# Synthesis of Chromeno[3,4-*b*]piperazines by an Enol-Ugi/Reduction/Cyclization Sequence

**DOI:** 10.3390/molecules26051287

**Published:** 2021-02-27

**Authors:** Ana Bornadiego, Ana G. Neo, Carlos F. Marcos

**Affiliations:** Laboratory of Bioorganic Chemistry & Membrane Biophysics (L.O.B.O.), Department of Organic and Inorganic Chemistry, University of Extremadura, 10003 Cáceres, Spain; bornadiegos@unex.es

**Keywords:** multicomponent reactions, isocyanides, peptidomimetics, heterocycles, piperazines, pyrazines, chromones, coumarins, enols, Ugi reaction

## Abstract

Keto piperazines and aminocoumarins are privileged building blocks for the construction of geometrically constrained peptides and therefore valuable structures in drug discovery. Combining these two heterocycles provides unique rigid polycyclic peptidomimetics with drug-like properties including many points of diversity that could be modulated to interact with different biological receptors. This work describes an efficient multicomponent approach to condensed chromenopiperazines based on the novel enol-Ugi reaction. Importantly, this strategy involves the first reported post-condensation transformation of an enol-Ugi adduct.

## 1. Introduction

Peptidomimetics are molecules structurally related to peptides that can interact at the same receptors of their prototypes [1,2]. They have attracted an enormous medical interest as they present similar, or sometimes opposite, effects to the equivalent peptide, but display more favorable pharmacological properties. Geometrically constrained peptidomimetics are an important class of peptide analogues containing cyclic structures that result in reduced conformational flexibility and usually well-defined secondary structures [3]. This frequently results in enhanced affinities for biological receptors, leading to improved biological activities [4]. In fact, many biologically active natural products present rigid peptide-like motives able to strongly bind to their target biomolecules.

Heterocycles are valuable building blocks to introduce defined structural constrains in peptide sequences [3,4]. Among the most attractive rigid peptidic scaffolds for drug discovery are piperazines [5,6,7,8,9,10], which are present in many alkaloids and pharmaceuticals that can bind to a wide range of receptors [11]. Additionally, 3-aminocoumarins are considered privileged structures, present in various biologically active natural didepsipeptides, such as bacterial antibiotics novobiocin [12,13,14], coumermycines [15] and cacibiocins [16], and marine-derived fungal metabolites trichodermamides [17,18,19].

Conventional methods for the synthesis of these compounds commonly consist in multi-step procedures, including several protection and deprotection reactions. In recent years, more convenient multicomponent approaches have been developed to straightforwardly generate diversely substituted constrained peptidomimetics in one or a few reaction steps. Thus, piperazines and pyrazines have been synthesized by modified Ugi condensations [20] and post-condensation transformations of Ugi [7,21,22,23] or Ugi–Smiles products [24,25]. Different multicomponent strategies have also been used in the synthesis of polycyclic coumarins [26].

**Figure 1:**
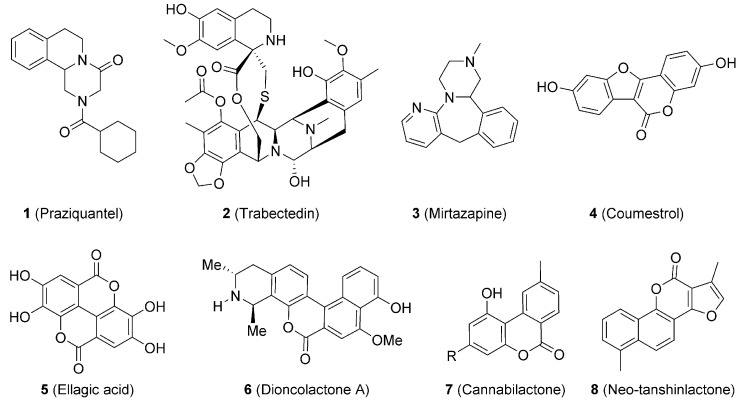
Some bioactive polycyclic pyrazines and coumarins.

Both condensed polycyclic piperazines [27] and coumarins [28] (Figure 1) are unique in terms of structure and properties, reaching larger areas of the chemical space of biologically relevant compounds, as well as a wider diversity of target proteins. Pharmacologically relevant polycyclic pyrazines include praziquantel (**1**), used as the primary medication for human schistosomiasis [29], trabectedin (**2**), a marine-derived orphan drug that has been approved for the treatment of soft tissue sarcomas [30], and antidepressant mirtazapine (**3**) [31]. On the other hand, many polycyclic coumarins have distinct biological activities. Notable examples are phytoestrogen coumestrol (**4**) [32], antioxidant and anticancer ellagic acid (**5**) [33], antimalarial dioncolactone (**6**) [34], cannabinoid agonists cannabilactones (**7**) [35] and neo-tanshinlactone (**8**), a natural compound known for its specificity and selectivity towards the breast cancer cells [36]. Thus, combining the coumarin and pyrazino scaffolds in the same structure opens new opportunities to develop molecules with novel and selective biological activities [37].

**Figure 2:**
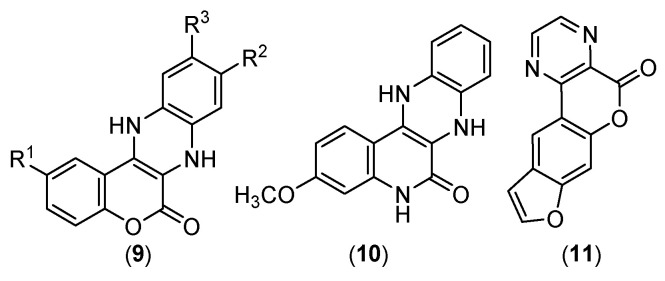
Some bioactive chromenopyrazines and quinolinopyrazines.

**Scheme 1:**
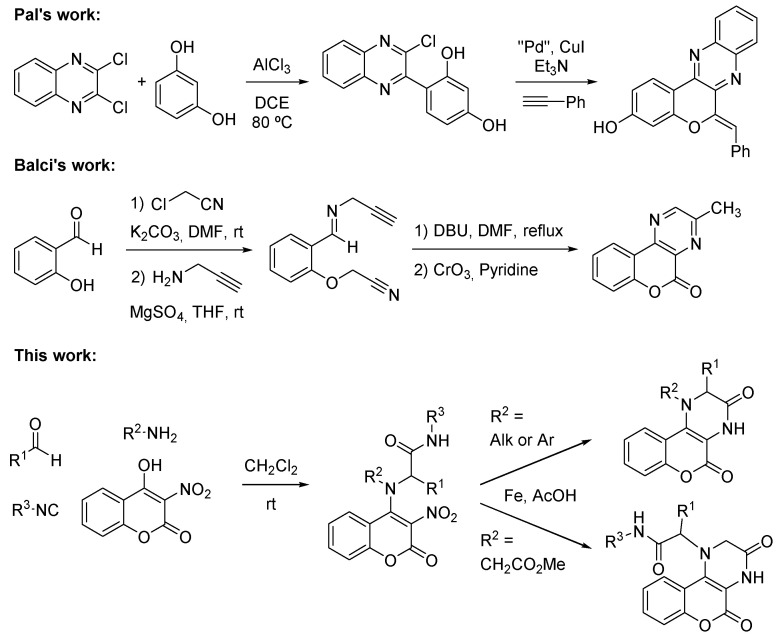
Representative syntheses of chromenopyrazines.

Reports on chromenopyrazines are scarce, but some interesting examples have been described. Thus, chromeno[3,4-*b*]pyrazines (**9**) [38] and the structural related pyrazino[2,3-*c*]quinolin-5(6*H*)-ones (**10**) [39] are known to have antimicrobial and anticancer activities. Additionally, furochromenopyrazines (**11**) present similar photobiological activities than psoralens (Figure 2) [40]. Although some syntheses of pyrazino[2,3-*c*]quinolin-5(6*H*)-ones have been reported [41,42], literature review reveals a limited number of known synthesis of chromenopyrazines (Scheme 1) [43,44]. For example, Pal and co-workers used an AlCl_3_-induced C–C bond formation followed by transition metal mediated coupling-cyclization to synthesize chromeno[4,3-*b*]quinoxalines [43]. More recently, the group of Balci reported a synthesis of chromenopyrazinone derivatives in four reaction steps, starting from salicylaldehyde [44]. These syntheses require the use of environmentally noxious transition metals and permit a limited diversity of products. Consequently, the development of simple and efficient synthetic methods is indispensable to further explore the chromenopyrazine structure.

We have previously used multicomponent reactions of isocyanides for the synthesis of peptide and pseudopeptide derivatives [45,46,47,48]. As part of this research line, here we report the multicomponent synthesis of chromeno[3,4-*b*]pyrazin-5-ones as geometrically restricted peptidomimetics.

## 2. Results and Discussion

**Scheme 2:**
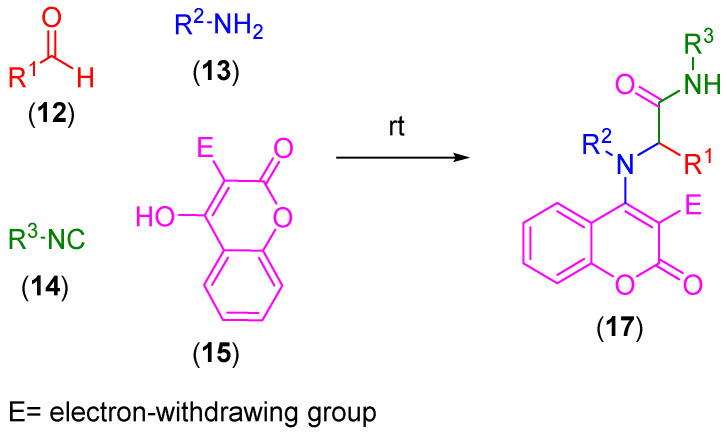
Enol-Ugi condensation of 4-hydroxycoumarin.

We have recently reported the effective multicomponent enol-Ugi condensation of enols (**15**), aldehydes (**12**), amines (**13**) and isocyanides (**14**) leading to polysubstituted heterocyclic enamines (**17**; Scheme 2) [49,50,51]. The enol-Ugi condensation of 4-hydroxy-3-nitro-coumarin (**15**) and cyclohexyl isocyanide (**14a**) with different amines (**13a**–**d**) and aldehydes (**12a**–**e**) or the corresponding preformed imines (**16a**–**k**) leads to aminoacylcoumarins (**17a**–**k**) in good to excellent yields (Table 1).

**Scheme 3:**
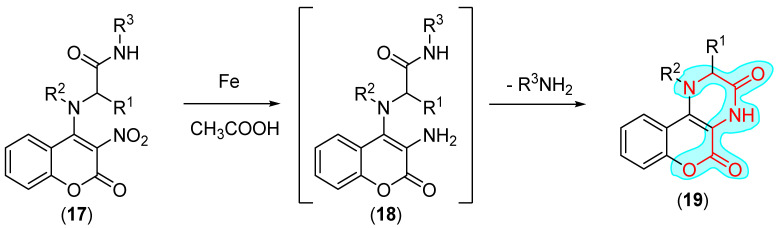
Postcondensation synthesis of chromenopyrazinones. Dipeptidic structure is shaded in light blue colour.

Reduction of the nitro group in the enol-Ugi adducts (**17a**–**k**) with iron in acetic acid takes place smoothly at room temperature to afford amino intermediates (**18a**–**k**) that are usually not isolated. In the case of enol-Ugi adducts derived from aliphatic amines (**17a**–**i**) the spontaneous intramolecular attack of the amine on the amide group generates a pyrazine ring (**19a**–**i**) with loss of cyclohexylamine (Scheme 3). Conversely, enol-Ugi adducts derived from aromatic amines (**17j**,**k**) give stable aminocoumarins (**18j**,**k**) that can be isolated. However, when the reduction/cyclization was carried out at 150 °C the corresponding chromenopyrazines (**19j**,**k**) were directly obtained (Table 1).

**Scheme 4:**
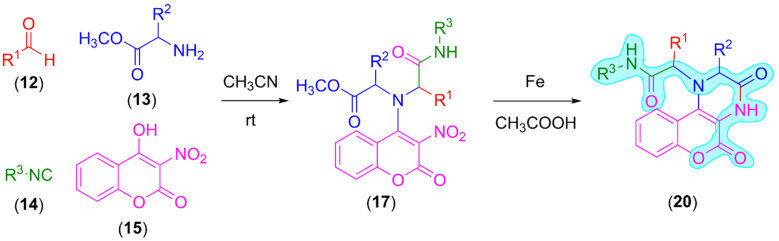
Synthesis of chromenopyrazine tripeptides. Tripeptidic structure is shaded in light blue colour.

A rigid dipeptidic structure is comprised in the pyrazine and pyranone rings of chromenopyrazines (**19**; Scheme 3). In order to extend the peptidic skeleton of these geometrically constrained dipeptides, we decided to use esters of amino acids as amino components of the enol-Ugi reaction (Scheme 4). Accordingly, the four-component reaction of 4-hydroxy-3-nitro-coumarin (**15**), different isocyanides (**14a**–**d**) and aldehydes (**12a**,**c**,**d**) with glycine methyl ester (**13e**) gave the corresponding adducts (**17l**–**r**) in good yields. The analogous condensation of β-alanine (**13f**) similarly gave adduct **17s** (Table 2).

Interestingly, in this case, reduction of the nitro group does not lead to cyclization by the attack on the amide, as with adducts **17a**–**k**. The attack of the amine occurs instead on the more reactive ester group derived from the glycine methyl ester, affording pyrazines **20l**–**r** (Scheme 4; Table 2). The amide group brought by the isocyanide component is thus preserved in the product delivering a new element of diversity, as different isocyanides can be used (Table 2). Rigid retropeptidic tripeptides are obtained in this manner. The peptide sequence could theoretically be grown from the isocyanide-derived amide to obtain peptides with an inverted rigid *N*-terminus.

On the other hand, the β-alanine-derived adduct (**17s**) cyclizes again by attack on the amide to give pyrazine **19s**. This reaction is more favorable than cyclization on the ester, as this would involve the formation of a seven-membered, instead of a six membered ring.

## 3. Materials and Methods

### 3.1. Starting Materials

Acetonitrile was dried by distillation over P_2_O_5_, immediately prior to use. Glacial acetic acid was purchased from commercial sources and used as received. Aldehydes (**12a**–**e**), amines (**13a**–**f**), isocyanides (**14a**–**d**; S.I. Figure S1), 4-hydroxycoumarin and iron powder are commercially available and were used without purification. 4-Hydroxy-3-nitrocoumarin (**15**) was prepared by nitration of 4-hydroxycoumarin [52]. Imines (**16a**–**k**; S.I. Figure S2) were synthesized using the standard procedure of mixing equimolar amounts of the corresponding aldehydes (**12**), amines (**13**) and anhydrous Na_2_SO_4_ in dry CH_2_Cl_2_ at room temperature for 24 h. [53,54] Evaporation of the solvent quantitatively yielded the imines (**16**) that were used in the Ugi reaction without further purification.

### 3.2. General Synthetic Techniques

Liquid reagents were measured using positive-displacement micropipettes with disposable tips and pistons. Thin layer chromatography was performed on aluminum plates, using 254 nm UV light or a mixture of *p*-anisaldehyde (2.5%), acetic acid (1%) and H_2_SO_4_ (3.4%) in 95% ethanol as developer.

### 3.3. Instrumentation

Melting points are uncorrected. IR spectra were recorded as KBr pellets. Proton and carbon-13 nuclear magnetic resonance (^1^H-NMR or ^13^C-NMR) spectra were obtained on a 500 MHz spectrometer. The assignments of signals in ^13^C-NMR were made by DEPT. High resolution mass spectra (HRMS) were recorded using a 6520 Accurate Mass QTOF LC/MS Spectrometer.

### 3.4. Synthesis and Characterization of the Ugi-Aducts

#### 3.4.1. Three-Component Condensation

Our previously reported procedure [51] was followed. Briefly, isocyanide (**14**, 0.5 mmol) and enol (**15**, 0.5 mmol) were successively added to a solution of imine (**16**, 0.5 mmol) in CH_2_Cl_2_ (1 mL), and the resulting mixture was stirred at 20 °C for 3 h. Removal of the solvent and purification by column chromatography (SiO_2_, gradient from 100% hexanes to hexanes–EtOAc, 7:3) gave the corresponding enamines **17a**–**k** (Table 1).

*2-(Benzyl(3-nitro-2-oxo-2H-chromen-4-yl)amino)-2-(2-bromophenyl)-N-cyclohexyl acetamide* (**17b**). Obtained from isocyanide **14a**, enol **15** and imine **16b**, from isocyanide **14a**, enol **15** and imine **16b**, as a yellow solid (260 mg, 88%); m.p. 165–167 °C; IR (cm^−1^) 3341, 3064, 2933, 2854, 1714, 1677, 1600, 1549, 1465, 1350, 1278, 1116, 1054, 925, 760; ^1^H-NMR (500 MHz, CDCl_3_) δ 8.14 (d, *J* = 8.0 Hz, 1H), 7.63–7.57 (m, 3H), 7.42 (dt, *J* = 7.5, 0.9 Hz, 1H), 7.40 (dt, *J* = 3.7, 1.0 Hz, 1H), 7.38–7.36 (m, 1H), 7.31 (dd, *J* = 8.3, 0.8 Hz, 1H), 7.28 (dd, *J* = 7.8, 1.4 Hz, 1H), 7.25–7.18 (m, 5H), 5.87 (s, 1H), 5.56 (d, *J* = 8.0 Hz, 1H), 4.77 (d, *J* = 14.8 Hz, 1H), 3.99 (d, *J* = 14.8 Hz, 1H), 3.82–3.73 (m, 1H), 1.93–1.54 (m, 5H), 1.39–0.97 (m, 5H); ^13^C-NMR (126 MHz, CDCl_3_) δ 167.45 (C), 155.12 (C), 153.00 (C), 152.48 (C), 135.14 (C), 134.33 (C), 133.96 (CH), 133.82 (CH), 130.99 (CH), 130.13 (CH), 129.02 (CH), 128.61 (CH), 128.44 (CH), 128.24 (CH), 127.49 (CH), 126.33 (C), 125.12 (CH), 118.45 (C), 117.90 (CH), 68.92 (CH), 54.76 (CH_2_), 49.26 (CH), 32.73 (CH_2_), 32.71 (CH_2_), 24.82 (CH_2_), 24.74 (CH_2_); MS (qTOF) *m*/*z* (%) 592 (M^+^ + 3, 28), 590 (M^+^ + 1, 30), 574 (42), 572 (44), 510 (98), 508 (100); HRMS (qTOF) Calcd for C_30_H_29_BrN_3_O_5_: 590.1296. Found: 590.1285.

*2-(Benzyl(3-nitro-2-oxo-2H-chromen-4-yl)amino)-N-cyclohexyl-2-(p-tolyl)acetamide* (**17c**). Obtained from isocyanide **14a**, enol **15** and imine **16c**, as a yellow solid (208 mg, 79%); m.p. 141–143 °C; IR (cm^−1^) 3369, 2928, 2853, 1729, 1681, 1601, 1549, 1451, 1403, 1350, 1116, 1054, 791; ^1^H-NMR (500 MHz, CDCl_3_) δ 8.04 (d, *J* = 8.1 Hz, 1H), 7.56 (dt, *J* = 7.3, 1.4 Hz, 1H), 7.31–7.26 (m, 2H), 7.24–7.11 (m, 9H), 5.78 (d, *J* = 8.1 Hz, 1H), 5.16 (s, 1H), 4.64 (d, *J* = 14.9 Hz, 1H), 4.19 (d, *J* = 14.9 Hz, 1H), 3.76–3.67 (m, 1H), 2.33 (s, 3H), 1.87–1.53 (m, 5H), 1.36–0.95 (m, 5H); ^13^C-NMR (126 MHz, CDCl_3_) δ 168.30 (C), 155.08 (C), 153.26 (C), 152.49 (C), 139.50 (C), 135.31 (C), 133.89 (CH), 131.78 (C), 129.90 (CH), 129.16 (CH), 129.04 (CH), 128.57 (C), 128.45 (CH), 128.39 (CH), 124.93 (CH), 118.38 (C), 117.65 (CH), 71.15 (CH), 55.96 (CH_2_), 48.99 (CH), 32.79 (CH_2_), 32.70 (CH_2_), 27.07 (CH_2_), 24.87 (CH_2_), 24.79 (CH_2_), 21.32 (CH_3_); MS (qTOF) *m*/*z* (%) 526 (M^+^ + 1, <5), 479 (10), 347 (100), 146 (54); HRMS (qTOF) Calcd for C_33_H_32_N_3_O_5_: 526.2326. Found: 526.2326.

*2-(Benzyl(3-nitro-2-oxo-2H-chromen-4-yl)amino)-N-cyclohexyl-2-(4-(trifluoromethyl) phenyl)acetamide* (**17d**). Obtained from isocyanide **14a**, enol **15** and imine **16d**, as a yellow solid (226 mg, 78%), m.p. 135–137 °C; IR (cm^−1^) 3365, 2932, 2855, 1730, 1684, 1603, 1550, 1324, 1168, 1127, 1068, 761, 699; ^1^H-NMR (500 MHz, CDCl_3_) δ 7.90 (d, *J* = 7.3 Hz, 1H), 7.61–7.57 (m, 3H), 7.52 (d, *J* = 8.2 Hz, 2H), 7.30 (d, *J* = 7.7 Hz, 2H), 7.24–7.18 (m, 3H), 7.10 (dd, *J* = 6.5, 1.6 Hz, 2H), 5.88 (d, *J* = 8.1 Hz, 1H), 5.16 (s, 1H), 4.63 (d, *J* = 14.7 Hz, 1H), 4.23 (d, *J* = 14.7 Hz, 1H), 3.71–3.61 (m, 1H), 1.84–1.55 (m, 5H), 1.35–0.93 (m, 5H); ^13^C-NMR (126 MHz, CDCl_3_) δ 167.30 (C), 154.79 (C), 153.12 (C), 152.48 (C), 139.01 (C), 134.71 (C), 134.23 (CH), 131.76 (C), 131.50 (C), 129.35 (CH), 129.29 (CH), 128.75 (CH), 128.70 (CH), 128.28 (CH), 126.15 (CH), 126.12 (CH), 125.05 (CH), 122.73 (C), 118.21 (C), 117.80 (CH), 70.56 (CH), 56.77 (CH_2_), 49.15 (CH), 32.68 (CH_2_), 32.60 (CH_2_), 25.41 (CH_2_), 24.82 (CH_2_), 24.75 (CH_2_); MS (qTOF) *m*/*z* (%) 580 (M^+^ + 1, 100), 391 (13), 309 (28); HRMS (qTOF) Calcd for C_31_H_29_F_3_N_3_O_5_: 580.2059. Found: 580.2059.

*2-((Benzo[d][1,3]dioxol-5-ylmethyl)(3-nitro-2-oxo-2H-chromen-4-yl)amino)-N-cyclohexyl-2-phenylacetamide* (**17e**). Obtained from isocyanide **14a**, enol **15** and imine **16e**, as a yellow solid (217 mg, 78%); m.p. 135–136 °C; IR (cm^−1^) 3369, 2930, 2853, 1728, 1681, 1601, 1549, 1504, 1489, 1446, 1401, 1347, 1249, 929, 761; ^1^H-NMR (500 MHz, CDCl_3_) δ 8.07 (dd, *J* = 8.1, 1.3 Hz, 1H), 7.56 (dt, *J* = 6.9, 1.4 Hz, 1H), 7.38–7.28 (m, 7H), 6.74 (d, *J* = 1.4 Hz, 1H), 6.61 (d, *J* = 7.9, Hz, 1H), 6.57 (dd, *J* = 8.0, 1.5 Hz, 1H), 5.89 (s, 2H), 5.78 (d, *J* = 8.1 Hz, 1H), 5.19 (s, 1H), 4.56 (d, *J* = 14.8 Hz, 1H), 4.09 (d, *J* = 14.8 Hz, 1H), 3.76–3.67 (m, 1H), 1.89–1.53 (m, 5H), 1.37–0.95 (m, 5H); ^13^C-NMR (126 MHz, CDCl_3_) δ 168.11 (C), 155.06 (C), 152.50 (C), 147.83 (C), 147.73 (C), 134.78 (C), 134.00 (CH), 129.52 (CH), 129.23 (CH), 129.12 (CH), 128.91 (C), 128.31 (CH), 125.06 (CH), 122.90 (CH), 118.25 (C), 117.71 (CH), 109.38 (CH), 108.16 (CH), 101.22 (CH_2_), 71.29 (CH), 55.71 (CH_2_), 49.04 (CH), 32.76 (CH_2_), 32.67 (CH_2_), 27.05 (CH_2_), 24.85 (CH_2_), 24.77 (CH_2_); MS (qTOF) *m*/*z* (%) 556 (M^+^ + 1, 672), 457 (20), 353 (100); HRMS (qTOF) Calcd for C_31_H_30_N_3_O_7_: 556.2084. Found: 556.2089.

*2-((Benzo[d][1,3]dioxol-5-ylmethyl)(3-nitro-2-oxo-2H-chromen-4-yl)amino)-N-cyclohexyl-2-(p-tolyl)acetamide* (**17f**). Obtained from isocyanide **14a**, enol **15** and imine **16f**, as a yellow solid (202 mg, 71%); m.p. 145–146 °C; IR (cm^−1^) 3424, 2929, 2853, 1728, 1679, 1601, 1549, 1489, 1446, 1249, 1039, 929, 761; ^1^H-NMR (500 MHz, CDCl_3_) δ 8.09 (d, *J* = 7.2 Hz, 1H), 7.58 (dt, *J* = 6.4, 1.3 Hz, 1H), 7.35–7.27 (m, 2H), 7.21 (d, *J* = 8.0 Hz, 2H), 7.14 (d, *J* = 7.9 Hz, 2H), 6.75 (s, 1H), 6.63–6.55 (m, 2H), 5.88 (s, 2H), 5.72 (d, *J* = 8.1 Hz, 1H), 5.16 (s, 1H), 4.55 (d, *J* = 14.8 Hz, 1H), 4.08 (d, *J* = 15.0 Hz, 1H), 3.77–3.68 (m, 1H), 2.33 (s, 3H), 1.88–1.54 (m, 5H), 1.35–0.95 (m, 5H); ^13^C-NMR (126 MHz, CDCl_3_) δ 168.36 (C), 153.15 (C), 152.55 (C), 147.84 (C), 147.71 (C), 139.54 (C), 133.92 (CH), 131.77 (C), 130.54 (C), 129.93 (CH), 129.07 (CH), 128.36 (CH), 125.02 (CH), 122.90 (CH), 118.36 (C), 118.32 (C), 117.69 (CH), 109.43 (CH), 108.15 (CH), 101.20 (CH_2_), 71.14 (CH), 55.58 (CH_2_), 49.04 (CH), 32.82 (CH_2_), 32.73 (CH_2_), 27.07 (CH_2_), 24.88 (CH_2_), 24.80 (CH_2_), 21.32 (CH_3_); MS (qTOF) *m*/*z* (%) 570 (M^+^ + 1, 70), 353 (100); HRMS (qTOF) Calcd for C_32_H_32_N_3_O_7_: 570.2240. Found: 570.2236.

*2-((Benzo[d][1,3]dioxol-5-ylmethyl)(3-nitro-2-oxo-2H-chromen-4-yl)amino)-N-cyclohexyl-2-(4-(trifluoromethyl)phenyl)acetamide* (**17g**). Obtained from isocyanide **14a**, enol **15** and imine **16g**, as a yellow solid (250 mg, 80%); m.p. 129–131 °C; IR (cm^−1^) 3362, 2931, 2854, 1727, 1683, 1603, 1550, 1490, 1447, 1324, 1250, 1167, 1127, 1068, 930, 761; ^1^H-NMR (500 MHz, CDCl_3_) δ 7.96 (dd, *J* = 8.1, 1.1 Hz, 1H), 7.64–7.56 (m, 3H), 7.52 (d, *J* = 8.2 Hz, 2H), 7.35–7.29 (m, 2H), 6.67 (d, *J* = 1.5 Hz, 1H), 6.62 (d, *J* = 7.9 Hz, 1H), 6.52 (dd, *J* = 8.0, 1.5 Hz, 1H), 5.90 (s, 2H), 5.82 (d, *J* = 8.0 Hz, 1H), 5.16 (s, 1H), 4.55 (d, *J* = 14.6 Hz, 1H), 4.16 (d, *J* = 14.6 Hz, 1H), 3.72–3.63 (m, 1H), 1.87–1.52 (m, 5H), 1.35–0.94 (m, 5H); ^13^C-NMR (126 MHz, CDCl_3_) δ 167.34 (C), 154.78 (C), 153.00 (C), 152.53 (C), 148.00 (C), 147.96 (C), 138.97 (C), 134.28 (CH), 129.35 (CH), 128.39 (C), 128.20 (CH), 126.17 (CH), 126.14 (CH), 125.14 (CH), 123.07 (CH), 118.16 (C), 117.85 (CH), 109.48 (CH), 108.28 (CH), 101.34 (CH_2_), 70.52 (CH), 56.42 (CH_2_), 49.19 (CH), 32.71 (CH_2_), 32.64 (CH_2_), 27.07 (CH_2_), 24.83 (CH_2_), 24.76 (CH_2_); MS (qTOF) *m*/*z* (%) 624 (M^+^ + 1, 35), 353 (100); HRMS (qTOF) Calcd for C_32_H_29_F_3_N_3_O_7_: 624.1958. Found: 624.1942.

*N-Cyclohexyl-2-(cyclohexyl(3-nitro-2-oxo-2H-chromen-4-yl)amino)-2-(p-tolyl)acetamide* (**17h**). Obtained from isocyanide **14a**, enol **15** and imine **16h**, as a pale orange solid (205 mg, 79%); m.p. 68–70 °C; IR (cm^−1^) 3415, 2931, 2855, 1736, 1662, 1605, 1540, 1451, 1374, 1276, 1111, 1055, 762; ^1^H-NMR (500 MHz, CDCl_3_) δ 7.98 (bs, 1H), 7.53 (dt, *J* = 7.2, 1.3 Hz, 1H), 7.32–7.28 (m, 3H), 7.21 (d, *J* = 7.6 Hz, 1H), 6.90 (d, *J* = 8.0 Hz, 2H), 6.53 (d, *J* = 5.8 Hz, 1H), 5.07 (s, 1H), 3.72–3.61 (m, 1H), 3.32 (tt, *J* = 11.6, 3.4 Hz, 1H), 2.17 (s, 3H), 2.10–0.81 (m, 20H); ^13^C-NMR (126 MHz, CDCl_3_) δ 170.63 (C), 154.63 (C), 153.53 (C), 151.63 (C), 138.79 (C), 133.82 (CH), 132.54 (C), 129.27 (CH), 129.21 (CH), 129.12 (CH), 128.70 (C), 127.78 (CH), 127.12 (CH), 124.57 (CH), 120.12 (C), 117.24 (CH), 72.17 (CH), 66.58 (CH), 48.60 (CH), 32.73 (CH_2_), 32.63 (CH_2_), 32.45 (CH_2_), 32.28 (CH_2_), 27.06 (CH_2_), 26.09 (CH_2_), 26.08 (CH_2_), 24.97 (CH_2_), 24.94 (CH_2_), 21.14 (CH_3_); MS (qTOF) *m*/*z* (%) 518 (M^+^ + 1, 30), 517 (35), 447 (100); HRMS (qTOF) Calcd for C_30_H_36_N_3_O_5_: 518.2655. Found: 518.2648.

*N-Cyclohexyl-2-(cyclohexyl(3-nitro-2-oxo-2H-chromen-4-yl)amino)-2-(4-(trifluoromethyl)phenyl)acetamide* (**17i**). Obtained from isocyanide **14a**, enol **15** and imine **16i**, as a pale orange solid (243 mg, 85%); m.p. 133–135 °C; IR (cm^−1^) 3386, 2933, 2856, 1737, 1681, 1606, 1541, 1325, 1167, 1127, 1068, 762; ^1^H-NMR (500 MHz, CDCl_3_) δ 7.93 (bs, 1H), 7.59 (d, *J* = 8.2 Hz, 2H), 7.54 (t, *J* = 7.3 Hz, 1H), 7.40 (d, *J* = 8.2 Hz, 2H), 7.32 (t, *J* = 7.7 Hz, 1H), 7.24 (d, *J* = 8.3 Hz, 1H), 6.55 (bs, 1H), 5.16 (s, 1H), 3.68–3.57 (m, 1H), 3.30 (tt, *J* = 11.6, 3.4 Hz, 1H), 2.09–1.53 (m, 10H), 1.37–0.95 (m, 10H); ^13^C-NMR (126 MHz, CDCl_3_) δ 169.44 (C), 154.32 (C), 152.78 (C), 151.79 (C), 140.01 (C), 134.25 (CH), 128.81 (CH), 125.58 (CH), 125.55 (CH), 124.86 (CH), 119.84 (C), 117.56 (CH), 72.08 (CH), 66.63 (CH), 48.84 (CH), 32.66 (CH_2_), 32.58 (CH_2_), 32.49 (CH_2_), 32.30 (CH_2_), 27.07 (CH_2_), 26.07 (CH_2_), 25.47 (CH_2_), 25.23 (CH_2_), 24.92 (CH_2_), 24.89 (CH_2_); MS (qTOF) *m*/*z* (%) 572 (M^+^ + 1, 69), 473 (100), 383 (17), 301 (171); HRMS (qTOF) Calcd for C_30_H_33_F_3_N_3_O_5_: 572.2372. Found: 572.2367.

*N-Cyclohexyl-2-(3,4-dimethoxyphenyl)-2-((3-nitro-2-oxo-2H-chromen-4-yl)(phenyl)amino) acetamide* (**17k**). Obtained from isocyanide **14a**, enol **15** and imine **16k**, as an orange solid (112 mg, 40%); m.p. 129–130 °C; IR (cm^−1^) 3403, 2931, 2853, 1740, 1681, 1604, 1517, 1451, 1373, 1265, 1148, 761; ^1^H-NMR (500 MHz, CDCl_3_) δ 7.67 (d, *J* = 7.0 Hz, 1H), 7.55 (dt, *J* = 6.1, 1.5 Hz, 1H), 7.29–7.20 (m, 5H), 7.03 (t, *J* = 7.4 Hz, 1H), 6.99 (d, *J* = 8.0 Hz, 2H), 6.76 (dd, *J* = 8.3, 2.1 Hz, 2H), 6.67 (d, *J* = 1.9 Hz, 2H), 6.61 (d, *J* = 8.4 Hz, 2H), 6.37 (d, *J* = 8.2 Hz, 2H), 5.50 (s, 2H), 3.87–3.78 (m, 2H), 3.77 (s, 3H), 3.58 (s, 3H), 1.94–1.52 (m, 5H), 1.39–0.94 (m, 5H); ^13^C-NMR (126 MHz, CDCl_3_) δ 168.15 (C), 153.89 (C), 152.90 (C), 150.26 (C), 149.83 (C), 148.68 (C), 144.87 (C), 134.24 (CH), 129.75 (CH), 128.78 (CH), 125.29 (CH), 124.44 (C), 123.13 (CH), 123.04 (CH), 118.23 (C), 117.81 (CH), 117.60 (CH), 112.72 (CH), 110.84 (CH), 69.97 (CH), 55.90 (CH_3_), 55.65 (CH_3_), 48.64 (CH), 32.83 (CH_2_), 32.43 (CH_2_), 25.43 (CH_2_), 24.71 (CH_2_), 24.58 (CH_2_); MS (qTOF) *m*/*z* (%) 558 (M^+^ + 1, <5), 478 (35), 328 (57); HRMS (qTOF) Calcd for C_31_H_32_N_3_O_7_: 558.2240. Found: 558.2234.

#### 3.4.2. Four-Component Condensation

Amine **13** (0.5 mmol) was added to a solution of aldehyde **12** (0.5 mmol) in of dry acetonitrile (1 mL). The resulting mixture was stirred for 15 min at rt and then isocyanide **14** (0.5 mmol) and enol **15** (0.5 mmol) were successively added. After 4 days stirring at room temperature, the reaction went to completion, as judged by tlc. Then 10% HCl (2 mL) was added, the mixture was washed with H_2_O (15 mL), extracted with CH_2_Cl_2_ (3 × 20 mL) and dried over Na_2_SO_4_. Removal of the solvent and purification by column chromatography (SiO_2_, gradient from 100% hexanes to hexanes–EtOAc, 7:3) gave the corresponding enamines **17l–s** (Table 2).

*Methyl N-(2-(cyclohexylamino)-2-oxo-1-phenylethyl)-N-(3-nitro-2-oxo-2H-chromen-4-yl)glycinate* (**17l**). Obtained from aldehyde **12a**, amine **13e**, isocyanide **14a** and enol **15**, as a pale orange solid (141 mg, 57%); m.p. 132–134 °C; IR (cm^−1^) 3359, 2931, 2854, 1730, 1681, 1606, 1554, 1452, 1212, 760; ^1^H-NMR (500 MHz, CDCl_3_) δ 8.12 (d, *J* = 8.1 Hz, 1H), 7.63 (t, *J* = 7.7 Hz, 1H), 7.44–7.33 (m, 5H), 5.90 (d, *J* = 8.0 Hz, 1H), 5.39 (s, 1H), 4.10 (d, *J* = 18.0 Hz, 1H), 3.80 (d, *J* = 18.0 Hz, 1H), 3.74–3.66 (m, 1H), 3.64 (s, 3H), 1.91–0.93 (m, 10H); ^13^C-NMR (126 MHz, CDCl_3_) δ 169.12 (C), 167.52 (C), 152.60 (C), 152.42 (C), 134.39 (C), 133.94 (CH), 129.57 (CH), 129.24 (CH), 128.91 (CH), 127.89 (CH), 125.15 (CH), 117.77 (CH), 70.24 (CH), 52.37 (CH_3_), 51.80 (CH_2_), 48.85 (CH), 32.60 (CH_2_), 32.53 (CH_2_), 26.94 (CH_2_), 25.33 (CH_2_), 24.73 (CH_2_), 24.66 (CH_2_); MS (qTOF) *m*/*z* (%) 494 (M^+^ + 1, 26), 423 (10), 305 (100); HRMS (qTOF) Calcd for C_26_H_28_N_3_O_7_: 494.1927. Found: 494.1915.

*Methyl N-(2-(cyclohexylamino)-2-oxo-1-(p-tolyl)ethyl)-N-(3-nitro-2-oxo-2H-chromen-4-yl)glycinate* (**17m**). Obtained from aldehyde **12c**, amine **13e**, isocyanide **14a** and enol **15**, as a pale yellow solid (122 mg, 48%); m.p. 114–116 °C; IR (cm^−1^) 3383, 2930, 2854, 1728, 1680, 1604, 1551, 1451, 1209, 1119, 1057, 759; ^1^H-NMR (500 MHz, CDCl_3_) δ 8.09 (dd, *J* = 6,99, 1.5 Hz, 1H), 7.64 (dt, *J* = 7.0, 1.4 Hz, 1H), 7.40–7.33 (m, 3H), 7.26–7.17 (m, 3H), 5.81 (d, *J* = 8.1 Hz, 1H), 5.70 (s, 1H), 4.23 (d, *J* = 18.3 Hz, 1H), 3.82–3.71 (m, 1H), 3,74 (d, *J* = 18.3 Hz, 1H), 3.66 (s, 3H), 2.32 (s, 3H), 1.95–1.54 (m, 5H), 1.38–0.95 (m, 5H); ^13^C-NMR (126 MHz, CDCl_3_) δ 169.15 (C), 168.14 (C), 155.19 (C), 152.67 (C), 152.61 (C), 137.90 (C), 134.06 (CH), 132.85 (C), 131.83 (CH), 129.58 (CH), 128.33 (CH), 127.79 (CH), 126.85 (CH), 125.21 (CH), 118.07 (CH), 117.30 (C), 66.50 (CH), 52.62 (CH_3_), 51.42 (CH_2_), 49.00 (CH), 32.78 (CH_2_), 32.69 (CH_2_), 25.43 (CH_2_), 24.87 (CH_2_), 24.81 (CH_2_), 19.81 (CH_3_); MS (qTOF) *m*/*z* (%) 508 (M^+^ + 1, 67), 319 (100); HRMS (qTOF) Calcd for C_27_H_30_N_3_O_7_: 508.2084. Found: 508.2071.

*Methyl N-(2-(cyclohexylamino)-2-oxo-1-(4-(trifluoromethyl)phenyl)ethyl)-N-(3-nitro-2-oxo-2H-chromen-4-yl)glycinate* (**17n**). Obtained from aldehyde **12d**, amine **13e**, isocyanide **14a** and enol **15**, as a pale yellow solid (146 mg, 52%); m.p. 113–115 °C; IR (cm^−1^) 3368, 2933, 2855, 1734, 1605, 1554, 1325, 1169, 1127, 1069, 760; ^1^H-NMR (500 MHz, CDCl_3_) δ 8.09 (dd, *J* = 8.1, 1.3 Hz, 1H), 7.68–7.62 (m, 3H), 7.57 (d, *J* = 8.2 Hz, 2H), 7.43–7.37 (m, 2H), 5.98 (d, *J* = 8.1 Hz, 1H), 5.42 (s, 1H), 4.14 (d, *J* = 17.9 Hz, 1H), 3.80 (d, *J* = 17.9 Hz, 1H), 3.73–3.63 (m, 1H), 3.66 (s, 3H), 1.90 –0.92 (m, 10H); ^13^C-NMR (126 MHz, CDCl_3_) δ 169.08, 166.89, 154.89, 152.76, 152.24, 138.60, 134.35, 132.66, 131.95, 131.69, 129.39, 127.70, 126.26, 125.47, 124.88, 122.71, 118.05, 117.39, 69.62, 52.62, 52.09, 49.17, 32.68, 32.63, 25.41, 24.83, 24.77; MS (qTOF) *m*/*z* (%) 568 (M^+^ + 1, 100), 373 (25); HRMS (qTOF) Calcd for C_27_H_27_F_3_N_3_O_7_: 562.1801. Found: 562.1793.

*Methyl N-(2-(tert-butylamino)-2-oxo-1-phenylethyl)-N-(3-nitro-2-oxo-2H-chromen-4-yl)glycinate* (**17o**). Obtained from aldehyde **12a**, amine **13e**, isocyanide **14b** and enol **15**, as a yellow solid (140 mg, 60%); m.p. 139–141 °C; IR (cm^−1^) 3378, 2969, 1685, 1605, 1554, 1456, 1365, 1213, 759; ^1^H-NMR (500 MHz, CDCl_3_) δ 8.10 (dd, *J* = 8.1, 1.2 Hz, 1H), 7.62 (t, *J* = 7.0 Hz, 1H), 7.44–7.29 (m, 7H), 5.84 (bs, 1H), 5.31 (s, 1H), 4.16 (d, *J* = 18.1 Hz, 1H), 3.78 (d, *J* = 18.1 Hz, 1H), 3.65 (s, 3H), 1.25 (s, 9H); ^13^C-NMR (126 MHz, CDCl_3_) δ 169.19 (C), 167.67 (C), 155.18 (C), 152.70 (C), 152.65 (C), 134.63 (C), 134.04 (CH), 129.63 (CH), 129.36 (CH), 129.04 (C), 128.96 (CH), 128.70 (C), 128.00 (CH), 125.24 (CH), 117.90 (CH), 70.68 (CH), 52.51 (CH_3_), 52.21 (C), 51.89 (CH_2_), 28.78 (CH_3_), 28.72 (CH_3_), 28.47 (CH_3_); MS (qTOF) *m*/*z* (%) 468 (M^+^ + 1, 15), 279 (100); HRMS (qTOF) Calcd for C_24_H_26_N_3_O_7_: 468.1771. Found: 468.1751.

*Methyl N-(3-nitro-2-oxo-2H-chromen-4-yl)-N-(2-oxo-2-(pentylamino)-1-phenylethyl) glycinate* (**17p**). Obtained from aldehyde **12a**, amine **13e**, isocyanide **14c** and enol **15**, as a yellow solid (92 mg, 38%); m.p. 72–74 °C; IR (cm^−1^) 3333, 2956, 1740, 1736, 1650, 1606, 1556, 1454, 1374, 1280, 1209, 1060, 761; ^1^H-NMR (500 MHz, CDCl_3_) δ 8.11 (dd, *J* = 8.1, 1.3 Hz, 1H), 7.63 (dt, *J* = 6.2, 1.4 Hz, 1H), 7.42–7.35 (m, 7H), 5.95 (t, *J* = 5.5 Hz, 1H), 5.40 (s, 1H), 4.16 (d, *J* = 17.9 Hz, 1H), 3.81 (d, *J* = 18.0 Hz, 1H), 3.65 (s, 3H), 3.25–3.17 (m, 2H), 1.45–1.36 (m, 2H), 1.28–1.20 (m, 2H), 1.20–1.11 (m, 2H), 0.83 (t, *J* = 7.2 Hz, 3H); ^13^C-NMR (126 MHz, CDCl_3_) δ 169.19 (C), 168.56 (C), 155.14 (C), 152.76 (C), 152.41 (C), 134.45 (C), 134.05 (CH), 129.75 (CH), 129.39 (CH), 129.06 (CH), 127.88 (CH), 125.34 (CH), 117.93 (CH), 117.60 (C), 70.23 (CH), 52.50 (CH_3_), 51.95 (CH_2_), 40.04 (CH_2_), 28.99 (CH_2_), 22.34 (CH_2_), 14.03 (CH_3_); MS (qTOF) *m*/*z* (%) 482 (M^+^ + 1, 100), 395 (37); HRMS (qTOF) Calcd for C_25_H_28_N_3_O_7_: 482.1927. Found: 482.1916.

*Methyl N-(2-(benzylamino)-2-oxo-1-phenylethyl)-N-(3-nitro-2-oxo-2H-chromen-4-yl)glycinate* (**17q**). Obtained from aldehyde **12a**, amine **13e**, isocyanide **14d** and enol **15**, as a yellow solid (118 mg, 47%); m.p. 145–147 °C; IR (cm^−1^) 3296, 2946, 1744, 1722, 1651, 1602, 1556, 1532, 1454, 1410, 1215, 1179, 1054, 759, 698; ^1^H-NMR (500 MHz, CDCl_3_) δ 8.10 (d, *J* = 7.2 Hz, 1H), 7.62 (dt, *J* = 7.1, 1.4 Hz, 1H), 7.42–7.36 (m, 5H), 7.34 (d, *J* = 7.8 Hz, 2H), 7.27–7.23 (m, 3H), 7.08 (dd, *J* = 6.4, 3.0 Hz, 2H), 6.32 (t, *J* = 5.6 Hz, 1H), 5.47 (s, 1H), 4.38 (dd, *J* = 5.8, 1.3 Hz, 2H), 4.14 (d, *J* = 18.0 Hz, 1H), 3.81 (d, *J* = 18.0 Hz, 1H), 3.62 (s, 3H); ^13^C-NMR (126 MHz, CDCl_3_) δ 1169.19 (C), 168.52 (C), 155.05 (C), 152.68 (C), 152.37 (C), 137.43 (C), 134.23 (C), 134.05 (CH), 129.82 (CH), 129.44 (CH), 129.07 (CH), 128.87 (CH), 127.81 (CH), 125.35 (CH), 117.93 (CH), 117.52 (C), 70.09 (CH), 52.49 (CH_3_), 51.93 (CH_2_), 43.97 (CH_2_); MS (qTOF) *m*/*z* (%) 502 (M^+^ + 1, 100), 415 (24), 299 (17); HRMS (qTOF) Calcd for C_27_H_24_N_3_O_7_: 502.1614. Found: 502.1602.

*Methyl N-(2-(benzylamino)-2-oxo-1-(p-tolyl)ethyl)-N-(3-nitro-2-oxo-2H-chromen-4-yl)glycinate* (**17r**). Obtained from aldehyde **12c**, amine **13e**, isocyanide **14d** and enol **15**, as a yellow solid (124 mg, 48%); m.p. 146–148 °C; IR (cm^−1^) 3294, 1747, 1720, 1655, 1601, 1554, 1530, 1452, 1436, 1368, 1209, 1178, 1053, 794; ^1^H-NMR (500 MHz, CDCl_3_) δ 8.05 (d, *J* = 7.4 Hz, 1H), 7.62 (t, *J* = 7.3 Hz, 1H), 7.38–7.20 (m, 10H), 7.14–7.11 (m, 2H), 6.26 (t, *J* = 5.6 Hz, 1H), 5.78 (s, 1H), 4.41 (d, *J* = 5.9 Hz, 2H), 4.23 (d, *J* = 18.3 Hz, 1H), 3.74 (d, *J* = 18.3 Hz, 1H), 3.64 (s, 3H), 2.29 (s, 3H); ^13^C-NMR (126 MHz, CDCl_3_) δ 169.11 (C), 169.04 (C), 155.06 (C), 152.66 (C), 152.49 (C), 137.93 (C), 137.51 (C), 134.03 (C), 132.57 (C), 131.89 (CH), 129.70 (CH), 128.92 (CH), 128.48 (CH), 127.87 (CH), 127.66 (CH), 126.9 (CH)3, 125.30 (CH), 118.09 (CH), 117.25 (C), 66.23 (CH), 52.58 (CH_3_), 51.45 (CH_2_), 44.00 (CH_2_), 19.77 (CH_3_); MS (qTOF) *m*/*z* (%) 516 (M^+^ + 1, 17), 429 (100), 299 (10); HRMS (qTOF) Calcd for C_28_H_26_N_3_O_7_: 516.1771. Found: 516.1782.

*Ethyl 3-((2-(cyclohexylamino)-2-oxo-1-phenylethyl)(3-nitro-2-oxo-2H-chromen-4-yl)amino)propanoate* (**17s**). Obtained from aldehyde **12a**, amine **13f**, isocyanide **14a** and enol **15**, as a yellow solid (131 mg, 50%); m.p. 130–131 °C; IR (cm^−1^) 3367, 2931, 2854, 1732, 1682, 1603, 1553, 1451, 1200, 1052, 762; ^1^H-NMR (500 MHz, CDCl_3_) δ 8.05 (dd, *J* = 8.1, 1.2 Hz, 1H), 7.65 (d, *J* = 7,3, 1.3 Hz, 1H), 7.45–7.34 (m, 7H), 5.99 (d, *J* = 8.0 Hz, 1H), 5.02 (s, 1H), 4.01 (q, *J* = 7.1 Hz, 2H), 3.83 (dt, *J* = 14.6, 7.2 Hz, 1H), 3.70–3.62 (m, 1H), 3.43–3.36 (m, 1H), 2.62 (dt, *J* = 17.0, 7.1 Hz, 1H), 2.44 (dt, *J* = 17.1, 6.2 Hz, 1H), 1.91 (d, *J* = 10.9 Hz, 1H), 1.75–1.52 (m, 5H), 1.36–1.16 (m, 3H), 1.13 (t, *J* = 9.0 Hz, 3H), 0.98 (ddd, *J* = 23.4, 12.1, 3.4 Hz, 1H); ^13^C-NMR (126 MHz, CDCl_3_) δ 171.43 (C), 167.65 (C), 155.05 (C), 153.46 (C), 152.85 (C), 134.86 (C), 134.03 (CH), 132.08 (C), 129.61 (CH), 129.32 (CH), 128.97 (CH), 127.89 (CH), 125.25 (CH), 118.04 (C), 117.89 (CH), 70.02 (CH), 61.03 (CH_2_), 49.14 (CH), 47.30 (CH_2_), 32.63 (CH_2_), 32.54 (CH_2_), 32.52 (CH_2_), 27.05 (CH_2_), 25.49 (CH_2_), 24.94 (CH_2_), 24.83 (CH_2_), 14.12 (CH_3_); MS (qTOF) *m*/*z* (%) 522 (M^+^ + 1, 100), 481 (17), 333 (20); HRMS (qTOF) Calcd for C_28_H_32_N_3_O_7_: 522.2240. Found: 522.2231.

#### 3.4.3. General Procedure for the Reduction of Nitro Derivatives **17a–s**

To a vigorously stirred solution of enol-Ugi adduct **17a**–**s** (0.4 mmol) in glacial acetic acid (8 mL), iron powder (9.6 mmol, 24 equiv) was added in one portion. The reaction mixture was stirred at rt for 2–4 h. Then water (50 mL) and dichloromethane (25 mL) were added. The unreacted iron was removed by filtration and the filtrate transferred to a separatory funnel. The phases were separated, and the aqueous layer extracted again with dichloromethane (25 mL). The combined organic extracts were washed with water (25 mL), saturated NaHCO_3_ (10 mL) and water again (25 mL), and then dried (Na_2_SO_4_) and evaporated to dryness. The residue was purified by flash column chromatography (SiO_2_, gradient from 100 % hexanes to hexanes–AcOEt 7:3) to give, depending on the case, chromeno[3,4-*b*]piperazines **19a**–**i,s**, aminocoumarins **18j**,**k** or chromeno[3,4-*b*]piperazines **20l**–**r** (Table 1 and Table 2).

##### Synthesis and Characterization of chromeno[3,4-b]piperazines **19a**–**i,s**

*1-Benzyl-2-phenyl-1,4-dihydro-2H-chromeno[3,4-b]pyrazine-3,5-dione* (**19a**). Obtained from **17a** as a pale yellow solid (130 mg, 85%); m.p. 183–185 °C; IR (cm^−1^) 3254, 1677, 1620, 1567, 1495, 1465, 1427, 1357, 1182, 1101, 1046, 746, 701; ^1^H-NMR (500 MHz, CDCl_3_) δ 7.98 (dd, *J* = 8.0, 1.3 Hz, 1H), 7.84 (bs, 1H), 7.53 (dt, *J* = 7.3, 1.5 Hz, 1H), 7.41 (t, *J* = 7.3 Hz, 2H), 7.39–7.30 (m, 8H), 7.28–7.23 (m, 2H), 4.98 (s, 1H), 4.87 (d, *J* = 15.2 Hz, 1H), 4.71 (d, *J* = 15.2 Hz, 1H); ^13^C-NMR (126 MHz, CDCl_3_) δ 163.37 (C), 156.63 (C), 150.85 (C), 135.88 (C), 135.67 (C), 130.46 (CH), 129.33 (CH), 129.02 (CH), 128.75 (CH), 128.47 (CH), 127.93 (CH), 126.04 (CH), 125.15 (CH), 123.34 (CH), 118.01 (CH), 116.55 (C), 112.25 (C), 63.91 (CH), 58.03 (CH_2_); HRMS (qTOF) Calcd for C_24_H_19_N_2_O_3_: 383.1396. Found: 383.1381.

*1-Benzyl-2-(2-bromophenyl)-1,4-dihydro-2H-chromeno[3,4-b]pyrazine-3,5-dione* (**19b**). Obtained from **17b** as a pale yellow solid (155 mg, 84%); m.p. 240–242 °C; IR (cm^−1^) 3195, 3085, 2938, 1700, 1688, 1625, 1495, 1467, 1392, 1346, 1094, 755, 699; ^1^H-NMR (500 MHz, CDCl_3_) δ 7.89 (bs, 1H), 7.74 (dd, *J* = 8.0, 1.1 Hz, 1H), 7.61 (dd, *J* = 7.7, 1.3 Hz, 1H), 7.48 (dt, *J* = 6.9, 1.3 Hz, 1H), 7.41–7.28 (m, 5H), 7.20–7.10 (m, 4H), 7.04 (dd, *J* = 7.5, 1.8 Hz, 1H), 5.33 (s, 1H), 5.03 (d, *J* = 14.7 Hz, 1H), 4.87 (d, *J* = 14.7 Hz, 1H); ^13^C-NMR (126 MHz, CDCl_3_) δ 162.69 (C), 156.79 (C), 150.85 (C), 136.26 (C), 135.91 (C), 135.69 (C), 133.90 (CH), 130.47 (CH), 130.30 (CH), 129.21 (CH), 128.91 (CH), 128.33 (CH), 128.16 (CH), 128.08 (CH), 124.93 (CH), 124.53 (C), 124.12 (CH), 117.82 (CH), 116.47 (C), 111.90 (C), 64.07 (CH_2_), 59.43 (CH); HRMS (qTOF) Calcd for C_24_H_18_BrN_2_O_3_: 461.0501. Found: 461.0473.

*1-Benzyl-2-(p-tolyl)-1,4-dihydro-2H-chromeno[3,4-b]pyrazine-3,5-dione* (**19c**). Obtained from **17c** as a white solid (124 mg, 78%); m.p. 170–172 °C; IR (cm^−1^) 3396, 3272, 1692, 1619, 1495, 1363, 1206, 1113, 755; ^1^H-NMR (500 MHz, CDCl_3_) δ 7.96 (dd, *J* = 8.0, 1.3 Hz, 1H), 7.79 (bs, 1H), 7.52 (dt, *J* = 7.4, 1.4 Hz, 1H), 7.43–7.30 (m, 7H), 7.23 (d, *J* = 8.1 Hz, 2H), 7.06 (d, *J* = 8.0 Hz, 2H), 4.93 (s, 1H), 4.86 (d, *J* = 15.2 Hz, 1H), 4.69 (d, *J* = 15.2 Hz, 1H), 2.26 (s, 3H); ^13^C-NMR (126 MHz, CDCl_3_) δ 163.50 (C), 156.63 (C), 150.86 (C), 138.32 (C), 135.97 (C), 135.86 (C), 132.76 (C), 130.38 (CH), 129.71 (CH), 129.32 (CH), 128.71 (CH), 127.95 (CH), 125.99 (CH), 125.09 (CH), 123.37 (CH), 117.98 (CH), 116.65 (C), 112.29 (C), 63.79 (CH), 57.99 (CH_2_), 21.12 (CH_3_); MS (qTOF) *m*/*z* (%) 397 (M^+^ + 1, 100), 337 (30); HRMS (qTOF) Calcd for C_25_H_21_N_2_O_3_: 397.1552. Found: 397.1544.

*1-Benzyl-2-(4-(trifluoromethyl)phenyl)-1,4-dihydro-2H-chromeno[3,4-b]pyrazine-3,5-dione* (**19d**). Obtained from **17d** as a pale yellow solid (160 mg, 89%); m.p. 172–174 °C; IR (cm^−1^) 3438, 3260, 1684, 1620, 1498, 1469, 1414, 1361, 1327, 1169, 1115, 1069, 752, 732; ^1^H-NMR (500 MHz, CDCl_3_) δ 7.99 (d, *J* = 6.8 Hz, 1H), 7.84 (bs, 1H), 7.57–7.48 (m, 5H), 7.44 (d, *J* = 8.5 Hz, 2H), 7.39–7.30 (m, 5H), 5.02 (s, 1H), 4.84 (d, *J* = 15.1 Hz, 1H), 4.70 (d, *J* = 15.1 Hz, 1H); ^13^C-NMR (126 MHz, CDCl_3_) δ 162.77 (C), 156.50 (C), 150.88 (C), 139.60 (C), 135.56 (C), 135.42 (C), 130.73 (CH), 129.45 (CH), 128.97 (CH), 128.00 (CH), 126.53 (CH), 126.06 (CH), 126.03 (C), 125.38 (CH), 123.11 (CH), 118.16 (CH), 116.42 (C), 112.72 (C), 63.64 (CH), 58.15 (CH_2_); MS (qTOF) *m*/*z* (%) 451 (M^+^ + 1, 95), 391 (100); HRMS (qTOF) Calcd for C_25_H_18_F_3_N_2_O_3_: 451.1270. Found: 451.1264.

*1-(Benzo[d][1,3]dioxol-5-ylmethyl)-2-phenyl-1,4-dihydro-2H-chromeno[3,4-b]pyrazine-3,5-dione* (**19e**). Obtained from **17e** as a pale yellow solid (147 mg, 86%); m.p. 174–176 °C; IR (cm^−1^) 3264, 1692, 1626, 1500, 1445, 1375, 1354, 1319, 1253, 1180, 1106, 1039, 926, 851, 747, 704; ^1^H-NMR (500 MHz, CDCl_3_) δ 7.97 (d, *J* = 4.0 Hz, 1H), 7.95 (d, *J* = 1.2 Hz, 1H), 7.53 (dt, *J* = 7.0 Hz, 1.4 Hz, 1H), 7.41 (d, *J* = 7.8 Hz, 2H), 7.36 (dd, *J* = 7.0, 1.5 Hz, 2H), 7.28–7.22 (m, 3H), 6.80–6.75 (m, 3H), 5.96 (s, 2H), 5.00 (s, 1H), 4.79 (d, *J* = 14.9 Hz, 1H), 4.59 (d, *J* = 14.9 Hz, 1H); ^13^C-NMR (126 MHz, CDCl_3_) δ 163.44 (C), 156.59 (C), 150.80 (C), 148.52 (C), 148.02 (C), 135.73 (C), 135.65 (C), 130.41 (CH), 129.55 (C), 128.99 (CH), 128.42 (CH), 125.99 (CH), 125.09 (CH), 123.32 (CH), 121.76 (CH), 117.98 (CH), 116.48 (C), 112.21 (C), 108.78 (CH), 108.06 (CH), 101.46 (CH_2_), 63.46 (CH), 57.69 (CH_2_); MS (qTOF) *m*/*z* (%) 427 (M^+^ + 1, 100), 427 (15), 274 (18); HRMS (qTOF) Calcd for C_25_H_19_N_2_O_5_: 427.1294. Found: 427.1293.

*1-(Benzo[d][1,3]dioxol-5-ylmethyl)-2-(p-tolyl)-1,4-dihydro-2H-chromeno[3,4-b]pyrazine-3,5-dione* (**19f**). Obtained from **17f** as a pale yellow solid (151 mg, 86%); m.p. 213–215 °C; IR (cm^−1^) 3408, 3192, 1715, 1677, 1625, 1502, 1419, 1326, 1298, 1109, 1036, 805, 751; ^1^H-NMR (500 MHz, CDCl_3_) δ 7.93 (dd, *J* = 8.0, 1.2 Hz, 1H), 7.76 (bs, 1H), 7.52 (dt, *J* = 7.3, 1.5 Hz, 1H), 7.43–7.36 (m, 2H), 7.23 (d, *J* = 8.0 Hz, 2H), 7.06 (d, *J* = 8.0 Hz, 2H), 6.77 (d, *J* = 0.8 Hz, 2H), 6.76 (s, 1H), 5.96 (d, *J* = 0.9 Hz, 2H), 4.94 (s, 1H), 4.77 (d, *J* = 14.9 Hz, 1H), 4.57 (d, *J* = 14.9 Hz, 1H); ^13^C-NMR (126 MHz, CDCl_3_) δ 163.52 (C), 156.64 (C), 150.85 (C), 148.57 (C), 148.06 (C), 138.34 (C), 135.77 (C), 132.68 (C), 130.42 (CH), 129.73 (CH), 129.64 (C), 125.94 (CH), 125.09 (CH), 123.38 (CH), 121.78 (CH), 118.02 (CH), 116.57 (C), 112.16 (C), 108.82 (CH), 108.08 (CH), 101.50 (CH_2_), 63.35 (CH), 57.69 (CH_2_), 21.14 (CH_3_); MS (qTOF) *m*/*z* (%) 441 (M^+^ + 1, 100), 400 (20), 281(30); HRMS (qTOF) Calcd for C_26_H_21_N_2_O_5_: 441.4630. Found: 441.1449.

*1-(Benzo[d][1,3]dioxol-5-ylmethyl)-2-(4-(trifluoromethyl)phenyl)-1,4-dihydro-2H-chromeno[3,4-b]pyrazine-3,5-dione* (**19g**). Obtained from **17g** as a pale yellow solid (148 mg, 75%); m.p. 190-192 °C; IR (cm^−1^) 3080, 2918, 1685, 1619, 1493, 1412, 1329, 1241, 1114, 1068, 1041, 998, 756; ^1^H-NMR (500 MHz, CDCl_3_) δ 7.96 (dd, *J* = 6.8, 1.6 Hz, 1H), 7.81 (bs, 2H), 7.58–7.48 (m, 5H), 7.46–7.41 (m, 2H), 6.80–6.75 (m, 3H), 5.97 (dd, *J* = 2.5, 1.3 Hz, 2H), 5.03 (s, 1H), 4.75 (d, *J* = 14.8 Hz, 1H), 4.58 (d, *J* = 14.8 Hz, 1H); ^13^C-NMR (126 MHz, CDCl_3_) δ 162.80 (C), 156.49 (C), 150.87 (C), 148.71 (C), 148.29 (C), 139.61 (C), 135.30 (C), 130.72 (CH), 129.22 (C), 126.51 (CH), 126.07 (CH), 126.04 (CH), 125.35 (CH), 123.11 (CH), 121.91 (CH), 118.18 (CH), 116.38 (C), 112.69 (C), 108.92 (CH), 108.10 (CH), 101.59 (CH_2_), 63.28 (CH), 57.89 (CH_2_); MS (qTOF) *m*/*z* (%) 495 (M^+^ + 1, 100), 339 (30), 353 (65); HRMS (qTOF) Calcd for C_26_H_18_F_3_N_2_O_5_: 495.1168. Found: 495.1157.

*1-Cyclohexyl-2-(p-tolyl)-1,4-dihydro-2H-chromeno[3,4-b]pyrazine-3,5-dione* (**19h**). Obtained from **17h** as a pale yellow solid (107 mg, 69%); m.p. 249–251 °C; IR (cm^−1^) 3444, 2931, 2853, 1692, 1622, 1495, 1406, 1335, 1102, 999, 757; ^1^H-NMR (500 MHz, CDCl_3_) δ 7.90 (bs, 1H), 7.74 (dd, *J* = 7.9, 1.4 Hz, 1H), 7.49 (dt, *J* = 6.0, 1.5 Hz, 1H), 7.42–7.36 (m, 2H), 7.28 (d, *J* = 8.0 Hz, 2H), 7.05 (d, *J* = 8.0 Hz, 2H), 5.17 (s, 1H), 3.77 (tt, *J* = 12.0, 3.7 Hz, 1H), 2.31 (d, *J* = 13.3 Hz, 1H), 2.25 (s, 3H), 1.95 (d, *J* = 13.2 Hz, 1H), 1.83–1.09 (m, 8H); ^13^C-NMR (126 MHz, CDCl_3_) δ 164.43 (C), 156.66 (C), 150.87 (C), 138.08 (C), 136.15 (CH), 133.12 (C), 130.15 (C), 129.60 (CH), 125.84 (CH), 124.98 (CH), 123.36 (CH), 117.92 (CH), 117.05 (C), 112.33 (C), 62.44 (CH), 58.98 (CH), 31.92 (CH_3_), 31.76 (CH_3_), 26.07 (CH_3_), 25.98 (CH_3_), 25.24 (CH_3_), 21.10 (CH_3_); MS (qTOF) *m*/*z* (%) 389 (M^+^ + 1, 100), 348 (31), 255 (29); HRMS (qTOF) Calcd for C_24_H_25_N_2_O_3_: 389.1865. Found: 389.1866.

*1-Cyclohexyl-2-(4-(trifluoromethyl)phenyl)-1,4-dihydro-2H-chromeno[3,4-b]pyrazine-3,5-dione* (**19i**). Obtained from **17i** as a pale yellow solid (127 mg, 72%); m.p. 113–115 °C; IR (cm^−1^) 3427, 2932, 2856, 1692, 1622, 1411, 1326, 1165, 1125, 1069, 755; ^1^H-NMR (500 MHz, CDCl_3_) δ 8.00 (bs, 1H), 7.75 (dd, *J* = 7.9, 1.2 Hz, 1H), 7.57–7.50 (m, 6H), 7.45–7.36 (m, 2H), 5.24 (s, 1H), 3.79 (tt, *J* = 12.0, 3.6 Hz, 1H), 2.30 (d, *J* = 12.6 Hz, 1H), 1.96 (d, *J* = 13.1 Hz, 1H), 1.85–1.58 (m, 5H), 1.29–1.11 (m, 3H); ^13^C-NMR (126 MHz, CDCl_3_) δ 163.73 (C), 156.52 (C), 150.91 (C), 140.21 (C), 135.73 (C), 130.46 (CH), 126.42 (CH), 125.96 (CH), 125.93 (CH), 125.20 (CH), 123.11 (CH), 118.10 (CH), 116.80 (C), 112.54 (C), 62.62 (CH), 59.06 (CH), 31.86 (CH_2_), 31.73 (CH_2_), 27.07 (CH_2_), 25.95 (CH_2_), 25.21 (CH_2_); MS (qTOF) *m*/*z* (%) 443 (M^+^ + 1, 100), 301 (<5); HRMS (qTOF) Calcd for C_24_H_22_F_3_N_2_O_3_: 443.1583. Found: 443.1578.

*Ethyl 3-(3,5-dioxo-2-phenyl-3,4-dihydro-2H-chromeno[3,4-b]pyrazin-1(5H)-yl)propanoate* (**19s**). Obtained from **17s** as a white solid (133 mg, 85%); m.p. 145–147 °C; IR (cm^−1^) 3231, 1733, 1680, 1619, 1498, 1460, 1372, 1188, 1126, 1021, 757; ^1^H-NMR (500 MHz, CDCl_3_) δ 7.99 (s, 1H), 7.89 (dd, *J* = 7.9, 1.1 Hz, 1H), 7.52 (dt, *J* = 7.3, 1.2 Hz, 1H), 7.43–7.36 (m, 4H), 7.29–7.24 (m, 3H), 5.09 (s, 1H), 4.17–4.06 (m, 3H), 3.89 (ddd, *J* = 14.7, 8.2, 6.4 Hz, 1H), 2.79 (ddd, *J* = 14.6, 8.1, 6.4 Hz, 1H), 2.73–2.63 (m, 1H), 1.19 (t, *J* = 7.1 Hz, 3H); ^13^C-NMR (126 MHz, CDCl_3_) δ 170.71 (C), 163.61 (C), 156.53 (C), 150.72 (C), 135.51 (C), 135.15 (C), 130.45 (CH), 129.06 (CH), 128.57 (CH), 125.81 (CH), 125.15 (CH), 123.39 (CH), 117.98 (CH), 116.43 (C), 112.23 (C), 64.73 (CH), 61.31 (CH_2_), 50.18 (CH_2_), 33.96 (CH_2_), 14.15 (CH_3_); HRMS (qTOF) Calcd for C_22_H_21_N_2_O_5_: 393.1450. Found: 393.1444.

##### Synthesis and Characterization of Aminocoumarins **18j**,**k**

*2-((3-Amino-2-oxo-2H-chromen-4-yl)(phenyl)amino)-N-cyclohexyl-2-phenylacetamide* (**18j**). Obtained from **17j** as a white solid (99 mg, 53%); m.p. 203–205 °C; IR (cm^−1^) 3466, 2933, 2852, 1719, 1635, 1600, 1556, 1497, 1455, 1177, 746; ^1^H-NMR (500 MHz, CDCl_3_) δ 7.27–7.18 (m, 4H), 7.11–7.05 (m, 3H), 7.04–6.96 (m, 4H), 6.83 (t, *J* = 7.3 Hz, 1H), 6.64 (d, *J* = 8.1 Hz, 2H), 6.21 (bs, 2H), 5.65 (d, *J* = 8.1 Hz, 1H), 5.51 (s, 1H), 3.93–3.83 (m, 1H), 2.15–0.92 (m, 10H); ^13^C-NMR (126 MHz, CDCl_3_) δ 171.49 (C), 160.18 (C), 147.29 (C), 145.30 (C), 134.85 (C), 132.81 (C), 129.70 (CH), 129.48 (CH), 129.42 (CH), 128.12 (CH), 125.71 (CH), 123.94 (CH), 122.65 (CH), 121.88 (C), 119.66 (C), 118.93 (CH), 115.93 (CH), 112.72 (CH), 68.52 (CH), 49.44 (CH), 33.19 (CH_2_), 32.69 (CH_2_), 25.55 (CH_2_), 24.98 (CH_2_), 24.84 (CH_2_); MS (qTOF) *m*/*z* (%) 468 (M^+^ + 1, 5, 407 (18), 369 (100); HRMS (qTOF) Calcd for C_29_H_30_N_3_O_3_: 468.2287. Found: 468.2285.

*2-((3-Amino-2-oxo-2H-chromen-4-yl)(phenyl)amino)-N-cyclohexyl-2-(3,4-dimethoxyphenyl)acetamide* (**18k**). Obtained from **17k** as a white solid (163 mg, 77%); m.p. 243–245 °C; IR (cm^−1^) 3439, 3326, 2938, 2850, 1715, 1637, 1599, 1539, 1518, 1464, 1253, 1176, 1152, 1028, 763, 749; ^1^H-NMR (500 MHz, CDCl_3_) δ 7.20 (t, *J* = 7.9 Hz, 2H), 7.11–7.06 (m, 2H), 6.98 (d, *J* = 3.5 Hz, 2H), 6.82–6.78 (m, 2H), 6.74 (s, 1H), 6.61 (d, *J* = 8.2 Hz, 2H), 6.50 (d, *J* = 8.3 Hz, 1H), 6.20 (bs, 2H), 5.60 (d, *J* = 8.1 Hz, 1H), 5.44 (s, 1H), 3.90–3.80 (m, 1H), 3.71 (s, 3H), 3.52 (s, 3H), 2.08–0.79 (m, 10H); ^13^C-NMR (126 MHz, CDCl_3_) δ 171.68 (C), 160.15 (C), 149.69 (C), 148.24 (C), 147.43 (C), 145.24 (C), 134.75 (C), 129.71 (CH), 125.75 (CH), 124.96 (CH), 123.95 (CH), 122.57 (CH), 121.99 (C), 119.86 (CH), 118.89 (CH), 116.26 (CH), 112.65 (CH), 110.32 (CH), 68.09 (CH), 55.87 (CH_3_), 55.79 (CH_3_), 49.43 (CH), 33.21 (CH_2_), 32.77 (CH_2_), 29.84 (CH_2_), 24.98 (CH_2_), 24.86 (CH_2_); MS (qTOF) *m*/*z* (%) 528 (M^+^ + 1, 100), 369 (25), 276 (13); HRMS (qTOF) Calcd for C_31_H_34_N_3_O_5_: 528.2498. Found: 528.2490.

##### Synthesis and Characterization of chromeno[3,4-b]piperazines **20l-r**

*N-cyclohexyl-2-(3,5-dioxo-3,4-dihydro-2H-chromeno[3,4-b]pyrazin-1(5H)-yl)-2-phenyl acetamide* (**20l**). Obtained from **17l** as a pale yellow solid (121 mg, 70%); m.p. 240–242 °C; IR (cm^−1^) 3268, 2931, 2854, 1703, 1818, 1544, 1496, 1451, 1382, 1111, 752; ^1^H-NMR (500 MHz, CDCl_3_) δ 7.63 (dd, *J* = 8.0, 1.2 Hz, 1H), 7.54 (bs, 1H), 7.51 (dt, *J* = 7.1, 1.4 Hz, 1H), 7.43–7.39 (m, 4H), 7.34–7.29 (m, 3H), 5.79 (d, *J* = 8.0 Hz, 1H), 5.37 (s, 1H), 3.98–3.90 (m, 1H), 3.92 (s, 3H), 2.03–1.90 (m, 2H), 1.74–1.57 (m, 4H), 1.45–1.07 (m, 4H.; ^13^C-NMR (126 MHz, CDCl_3_) δ 167.85 (C), 163.00 (C), 156.92 (C), 150.63 (C), 135.72 (C), 135.01 (C), 130.32 (CH), 129.52 (CH), 128.50 (CH), 125.06 (CH), 122.96 (CH), 118.02 (CH), 116.33 (C), 114.08 (C), 69.14 (CH), 49.49 (CH_2_), 49.05 (CH), 33.17 (CH_2_), 33.10 (CH_2_), 25.51 (CH_2_), 24.89 (CH_2_), 24.85 (CH_2_); MS (qTOF) *m*/*z* (%) 432 (M^+^ + 1, 100), 305 (<5), 234 (<5); HRMS (qTOF) Calcd for C_25_H_26_N_3_O_4_: 432.1923. Found: 432.1914.

*N-cyclohexyl-2-(3,5-dioxo-3,4-dihydro-2H-chromeno[3,4-b]pyrazin-1(5H)-yl)-2-(p-tolyl)acetamide* (**20m**). Obtained from **17m** as a pale yellow solid (98 mg, 55%); m.p. 245–247 °C (dec.); IR (cm^−1^) 3400, 3280, 2930, 2849, 1701, 1651, 1618, 1561, 1439, 1412, 1347, 1108, 751, 732; ^1^H-NMR (500 MHz, CDCl_3_) δ 7.67 (bs, 1H), 7.47 (t, *J* = 7.2 Hz, 1H), 7.44–7.37 (m, 3H), 7.35–7.31 (m, 2H), 7.26–7.19 (m, 2H), 5.72 (d, *J* = 8.1 Hz, 1H), 5.59 (s, 1H), 4.09 (d, *J* = 16.7 Hz, 1H), 3.98 (d, *J* = 16.7 Hz, 1H), 3.95–3.87 (m, 1H), 1.98–0.95 (m, 10H); ^13^C-NMR (126 MHz, CDCl_3_) δ 168.26 (C), 162.61 (C), 156.91 (C), 150.61 (C), 137.43 (C), 136.82 (C), 133.84 (C), 131.87 (CH), 130.36 (CH), 129.58 (CH), 128.28 (CH), 127.23 (CH), 124.88 (CH), 123.08 (CH), 118.05 (CH), 116.39 (C), 111.84 (C), 67.33 (CH), 49.97 (CH), 49.08 (CH_2_), 33.00 (CH_2_), 25.47 (CH_2_), 24.81 (CH_2_), 19.44 (CH_3_); MS (qTOF) *m*/*z* (%) 446 (M^+^ + 1, 11), 319 (100); HRMS (qTOF) Calcd for C_26_H_28_N_3_O_4_: 446.2080. Found: 446.2074.

*N-cyclohexyl-2-(3,5-dioxo-3,4-dihydro-2H-chromeno[3,4-b]pyrazin-1(5H)-yl)-2-(4-(trifluoromethyl)phenyl)acetamide* (**20n**). Obtained from **17n** as a pale yellow solid (162 mg, 81%); m.p. 249–251 °C (dec.); IR (cm^−1^) 3313, 3266, 2930, 2855, 1704, 1688, 1620, 1549, 1497, 1487, 1326, 1167, 1126, 1068, 824, ^1^H-NMR (500 MHz, CDCl_3_) δ 7.68 (d, *J* = 8.2 Hz, 1H), 7.60 (bs, 1H), 7.58 (dd, *J* = 8.1, 1.2 Hz, 1H), 7.53 (t, *J* = 7.8 Hz, 1H), 7.45–7.34 (m, 3H), 7.39–7.34 (m, 1H), 5.88 (d, *J* = 4.7 Hz, 1H), 5.37 (s, 1H), 3.98–3.88 (m, 1H), 3.91 (d, *J* = 2.9 Hz, 2H), 2.04–1.09 (m, 10H); ^13^C-NMR (126 MHz, CDCl_3_) δ 167.11 (C), 162.84 (C), 156.76 (C), 150.56 (C), 138.72 (C), 134.97 (C), 130.56 (CH), 128.98 (CH), 127.12 (C), 126.43 (CH), 125.26 (CH), 122.53 (CH), 118.18 (CH), 116.04 (C), 114.70 (C), 68.39 (CH), 49.41 (CH_2_), 49.20 (CH), 33.18 (CH_2_), 33.11 (CH_2_), 25.44 (CH_2_), 24.88 (CH_2_); MS (qTOF) *m*/*z* (%) 522 (M^+^ + Na^+^, 100), 500 (M^+^ + 1, 54), 429 (10); HRMS (qTOF) Calcd for C_26_H_25_F_3_N_3_O_4_: 500.1797. Found: 500.1782.

*N-(tert-butyl)-2-(3,5-dioxo-3,4-dihydro-2H-chromeno[3,4-b]pyrazin-1(5H)-yl)-2-phenylacetamide* (**20o**). Obtained from **17o** as a pale yellow solid (94 mg, 58%); m.p. 258–260 °C (dec.); IR (cm^−1^) 3328, 3269, 2966, 2931, 1680, 1619, 1561, 1496, 1466, 1365, 1288, 1111, 750; ^1^H-NMR (500 MHz, CDCl_3_) δ .59 (dd, *J* = 8.0, 1.2 Hz, 1H), 7.53–7.49 (m, 2H), 7.44–7.40 (m, 4H), 7.34–7.29 (m, 3H), 5.71 (bs, 1H), 5.31 (s, 1H), 3.96 (q, *J* = 16.9 Hz, 2H), 1.41 (s, 9H); ^13^C-NMR (126 MHz, CDCl_3_) δ 168.11 (C), 163.00 (C), 156.94 (C), 150.63 (C), 135.98 (C), 135.19 (C), 130.34 (CH), 129.54 (CH), 129.50 (CH), 128.40 (CH), 125.04 (CH), 123.00 (CH), 118.01 (CH), 116.32 (C), 113.75 (C), 69.53 (CH), 52.50 (C), 49.54 (CH_2_), 28.85 (CH_3_); MS (qTOF) *m*/*z* (%) 406 (M^+^ + 1, 60), 321 (27), 279 (100); HRMS (qTOF) Calcd for C_23_H_24_N_3_O_4_: 406.1767. Found: 406.1761.

*2-(3,5-Dioxo-3,4-dihydro-2H-chromeno[3,4-b]pyrazin-1(5H)-yl)-N-pentyl-2-phenylacetamide* (**20p**). Obtained from **17p** as a white solid (92 mg, 55%); m.p. 206–208 °C (dec.); IR (cm^−1^) 3310, 3276, 2929, 1687, 1616, 1567, 1496, 1471, 1380, 1115, 999, 931, 748, 726; ^1^H-NMR (500 MHz, CDCl_3_) δ 7.63 (dd, *J* = 8.0, 1.2 Hz, 1H), 7.51 (dt, 6.9, *J* = 1.3 Hz, 2H), 7.43–7.37 (m, 4H), 7.35–7.28 (m, 4H), 6.04 (t, *J* = 5.4 Hz, 1H), 5.39 (s, 1H), 3.92 (d, *J* = 1.7 Hz, 2H), 3.37 (dd, *J* = 13.2, 7.0 Hz, 2H), 1.57–1.50 (m, 2H), 1.37–1.25 (m, 4H), 0.90 (t, *J* = 7.1 Hz, 3H); ^13^C-NMR (126 MHz, CDCl_3_) δ 168.65 (C), 162.80 (C), 156.78 (C), 150.47 (C), 135.51 (C), 134.77 (C), 130.21 (CH), 129.44 (CH), 129.39 (CH), 128.38 (CH), 124.94 (CH), 122.83 (CH), 117.87 (CH), 116.16 (C), 114.07 (C), 69.04 (CH), 49.40 (CH_2_), 40.00 (CH_2_), 29.19 (CH_2_), 29.04 (CH_2_), 22.27 (CH_2_), 13.97 (CH_3_); MS (qTOF) *m*/*z* (%) 420 (M^+^ + 1, 14), 293 (100); HRMS (qTOF) Calcd for C_24_H_26_N_3_O_4_: 420.1923. Found: 420.1918.

*N-benzyl-2-(3,5-dioxo-3,4-dihydro-2H-chromeno[3,4-b]pyrazin-1(5H)-yl)-2-phenylacetamide* (**20q**). Obtained from **17q** as a white solid (118 mg, 67%); m.p. 238–240 °C (dec.); IR (cm^−1^) 3280, 1686, 1618, 1496, 1379, 1113, 751, 700; ^1^H-NMR (500 MHz, CDCl_3_) δ 7.59 (dd, *J* = 8.0, 1.1 Hz, 1H), 7.52–7.44 (m, 2H), 7.42–7.33 (m, 6H), 7.33–7.25 (m, 6H), 6.33 (t, *J* = 5.5 Hz, 1H), 5.42 (s, 1H), 4.56 (qd, *J* = 14.6, 5.8 Hz, 2H), 3.92 (*d*, J = 1.7 Hz, 2H); ^13^C-NMR (126 MHz, CDCl_3_) δ 168.90 (C), 162.97 (C), 156.87 (C), 150.57 (C), 137.66 (C), 135.55 (C), 134.61 (C), 130.39 (CH), 129.63 (CH), 129.52 (CH), 129.06 (CH), 128.55 (CH), 128.06 (CH), 128.01 (CH), 125.10 (CH), 122.89 (CH), 118.00 (CH), 116.18 (C), 114.29 (C), 69.11 (CH), 49.43 (CH_2_), 44.15 (CH_2_); MS (qTOF) *m*/*z* (%) 440 (M^+^ + 1, 100), 282 (40), 169 (15); HRMS (qTOF) Calcd for C_26_H_22_N_3_O_4_: 440.1610. Found: 440.1585.

*N-benzyl-2-(3,5-dioxo-3,4-dihydro-2H-chromeno[3,4-b]pyrazin-1(5H)-yl)-2-(p-tolyl)acetamide* (**20r**). Obtained from **17r** as a white solid (141 mg, 78%); m.p. 221–223 °C (dec.); IR (cm^−1^) 3369, 1711, 1665, 1649, 1559, 1495, 1113, 754, 703; ^1^H-NMR (500 MHz, CDCl_3_) δ 7.79 (bs, 1H), 7.58 (dt, *J* = 7.2, 1.2 Hz, 1H), 7.54 (dd, *J* = 8.1, 1.2 Hz, 1H), 7.51–7.54 (m, 5H), 7.42–7.38 (m, 5H), 7.36–7.23 (m, 2H), 6.38 (t, *J* = 6.4 Hz, 1H), 5.79 (s, 1H), 4.75 (dd, *J* = 14.6, 6.2 Hz, 1H), 4.57 (dd, *J* = 14.6, 5.5 Hz, 1H), 4.21 (d, *J* = 16.7 Hz, 1H), 4.09 (d, *J* = 16.7 Hz, 1H), 2.14 (s, 3H); ^13^C-NMR (126 MHz, CDCl_3_) δ 169.46 (C), 162.69 (C), 156.85 (C), 150.57 (C), 137.65 (C), 137.27 (C), 136.89 (C), 133.46 (C), 131.91 (CH), 130.43 (CH), 129.69 (CH), 129.06 (CH), 128.65 (CH), 128.12 (CH), 128.06 (CH), 127.18 (CH), 124.95 (CH), 123.05 (CH), 118.07 (CH), 116.26 (C), 112.10 (C), 66.93 (CH), 49.79 (CH_2_), 44.24 (CH_2_), 19.44 (CH_3_); MS (qTOF) *m*/*z* (%) 454 (M^+^ + 1, 27), 369 (73), 327 (100); HRMS (qTOF) Calcd for C_27_H_24_N_3_O_4_: 454.1767. Found: 454.1761.

#### 3.4.4. General Procedure for the Synthesis of Chromeno[3,4-b]piperazines 19**j**,**k**

Enol-Ugi adduct **17j**–**k** (0.4 mmol) and iron powder (9.6 mmol, 24 equiv) in glacial acetic acid (8 mL), were subjected to a procedure identical to the one used for the reduction of nitro derivatives **17a**–**s**, except that the reaction was performed at 150 °C. Chromeno[3,4-*b*]piperazines **19j**,**k** were obtained after flash column chromatography purification (SiO_2_, gradient from 100 % hexanes to hexanes–AcOEt, 7:3).

*1,2-Diphenyl-1,4-dihydro-2H-chromeno[3,4-b]pyrazine-3,5-dione* (**19j**). Obtained from **18j** as a pale yellow solid (103 mg, 70%); m.p. 243–245 °C; IR (cm^−1^) 3423, 3075, 1716, 1679, 1631, 1492, 1470, 1407, 1369, 1234, 1131, 998, 754, 593; ^1^H-NMR (500 MHz, CDCl_3_) δ 8.15 (bs, 1H), 7.51 (dd, *J* = 6.3, 1.7 Hz, 2H), 7.43–7.30 (m, 7H), 7.22 (t, *J* = 7.4 Hz, 1H), 7.15 (dd, *J* = 8.1, 1.2 Hz, 1H), 7.09 (d, *J* = 8.0 Hz, 2H), 7.06 (d, *J* = 7.3 Hz, 1H), 5.64 (s, 1H); ^13^C-NMR (126 MHz, CDCl_3_) δ 162.88 (C), 156.74 (C), 150.88 (C), 145.76 (C), 135.81 (C), 131.77 (C), 130.07 (CH), 130.00 (CH), 129.41 (CH), 128.86 (CH), 125.89 (CH), 125.63 (CH), 124.99 (CH), 124.40 (CH), 122.53 (CH), 117.80 (CH), 115.63 (C), 111.31 (C), 69.02 (CH); MS (qTOF) *m*/*z* (%) 369 (M^+^ + 1, 100), 288 (<5); HRMS (qTOF) Calcd for C_23_H_17_N_2_O_3_: 369.1239. Found: 369.1228.

*2-(3,4-Dimethoxyphenyl)-1-phenyl-1,4-dihydro-2H-chromeno[3,4-b]pyrazine-3,5-dione* (**19k**). Obtained from **18k** as a pale yellow solid (120 mg, 70%); m.p. 209–211 °C; IR (cm^−1^) 3423, 3072, 1712, 1678, 1632, 1604, 1514, 1403, 1260, 1140, 1024, 999, 757; ^1^H-NMR (500 MHz, CDCl_3_) δ 8.12 (bs, 1H), 7.44–7.31 (m, 4H), 7.22 (t, *J* = 7.4 Hz, 1H), 7.15 (dd, *J* = 8.1, 1.1 Hz, 1H), 7.12–7.04 (m, 4H), 6.98 (dd, *J* = 8.4, 2.1 Hz, 1H), 6.79 (d, *J* = 8.4 Hz, 1H), 5.59 (s, 1H), 3.83 (s, 3H), 3.72 (s, 3H); ^13^C-NMR (126 MHz, CDCl_3_) δ 163.24 (C), 156.70 (C), 150.79 (C), 149.64 (C), 149.40 (C), 145.48 (C), 131.68 (C), 130.10 (CH), 129.97 (CH), 127.65 (C), 125.76 (CH), 124.73 (CH), 124.35 (CH), 122.25 (CH), 117.86 (CH), 117.44 (CH), 115.53 (C), 111.63 (CH), 111.28 (C), 108.67 (CH), 68.31 (CH), 56.07 (CH_3_), 55.91 (CH_3_); MS (qTOF) *m*/*z* (%) 429 (M^+^ + 1, 75), 369 (100), 276 (45); HRMS (qTOF) Calcd for C_25_H_21_N_2_O_5_: 429.1450. Found: 429.1454.

## 4. Conclusions

Multicomponent functionalization of 4-hydroxy-3-nitro-coumarin by an enol-Ugi condensation permits to introduce a peptidic chain that is subsequently cyclized in reducing conditions to build the fused piperazino ring. In this way, rigid polyheterocyclic di- and tri-peptides comprising a wide chemical diversity are easily accessible. This strategy, involving the first reported post-condensation transformation of an enol-Ugi adduct, opens new opportunities for the discovery of novel pharmacologically active compounds.

## Figures and Tables

**Table 1:** Synthesis of enol-Ugi adducts **17** and chromenopyrazines **19** ^a,b,d^.

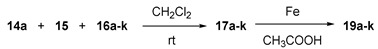
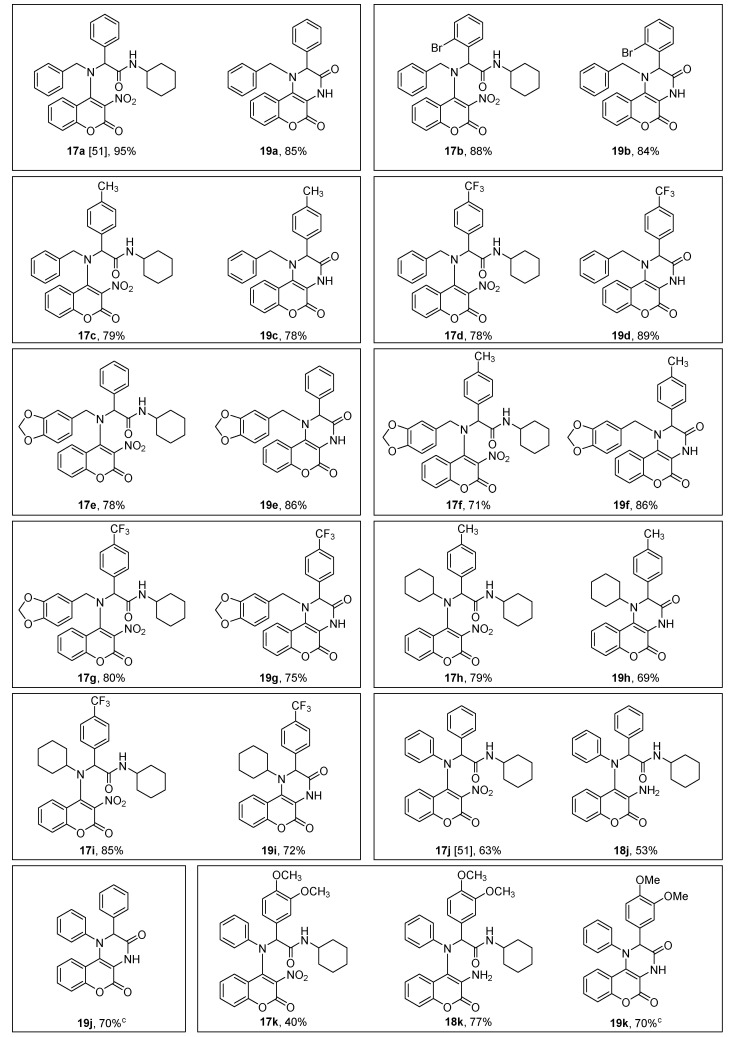

^a^ Procedure for the synthesis of **17a**–**k**: Isocyanide **14** (1 equiv) and enol **15** (1 equiv) were added to a solution of imine **16** (1 equiv) in CH_2_Cl_2_ and stirred at 20 °C for 3 h. ^b^ Procedure for the synthesis of **19a**–**i** and **18j**,**k**: Enol-Ugi adduct **17** (1 equiv) and iron powder (24 equiv) were stirred at rt for 2–4 h. ^c^ The reaction was performed at 150 °C. ^d^ All yields refer to isolated yields.

**Table 2:** Synthesis of enol-Ugi adducts of amino acids (**17**) and chromenopyrazines **19** and **20** ^a,b,c^.

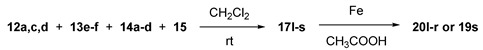
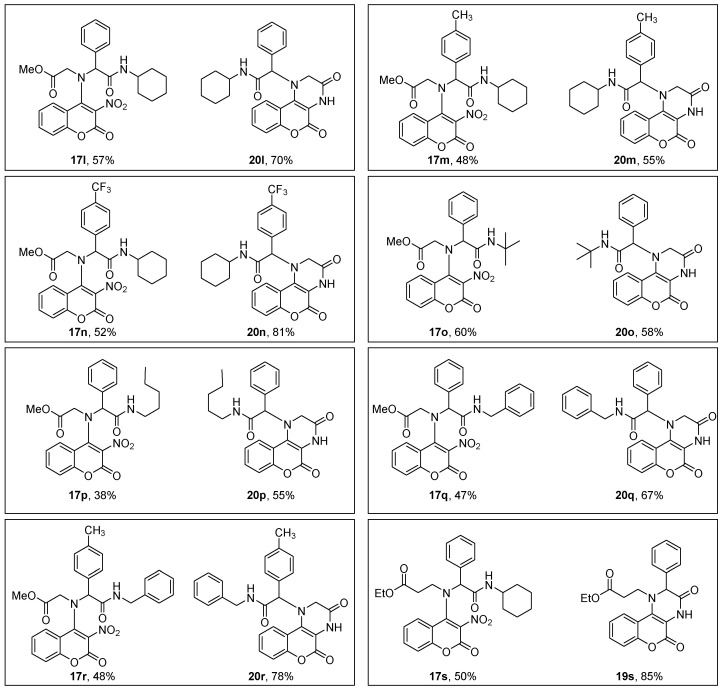

^a^ Procedure for the synthesis of **17l**–**s**: A solution of amine **13** (1 equiv) and aldehyde **12** (1 equiv) in of dry acetonitrile was stirred for 15 min at rt; isocyanide **14** (1 equiv) and enol **15** (1 equiv) were successively added and the reaction mixture was stirred 4 days at rt. ^b^ Procedure for the synthesis of **19s** and **20l**–**r**: Enol-Ugi adduct **17** (1 equiv) and iron powder (24 equiv) were stirred at rt in acetic acid for 2–4 h. ^c^ All yields refer to isolated yields.

## Data Availability

The data presented in this study is available in this article and in the supplementary material.

## Supplementary Materials

The following are available online: Figure S1: Aldehydes, amines, and isocyanides used as starting materials, Figure S2: Imines used as starting materials, Experimental data for imine **16g**, Copies of the NMR spectra for all new compounds.

## Sample Availability

Samples of the compounds are available from the authors.
