# Supplementary material for: Synthesis of Chromeno[3,4-b]piperazines by an Enol-Ugi/Reduction/Cyclization Sequence
Source: Molecules. 2021 Feb 27;26(5):1287. doi: 10.3390/molecules26051287 (PMC7956738; doi:10.3390/molecules26051287)

Supporting Information for

Synthesis of chromeno[3,4-*b*]piperazines by an enol-Ugi/reduction/cyclization sequence

Ana Bornadiego, Ana G. Neo* and Carlos F. Marcos.*

Laboratory of Bioorganic Chemistry & Membrane Biophysics (L.O.B.O.). School of Veterinary Sciences. University of Extremadura. 10003 Cáceres, Spain. E-mail: cfernan@unex.es, aneo@unex.es

CONTENT

Starting materials 3

NMR Spectra.. 6

(*E*)-*N*-(Benzo[*d*][1,3]dioxol-5-ylmethyl)-1-(4-(trifluoromethyl)phenyl)methanimine (16g). 6
2-(Benzyl(3-nitro-2-oxo-2*H*-chromen-4-yl)amino)-2-(2-bromophenyl)-*N*-cyclohexyl acetamide (17b). 7
2-(Benzyl(3-nitro-2-oxo-2*H*-chromen-4-yl)amino)-2-(2-bromophenyl)-*N*-cyclohexyl acetamide (17c). 8
2-(Benzyl(3-nitro-2-oxo-2*H*-chromen-4-yl)amino)-*N*-cyclohexyl-2-(4-(trifluoromethyl) phenyl)acetamide (17d)... 9
2-((Benzo[*d*][1,3]dioxol-5-ylmethyl)(3-nitro-2-oxo-2*H*-chromen-4-yl)amino)-*N*-cyclohexyl-2-phenylacetamide (17e). 10
2-((Benzo[*d*][1,3]dioxol-5-ylmethyl)(3-nitro-2-oxo-2*H*-chromen-4-yl)amino)-*N*-cyclohexyl-2-(*p*-tolyl)acetamide (17f). 11
2-((Benzo[*d*][1,3]dioxol-5-ylmethyl)(3-nitro-2-oxo-2*H*-chromen-4-yl)amino)-*N*-cyclohexyl-2-(4-(trifluoromethyl)phenyl)acetamide (17g). 12
N-Cyclohexyl-2-(cyclohexyl(3-nitro-2-oxo-2*H*-chromen-4-yl)amino)-2-(*p*-tolyl)acetamide (17h). 13
N-Cyclohexyl-2-(cyclohexyl(3-nitro-2-oxo-2*H*-chromen-4-yl)amino)-2-(4-(trifluoromethyl) phenyl)acetamide (17i)... 14
N-Cyclohexyl-2-(3,4-dimethoxyphenyl)-2-((3-nitro-2-oxo-2*H*-chromen-4-yl)(phenyl)amino) acetamide (17k)... 15
Methyl *N*-(2-(cyclohexylamino)-2-oxo-1-phenylethyl)-*N*-(3-nitro-2-oxo-2*H*-chromen-4-yl)glycinate (17l)... 16
Methyl *N*-(2-(cyclohexylamino)-2-oxo-1-(*p*-tolyl)ethyl)-*N*-(3-nitro-2-oxo-2*H*-chromen-4-yl)glycinate (17m)... 17
Methyl *N*-(2-(cyclohexylamino)-2-oxo-1-(4-(trifluoromethyl)phenyl)ethyl)-*N*-(3-nitro-2-oxo-2*H*-chromen-4-yl)glycinate (17n). 18
Methyl *N*-(2-(*tert*-butylamino)-2-oxo-1-phenylethyl)-*N*-(3-nitro-2-oxo-2H-chromen-4-yl)glycinate (17o). 19
Methyl *N*-(3-nitro-2-oxo-2*H*-chromen-4-yl)-*N*-(2-oxo-2-(pentylamino)-1-phenylethyl) glycinate (17g). 20
Methyl *N*-(2-(benzylamino)-2-oxo-1-phenylethyl)-*N*-(3-nitro-2-oxo-2*H*-chromen-4-yl)glycinate (17q) 21
Methyl *N*-(2-(benzylamino)-2-oxo-1-(*p*-tolyl)ethyl)-*N*-(3-nitro-2-oxo-2*H*-chromen-4-yl)glycinate (17r)... 22
Ethyl 3-((2-(cyclohexylamino)-2-oxo-1-phenylethyl)(3-nitro-2-oxo-2*H*-chromen-4-yl)amino)propanoate (17s). 23
1-Benzyl-2-phenyl-1,4-dihydro-2*H*-chromeno[3,4-*b*]pyrazine-3,5-dione (19a)........................ 24

1-Benzyl-2-(2-bromophenyl)-1,4-dihydro-2*H*-chromeno[3,4-*b*]pyrazine-3,5-dione (19b) 25
1-Benzyl-2-(*p*-tolyl)-1,4-dihydro-2*H*-chromeno[3,4-*b*]pyrazine-3,5-dione (19c). 26
1-Benzyl-2-(4-(trifluoromethyl)phenyl)-1,4-dihydro-2*H*-chromeno[3,4-*b*]pyrazine-3,5-dione (19d). 27
1-(Benzo[*d*][1,3]dioxol-5-ylmethyl)-2-phenyl-1,4-dihydro-2*H*-chromeno[3,4-*b*]pyrazine-3,5-dione (19e). 28
1-(Benzo[*d*][1,3]dioxol-5-ylmethyl)-2-(*p*-tolyl)-1,4-dihydro-2*H*-chromeno[3,4-*b*]pyrazine-3,5-dione (19f). 29
1-(Benzo[*d*][1,3]dioxol-5-ylmethyl)-2-(4-(trifluoromethyl)phenyl)-1,4-dihydro-2*H*-chromeno[3,4-*b*]pyrazine-3,5-dione (19g). 30
1-Cyclohexyl-2-(*p*-tolyl)-1,4-dihydro-2*H*-chromeno[3,4-*b*]pyrazine-3,5-dione (19h). 31
1-Cyclohexyl-2-(4-(trifluoromethyl)phenyl)-1,4-dihydro-2*H*-chromeno[3,4-*b*]pyrazine-3,5-dione (19i) 32
2-((3-Amino-2-oxo-2*H*-chromen-4-yl)(phenyl)amino)-*N*-cyclohexyl-2-phenylacetamide (18j). 33
2-((3-Amino-2-oxo-2*H*-chromen-4-yl)(phenyl)amino)-*N*-cyclohexyl-2-(3,4-dimethoxyphenyl)acetamide (18k). 34
1,2-Diphenyl-1,4-dihydro-2*H*-chromeno[3,4-*b*]pyrazine-3,5-dione (19j). 35
2-(3,4-Dimethoxyphenyl)-1-phenyl-1,4-dihydro-2*H*-chromeno[3,4-*b*]pyrazine-3,5-dione (19k). 36
N-cyclohexyl-2-(3,5-dioxo-3,4-dihydro-2*H*-chromeno[3,4-*b*]pyrazin-1(5*H*)-yl)-2-phenyl acetamide (20l) 37
N-cyclohexyl-2-(3,5-dioxo-3,4-dihydro-2*H*-chromeno[3,4-*b*]pyrazin-1(5*H*)-yl)-2-(*p*-tolyl)acetamide (20m). 38
N-cyclohexyl-2-(3,5-dioxo-3,4-dihydro-2*H*-chromeno[3,4-*b*]pyrazin-1(5*H*)-yl)-2-(4-(trifluoromethyl)phenyl)acetamide (20n). 39
N-(*tert*-butyl)-2-(3,5-dioxo-3,4-dihydro-2*H*-chromeno[3,4-*b*]pyrazin-1(5*H*)-yl)-2-phenylacetamide (20o). 40
2-(3,5-Dioxo-3,4-dihydro-2*H*-chromeno[3,4-*b*]pyrazin-1(5*H*)-yl)-*N*-pentyl-2-phenylacetamide (20p). 41
N-benzyl-2-(3,5-dioxo-3,4-dihydro-2*H*-chromeno[3,4-*b*]pyrazin-1(5*H*)-yl)-2-phenylacetamide (20q). 42
N-benzyl-2-(3,5-dioxo-3,4-dihydro-2*H*-chromeno[3,4-*b*]pyrazin-1(5*H*)-yl)-2-(*p*-tolyl)acetamide (20r). 43
Ethyl 3-(3,5-dioxo-2-phenyl-3,4-dihydro-2*H*-chromeno[3,4-*b*]pyrazin-1(5*H*)-yl)propanoate (19s). 44

Starting materials

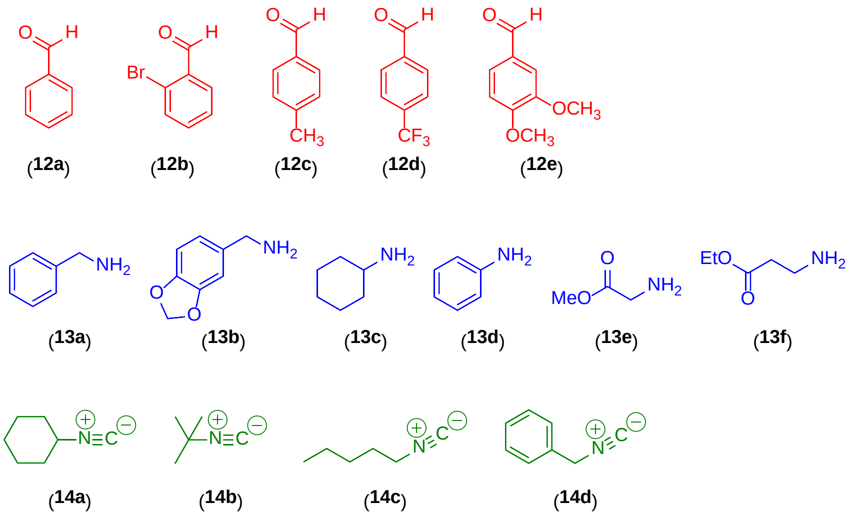

Figure S1. Aldehydes, amines, and isocyanides used as starting materials.

Synthesis and characterization of imines

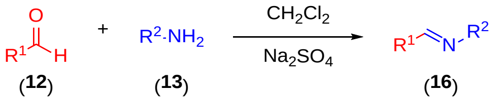

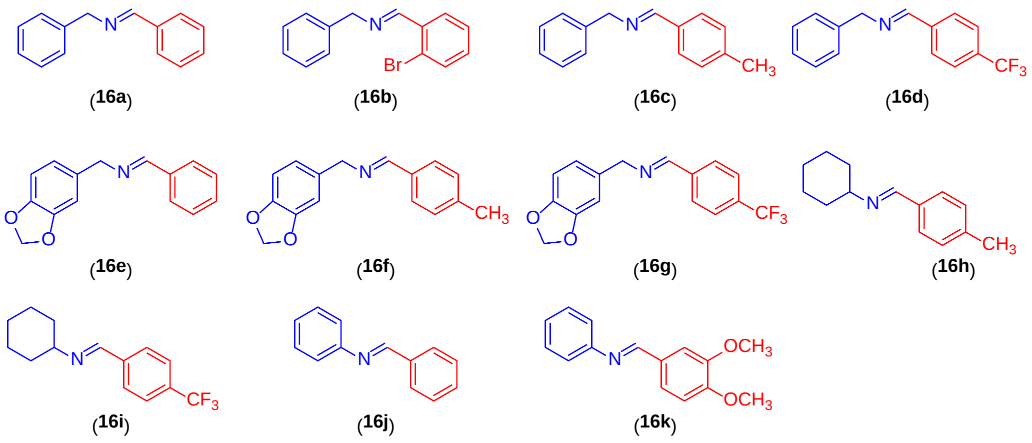

Figure S2. Imines used as starting materials.

General procedure: To a solution of 10 mmol of aldehyde **12** in 20 mL of dry CH_2Cl_2, 10 mmol of anhydrous Na_2SO_4 and 10 mmol of amine **13** were successively added. After 24 hours stirring at room temperature, the reaction went to completion, as judged by tlc. Then the Na_2SO_4

was filtered off and the solvent was eliminated to yield the imines **16** that were used without further purification in the Ugi reaction.

(*E*)-*N*-benzyl-1-phenylmethanimine (**16a**) [1], (*E*)-*N*-benzyl-1-(2-bromophenyl)methanimine (**16b**) [1], (*E*)-*N*-benzyl-1-(*p*-tolyl)methanimine (**16c**) [1], (*E*)-*N*-benzyl-1-(4-(trifluoromethyl)phenyl)methanimine (**16d**) [1], (*E*)-*N*-(benzo[*d*][1,3]dioxol-5-ylmethyl)-1-phenylmethanimine (**16e**) [2], (*E*)-*N*-(benzo[*d*][1,3]dioxol-5-ylmethyl)-1-(*p*-tolyl)methanimine (**16f**) [3], (*E*)-*N*-cyclohexyl-1-(*p*-tolyl)methanimine (**16h**) [4], (*E*)-*N*-cyclohexyl-1-(4-(trifluoromethyl)phenyl)methanimine (**16i**) [5], (*E*)-*N*,1-diphenylmethanimine (**16j**) [6, 7] and (*E*)-1-(3,4-dimethoxyphenyl)-*N*-phenylmethanimine (**16k**) [8] showed physical and spectroscopic data consistent with the literature.

(*E*)-*N*-(Benzo[*d*][1,3]dioxol-5-ylmethyl)-1-(4-(trifluoromethyl)phenyl)methanimine (16g). Obtained from aldehyde **12d** and amine **13b** as a light brown oil (93%); IR (cm^{-1}) 2915, 2857, 2820, 1649, 1493, 1442, 1337,1247, 1106, 1066, 1045, 953, 931, 838, 801, 598; ^{1}H NMR (500 MHz, $CDCl_3$) δ 8.39 (s, 1H), 7.87 (d, J = 8.1 Hz, 2H), 7.66 (d, J = 8.2 Hz, 2H), 6.83 (s, 1H), 6.79 (d, J = 0.9 Hz, 2H), 5.94 (s, 2H), 4.75 (d, J = 0.9 Hz, 2H); ^{13}C NMR (126 MHz, $CDCl_3$) δ 160.29 (CH), 148.02 (C), 146.90 (C), 139.40 (C), 132.76 (C), 128.60 (CH), 125.75 (CH), 125.72 (CH), 125.69 (CH), 125.66 (CH), 121.32 (CH), 108.81 (CH), 108.45 (CH), 101.12 (CH_2), 64.93 (CH_2); MS (qTOF) m/z (%) 308 (M^+ + 1, 100), 135 (9); HRMS (qTOF) Calcd for $C_{16}H_{13}F_3NO_2$: 308.0898. Found: 308.094.

References

1. Seayad, A. M.; Ramalingam, B.; Yoshinaga, K.; Nagata, T.; Chai, C. L. L., Highly Enantioselective Titanium-Catalyzed Cyanation of Imines at Room Temperature. *Org. Lett.* **2010,** 12, (2), 264-267. doi: http://dx.doi.org/10.1021/ol902540h

2. Manju, B.; Kumar, V. P.; Neeraj, K.; Upendra, S.; Bikram, S., Highly efficient iron phthalocyanine catalyzed oxidative synthesis of imines from alcohols and amines. *Can. J. Chem.* **2013,** 91, (8), 732-737. doi: http://dx.doi.org/10.1139/cjc-2012-0399

3. Genc Bilgicli, H.; Taslimi, P.; Akyuz, B.; Tuzun, B.; Gulcin, I., Synthesis, characterization, biological evaluation, and molecular docking studies of some piperonyl-based 4-thiazolidinone derivatives. *Arch Pharm (Weinheim)* **2020,** 353, (1), e1900304. doi: http://dx.doi.org/10.1002/ardp.201900304

4. Anders, E.; Tropsch, J. G.; Katritzky, A. R.; Rasala, D.; Vanden Eynde, J. J., N-(1-haloalkyl)pyridinium salts: preparation and use for new syntheses of other N-(1-substituted-alkyl)pyridinium salts, N,N'-(1-alkylidene)bisamines, and N,N'-(1-alkylidene)bisbenzazoles. *J. Org. Chem.* **1989,** 54, (20), 4808-4812. doi: http://dx.doi.org/10.1021/jo00281a021

5. Elliott, D. C.; Marti, A.; Mauleon, P.; Pfaltz, A., H2 Activation by Non-Transition-Metal Systems: Hydrogenation of Aldimines and Ketimines with LiN(SiMe3)2. *Chemistry* **2019,** 25, (8), 1918-1922. doi: http://dx.doi.org/10.1002/chem.201805549

6. Nongkunsarn, P.; Ramsden, C. A., Oxidative rearrangement of imines to formamides using sodium perborate. *Tetrahedron* **1997,** 53, (10), 3805-3830. doi: http://dx.doi.org/https://doi.org/10.1016/S0040-4020(97)00101-4

7. Garcia Ruano, J. L.; Aleman, J.; Alonso, I.; Parra, A.; Marcos, V.; Aguirre, J., Pi-pi stacking versus steric effects in stereoselectivity control: highly diastereoselective synthesis of syn-1,2-diarylpropylamines. *Chemistry* **2007,** 13, (21), 6179-95. doi: http://dx.doi.org/10.1002/chem.200601893

8. Hiroyuki, M.; Masataka, M.; Takeshi, I.; Shū, K., Aerobic Oxidation of Amines Catalyzed by Polymer-Incarcerated Au Nanoclusters: Effect of Cluster Size and Cooperative Functional Groups in the Polymer. *Bull. Chem. Soc. Jpn.* **2011,** 84, (6), 588-599. doi: http://dx.doi.org/10.1246/bcsj.20100300

NMR Spectra

(*E*)-*N*-(Benzo[*d*][1,3]dioxol-5-ylmethyl)-1-(4-(trifluoromethyl)phenyl)methanimine (16g).

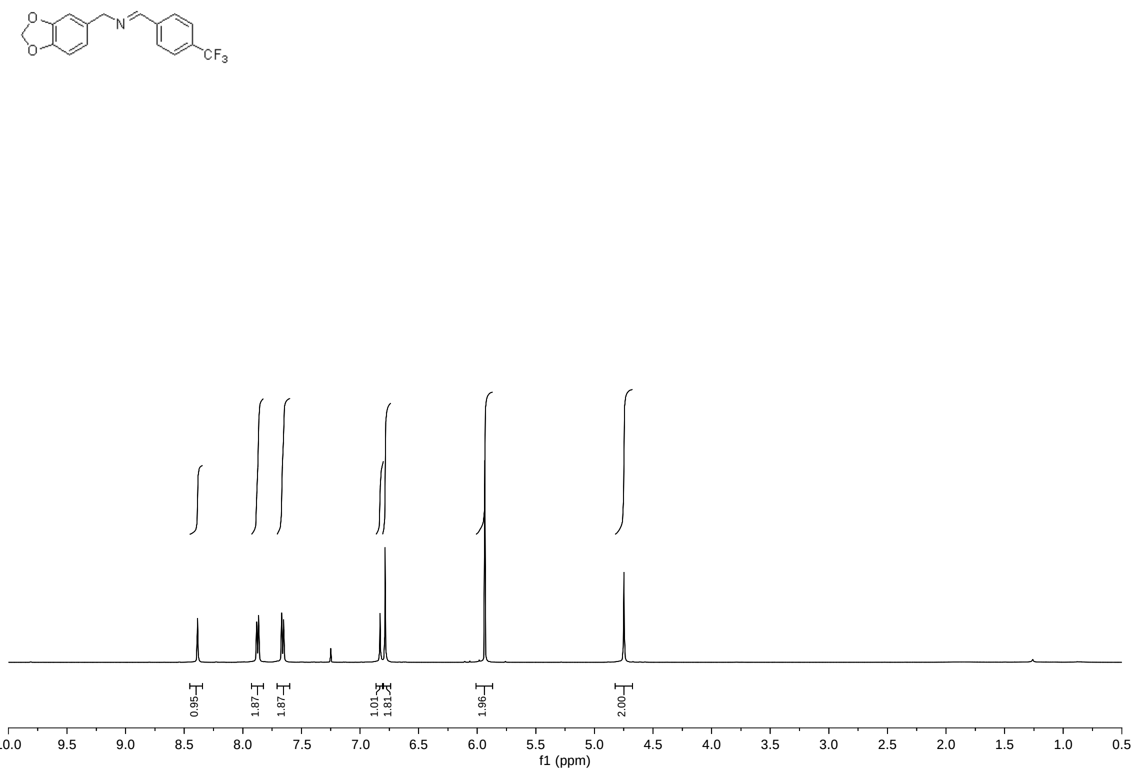

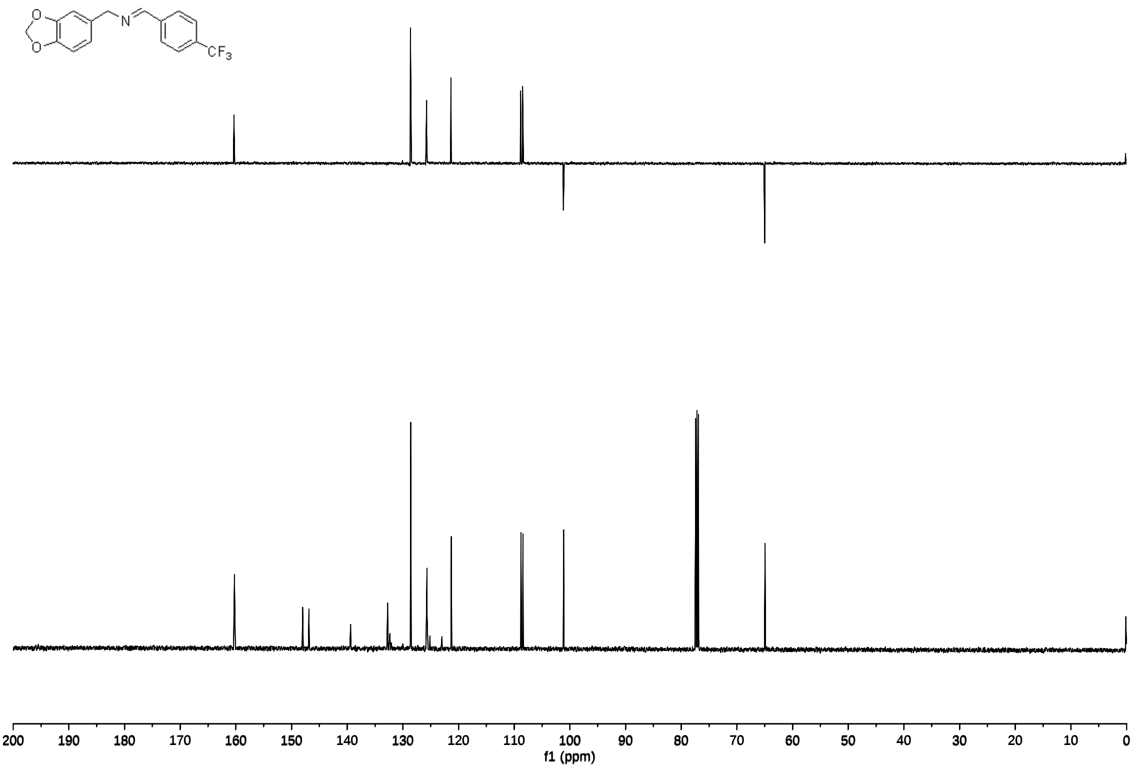

2-(Benzyl(3-nitro-2-oxo-2*H*-chromen-4-yl)amino)-2-(2-bromophenyl)-*N*-cyclohexyl acetamide (17b).

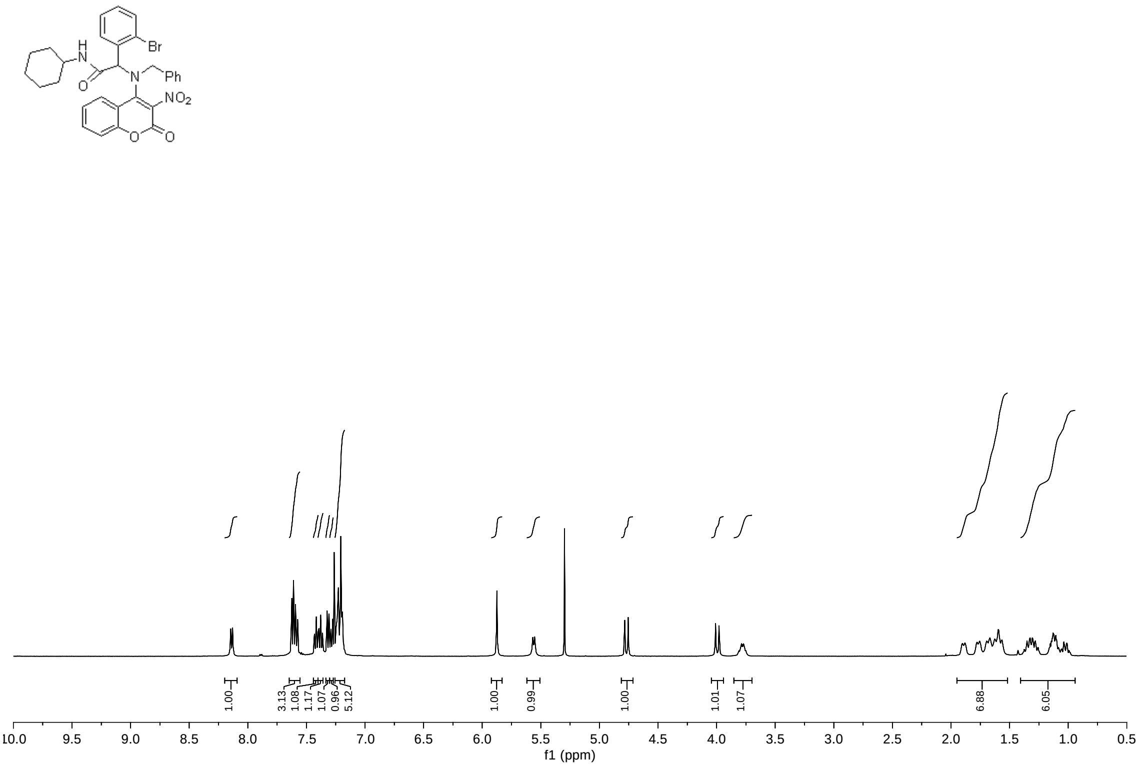

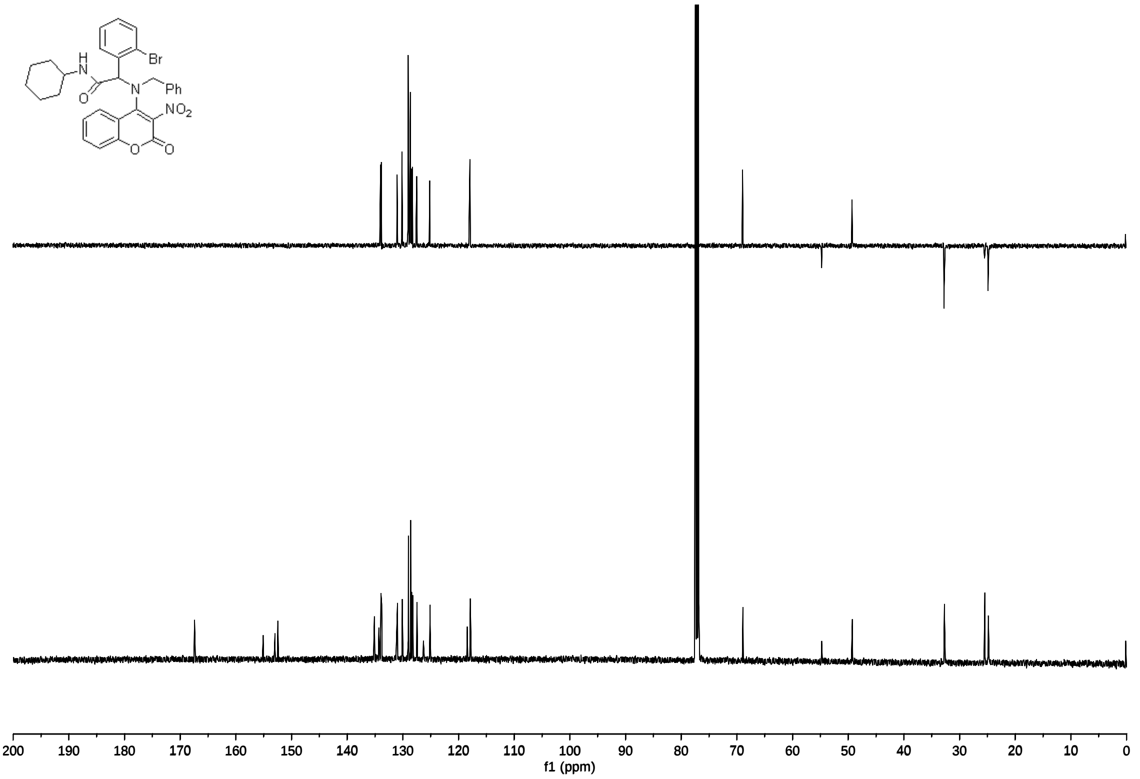

2-(Benzyl(3-nitro-2-oxo-2*H*-chromen-4-yl)amino)-2-(2-bromophenyl)-*N*-cyclohexyl acetamide (17c).

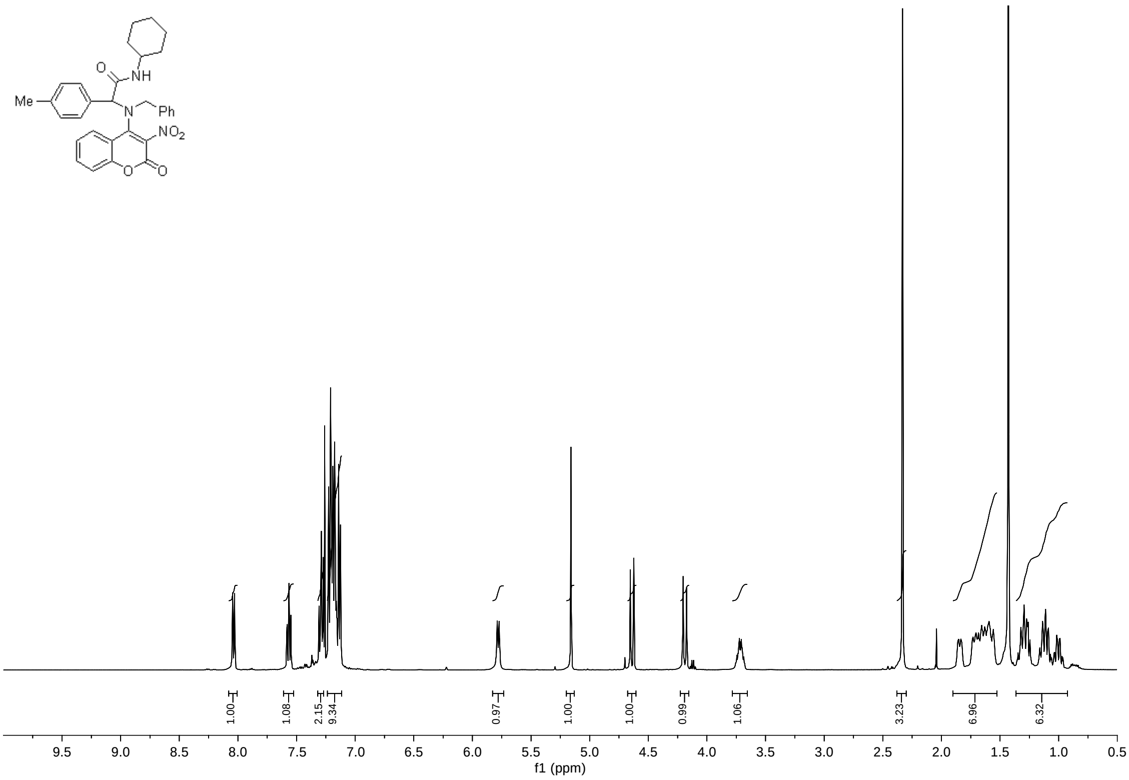

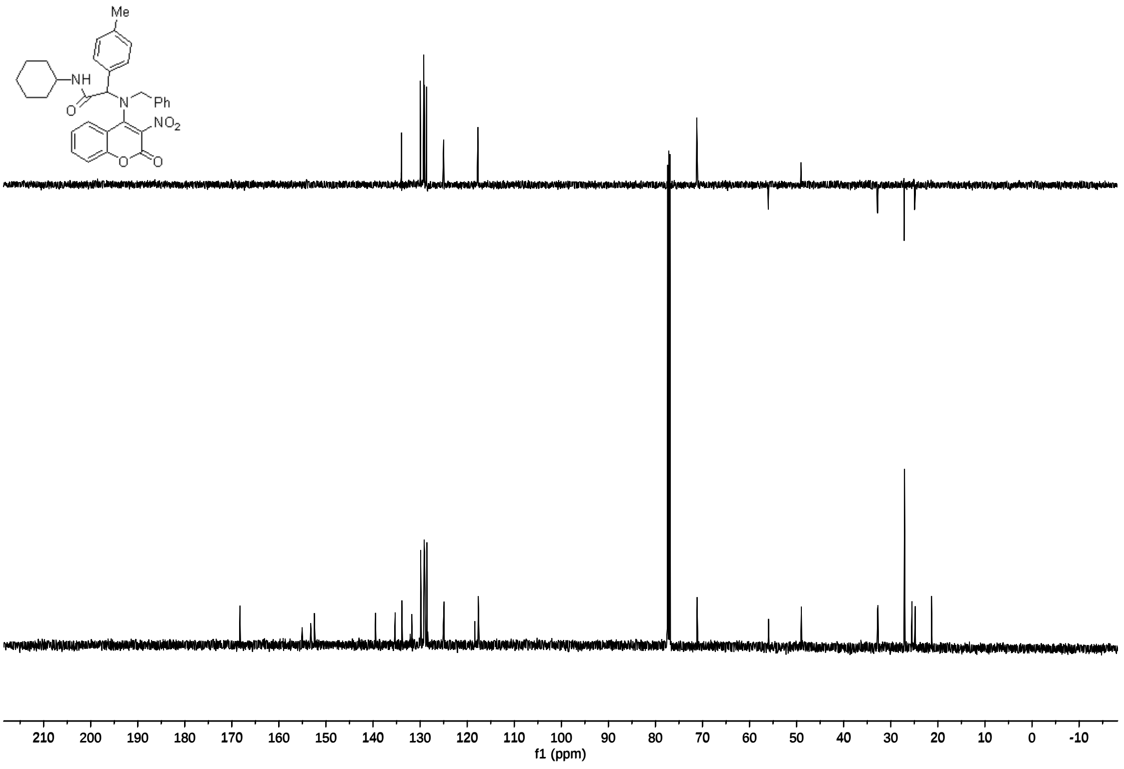

2-(Benzyl(3-nitro-2-oxo-2*H*-chromen-4-yl)amino)-*N*-cyclohexyl-2-(4-(trifluoromethyl) phenyl)acetamide (17d).

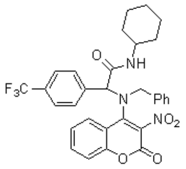

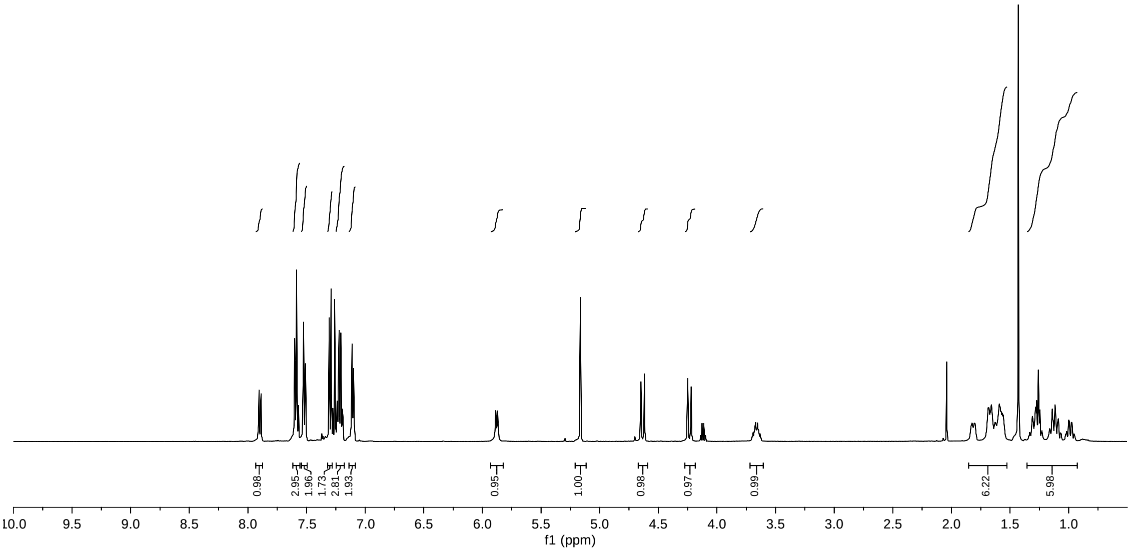

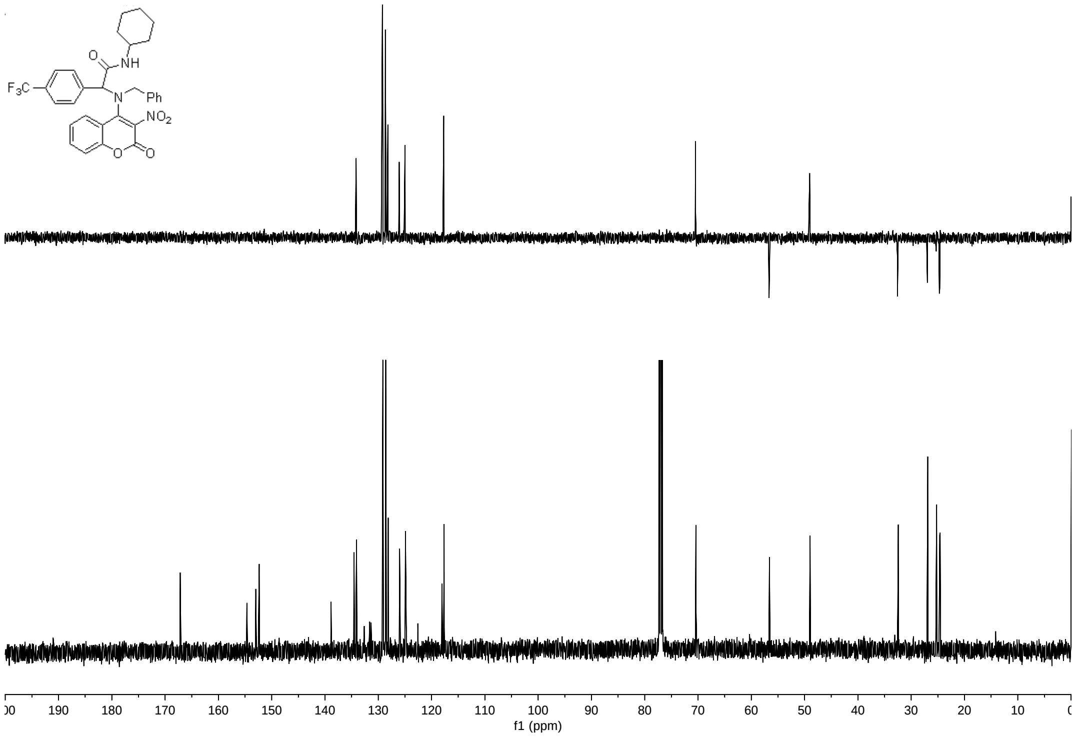

2-((Benzo[*d*][1,3]dioxol-5-ylmethyl)(3-nitro-2-oxo-2*H*-chromen-4-yl)amino)-*N*-cyclohexyl-2-phenylacetamide (17e).

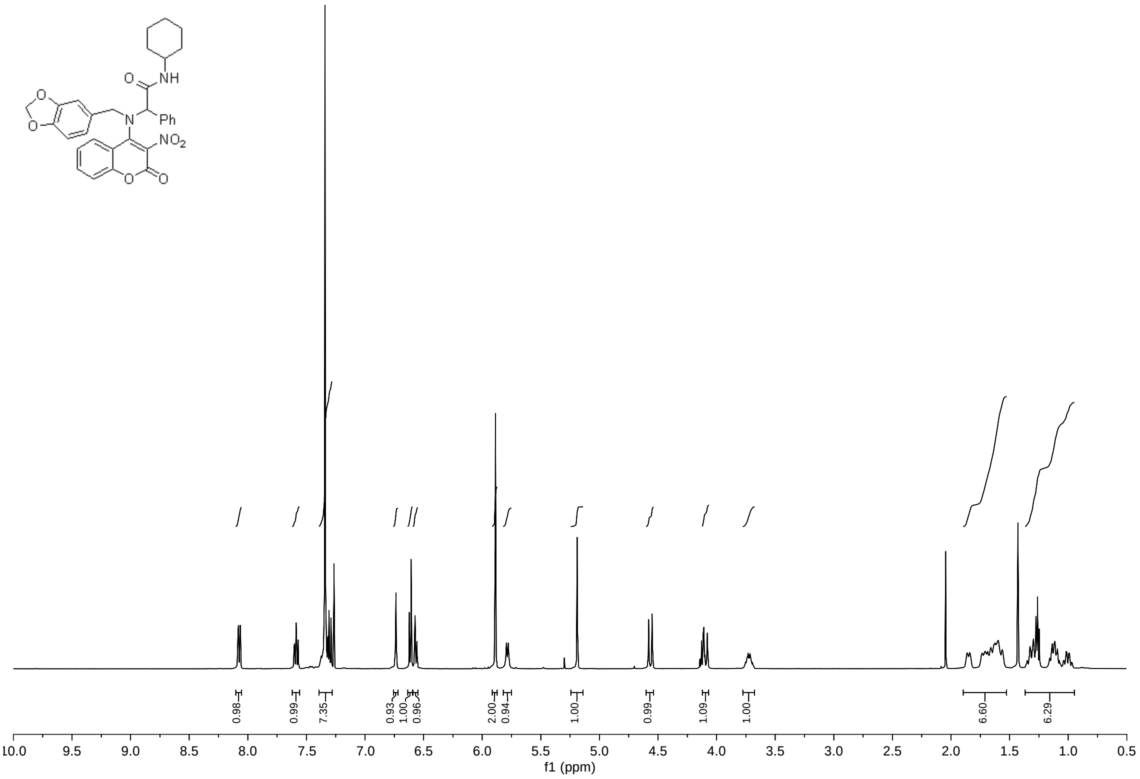

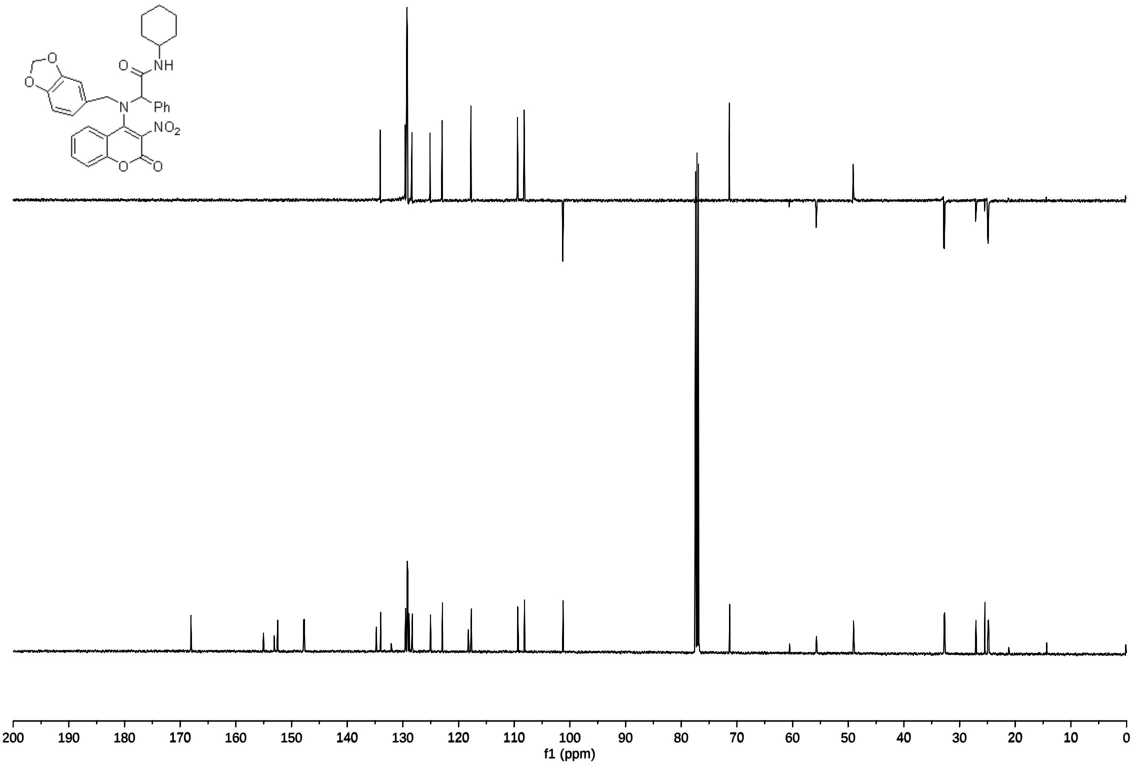

2-((Benzo[*d*][1,3]dioxol-5-ylmethyl)(3-nitro-2-oxo-2*H*-chromen-4-yl)amino)-*N*-cyclohexyl-2-(*p*-tolyl)acetamide (17f).

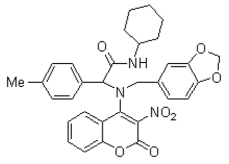

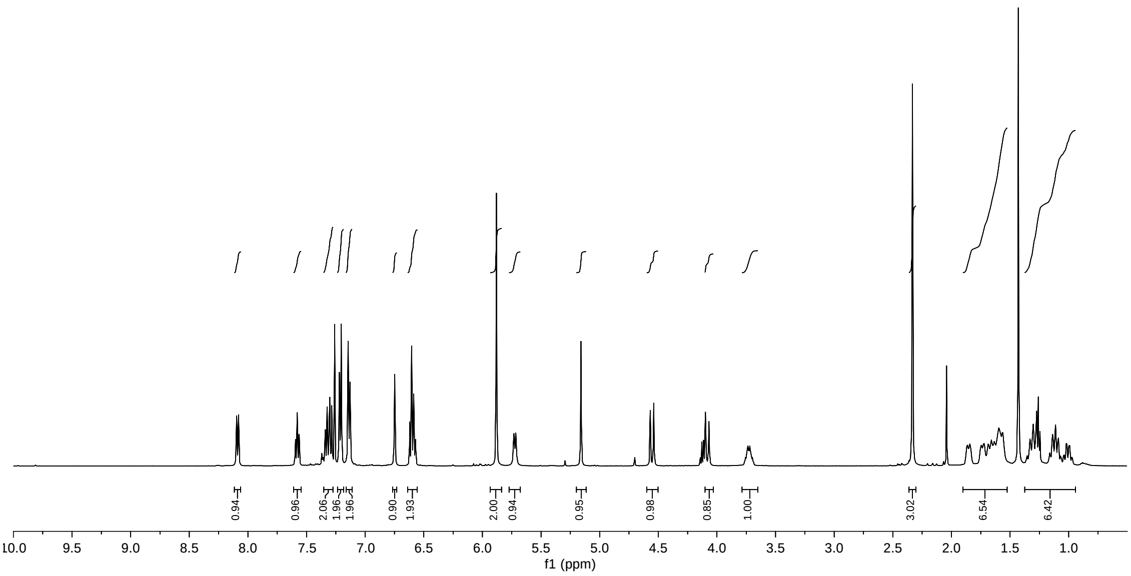

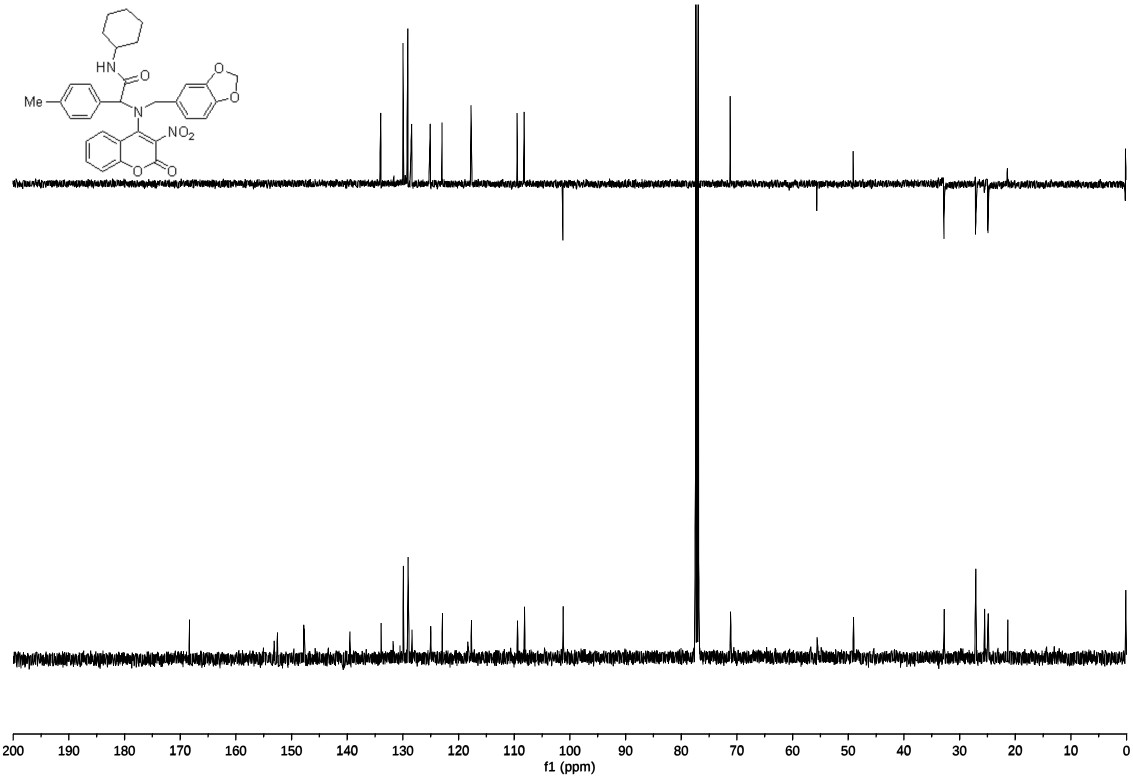

2-((Benzo[*d*][1,3]dioxol-5-ylmethyl)(3-nitro-2-oxo-2*H*-chromen-4-yl)amino)-*N*-cyclohexyl-2-(4-(trifluoromethyl)phenyl)acetamide (17g).

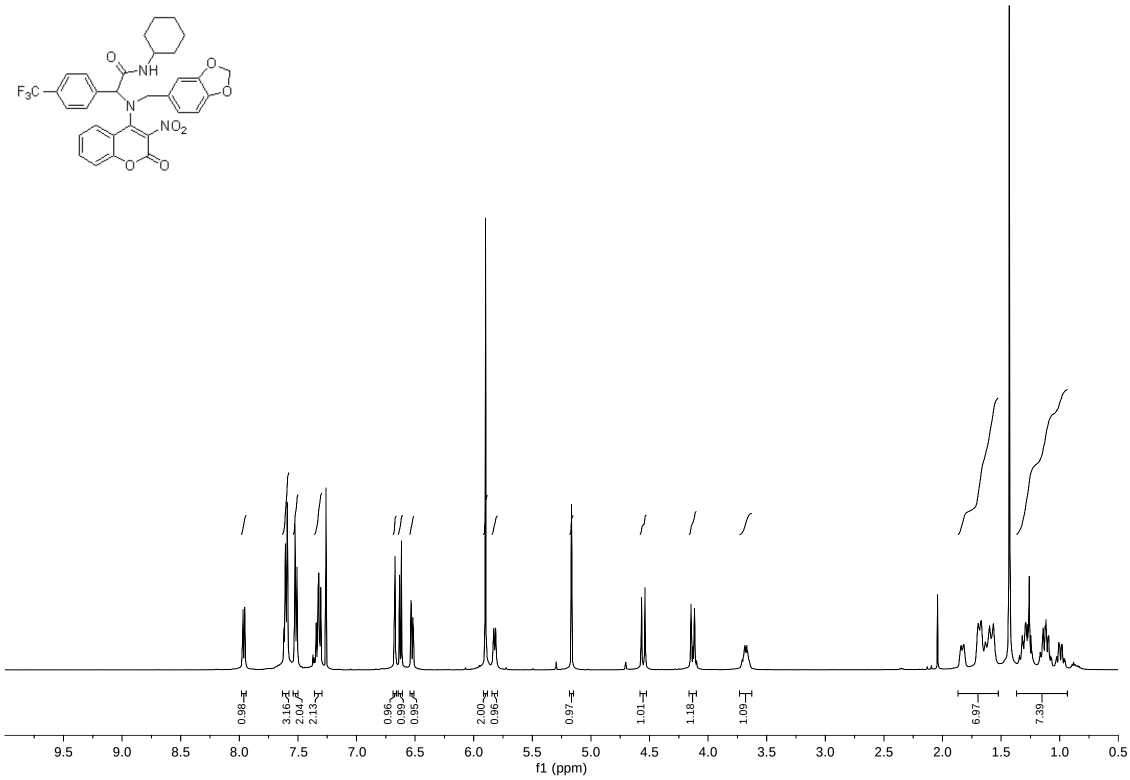

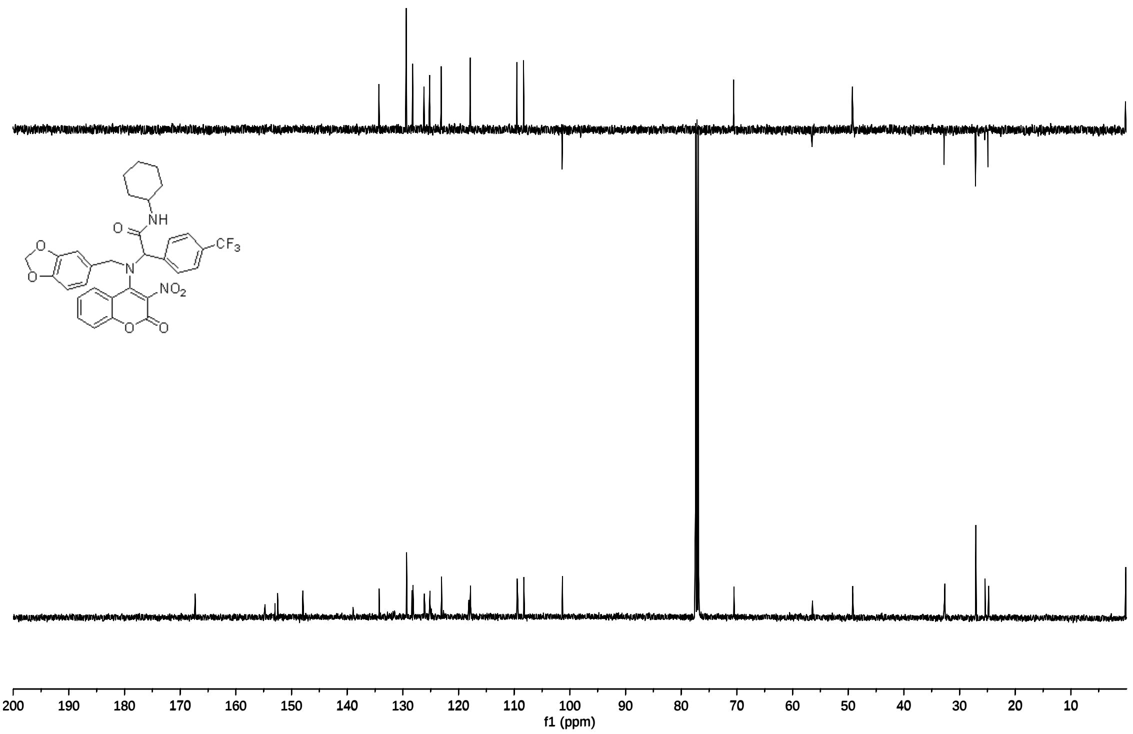

***N*-Cyclohexyl-2-(cyclohexyl(3-nitro-2-oxo-2*H*-chromen-4-yl)amino)-2-(*p*-tolyl)acetamide (17h).**

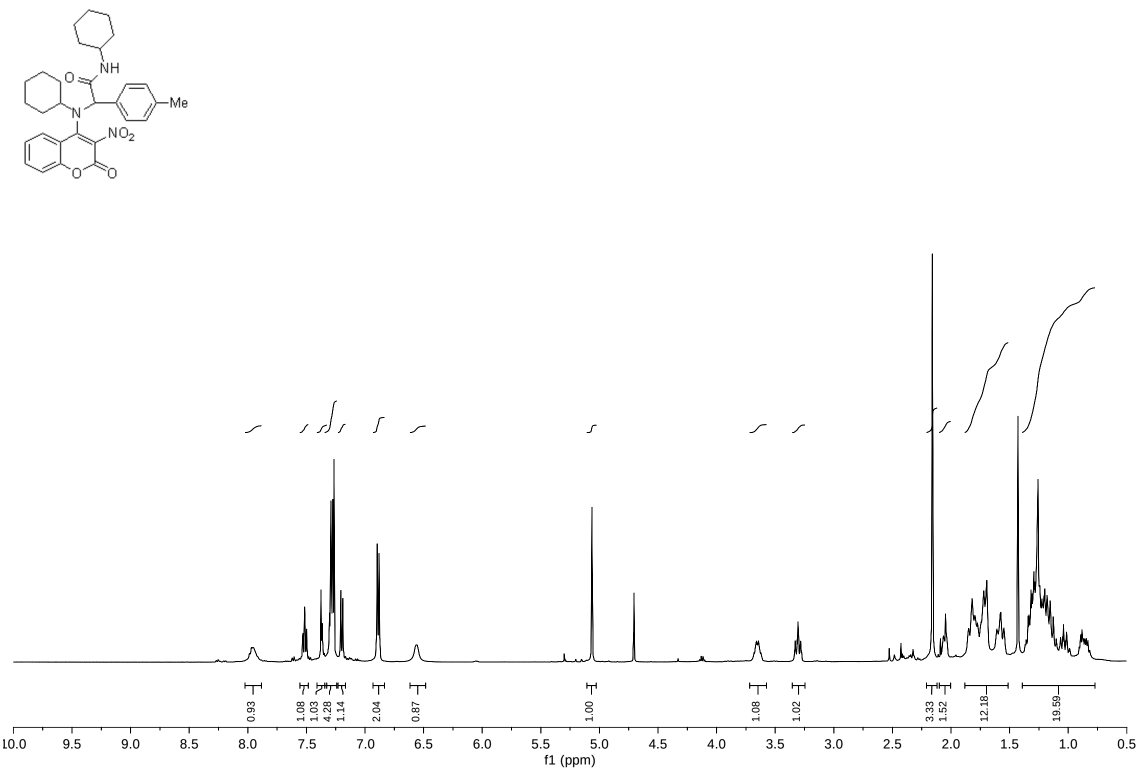

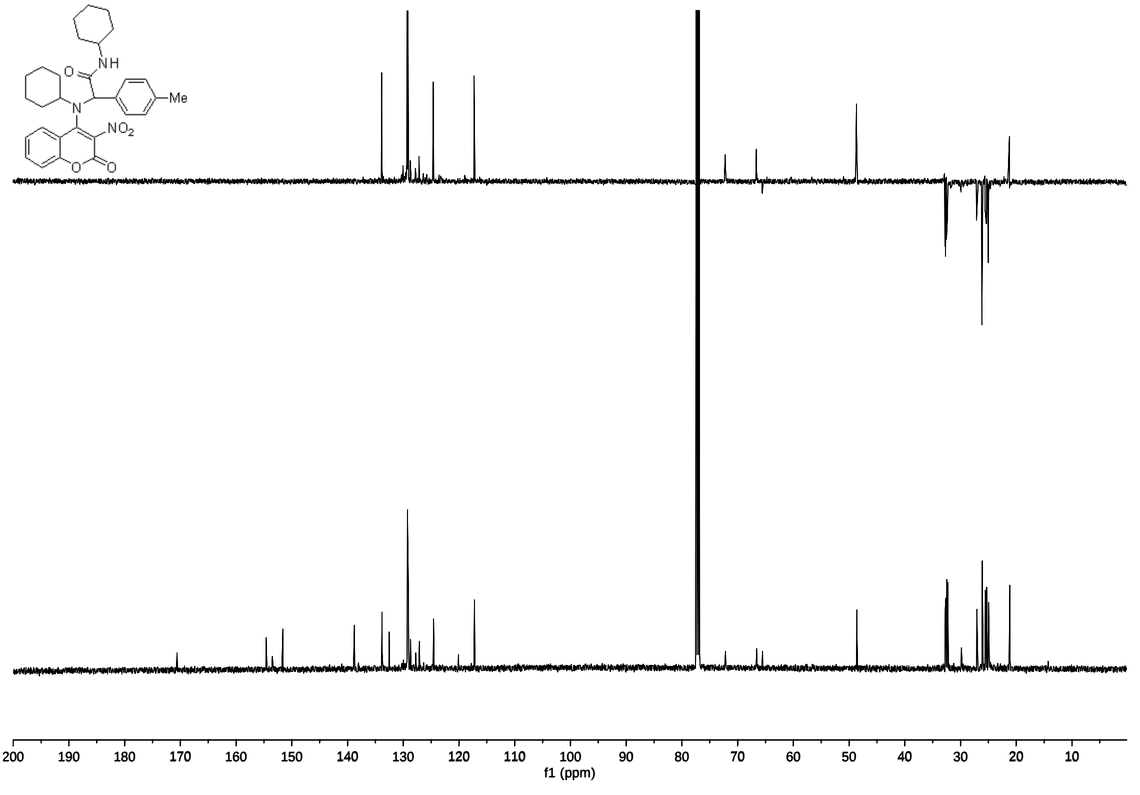

***N*-Cyclohexyl-2-(cyclohexyl(3-nitro-2-oxo-2*H*-chromen-4-yl)amino)-2-(4-(trifluoromethyl) phenyl)acetamide (17i).**

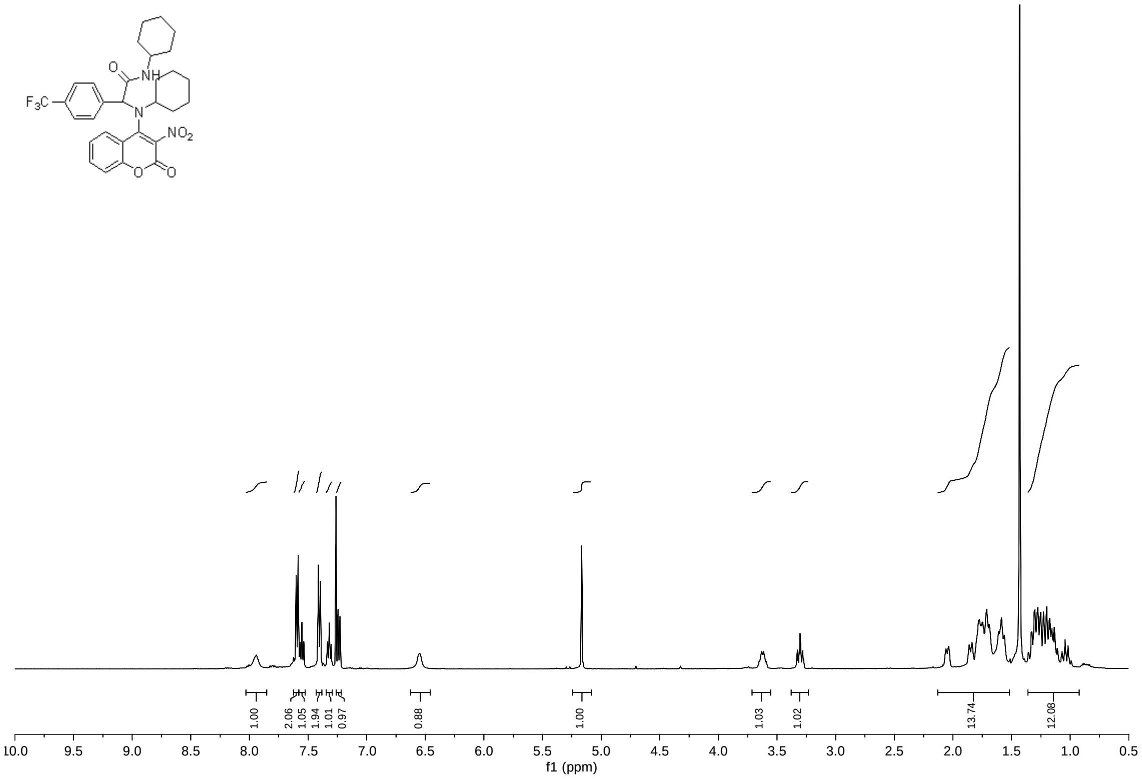

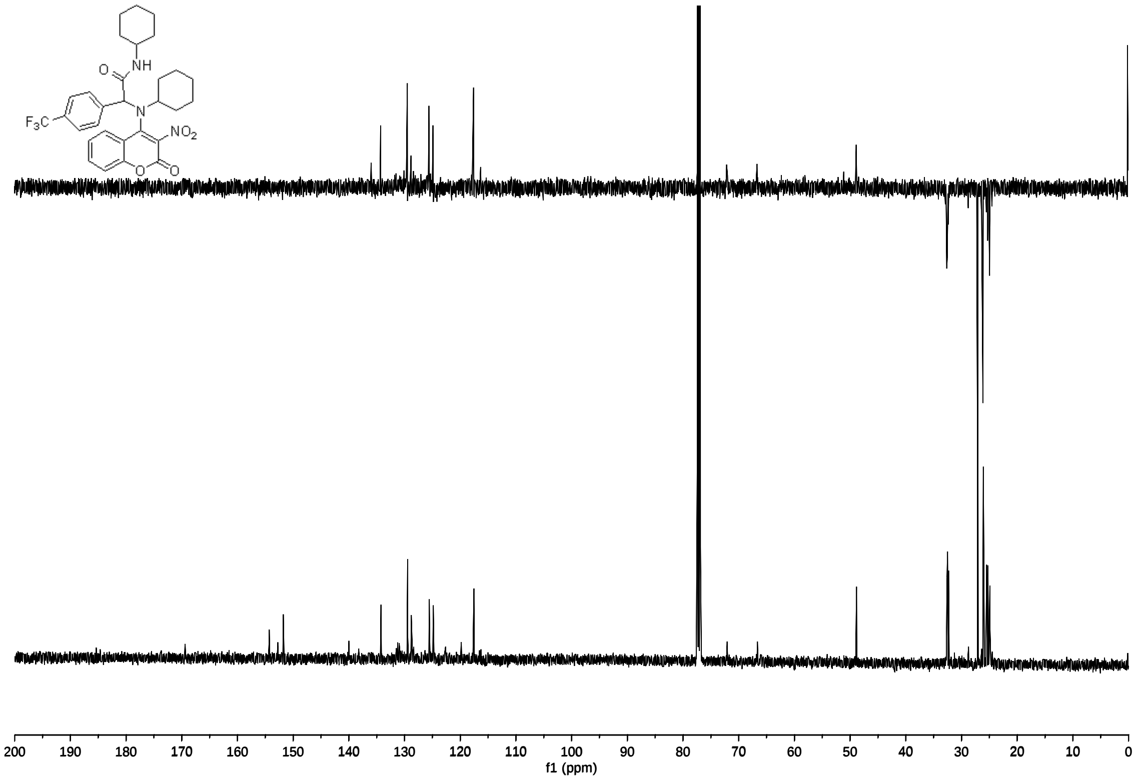

_N_-Cyclohexyl-2-(3,4-dimethoxyphenyl)-2-((3-nitro-2-oxo-2_H_-chromen-4-yl)(phenyl)amino) acetamide (17k).

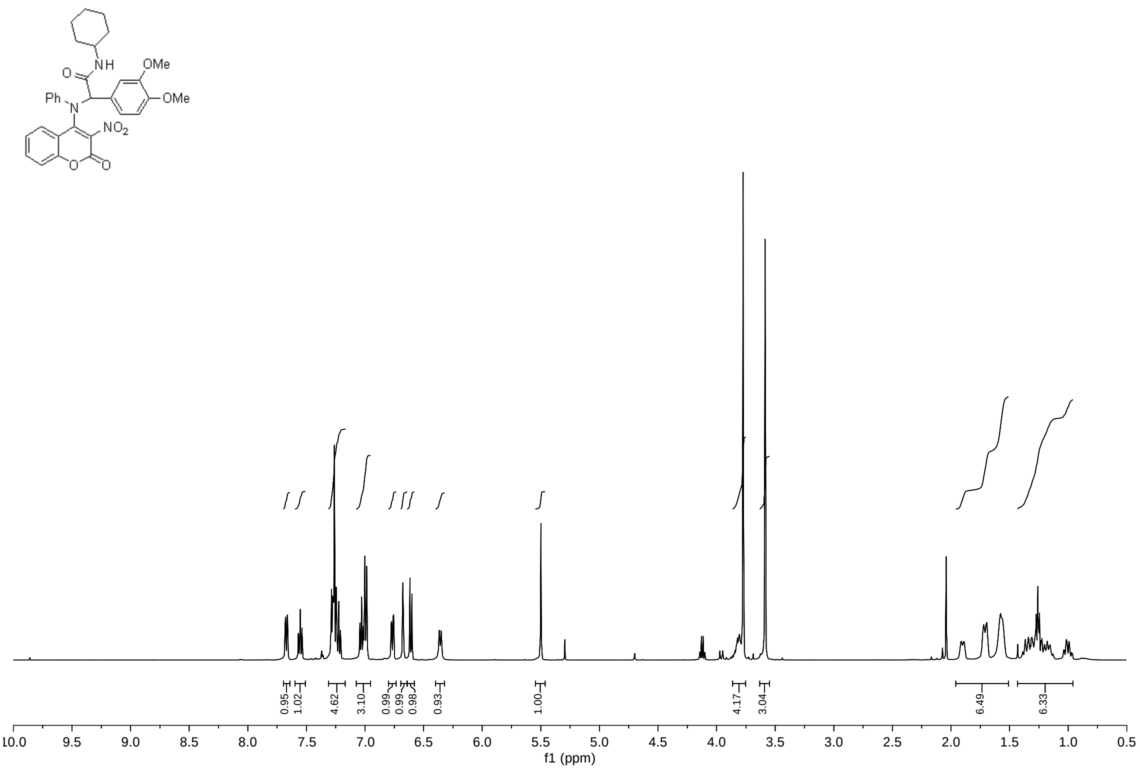

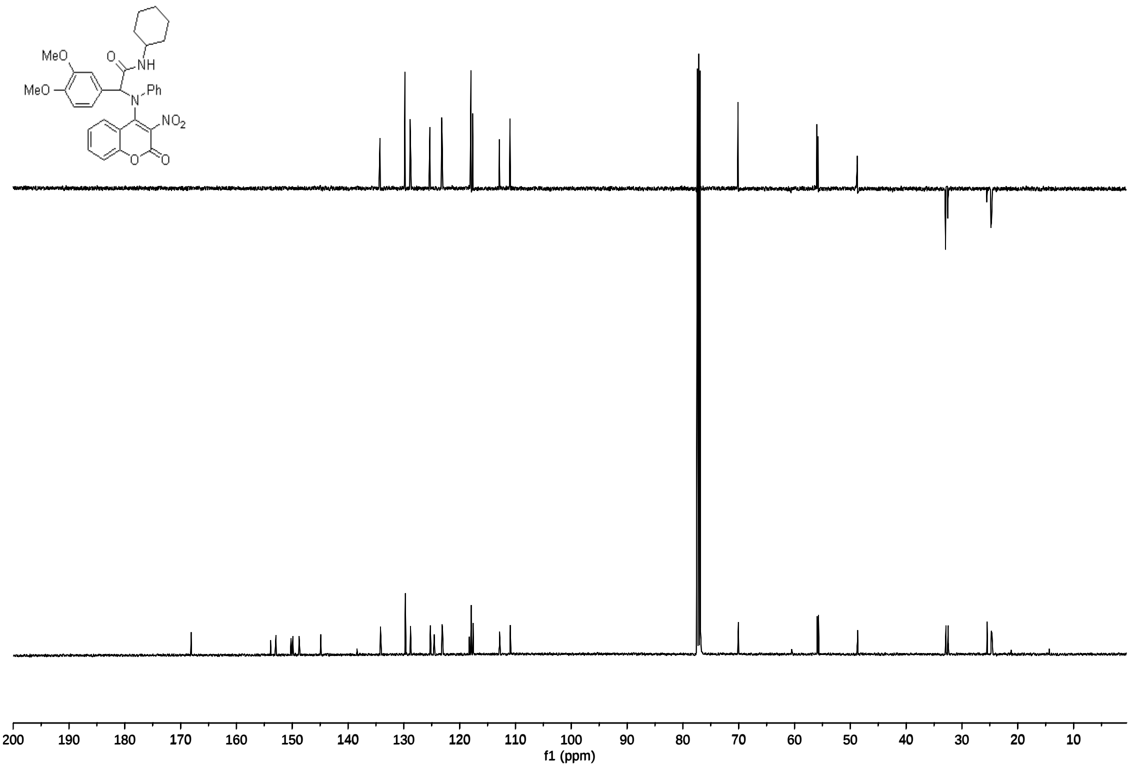

Methyl *N*-(2-(cyclohexylamino)-2-oxo-1-phenylethyl)-*N*-(3-nitro-2-oxo-2*H*-chromen-4-yl)glycinate (17l).

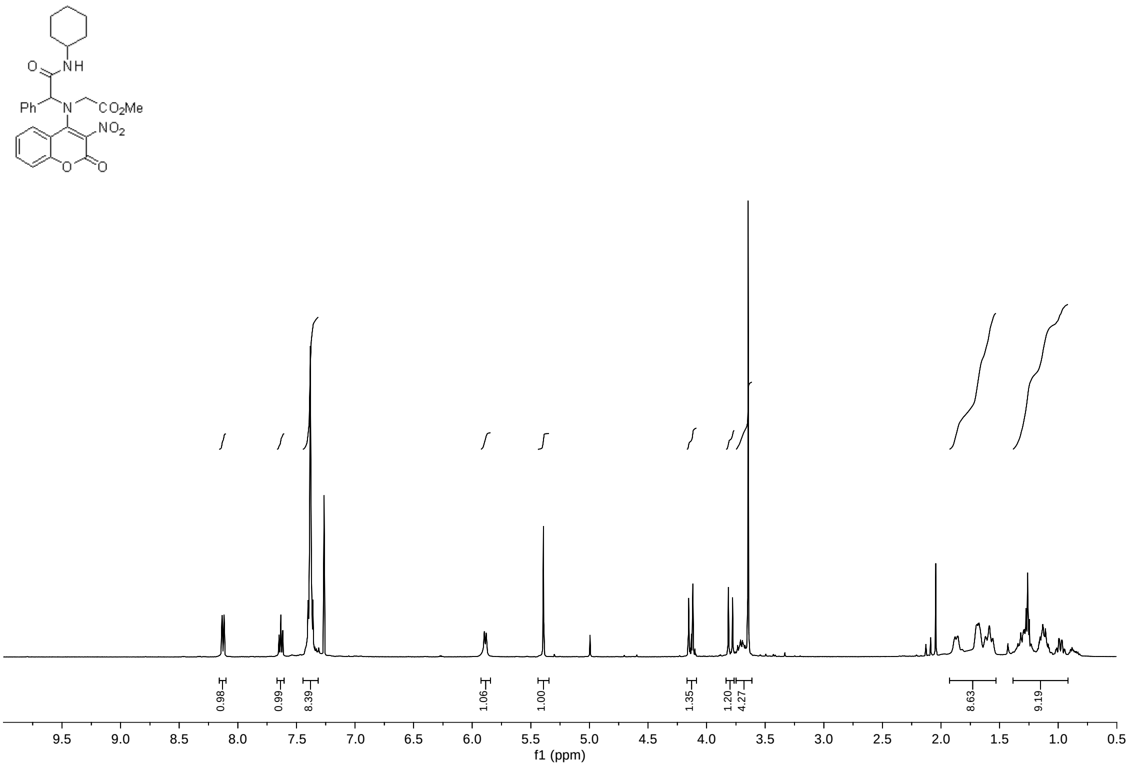

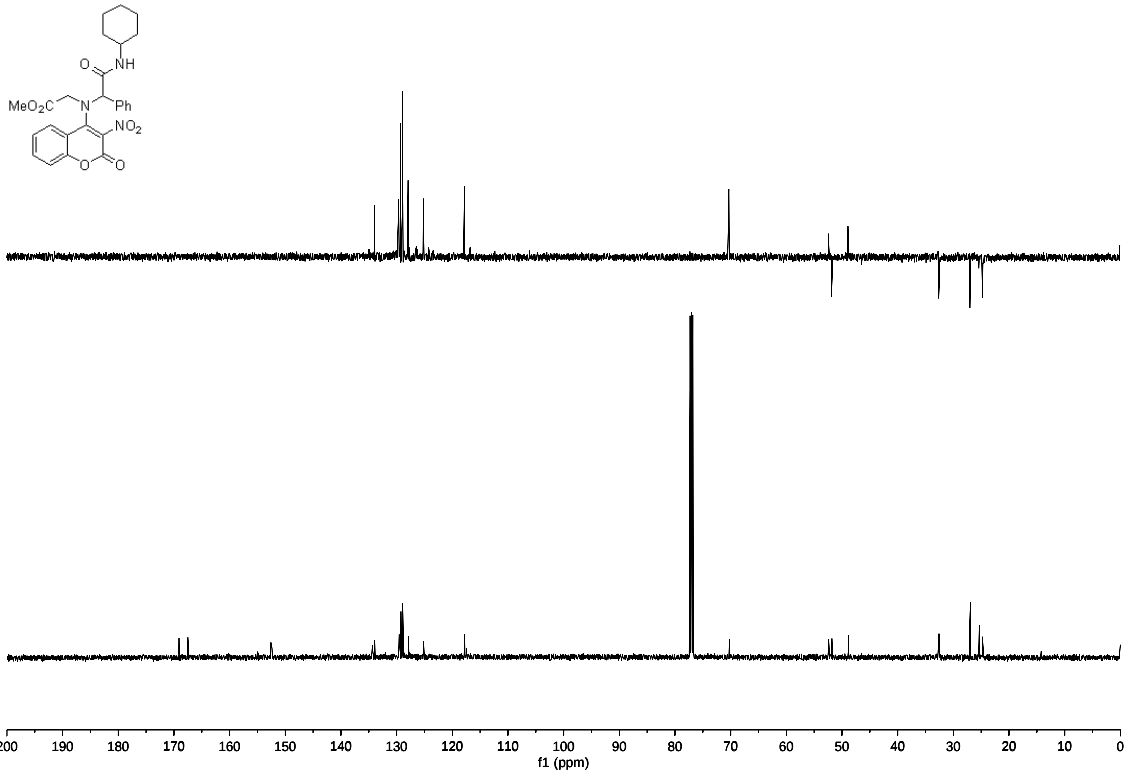

Methyl *N*-(2-(cyclohexylamino)-2-oxo-1-(*p*-tolyl)ethyl)-*N*-(3-nitro-2-oxo-2*H*-chromen-4-yl)glycinate (17m).

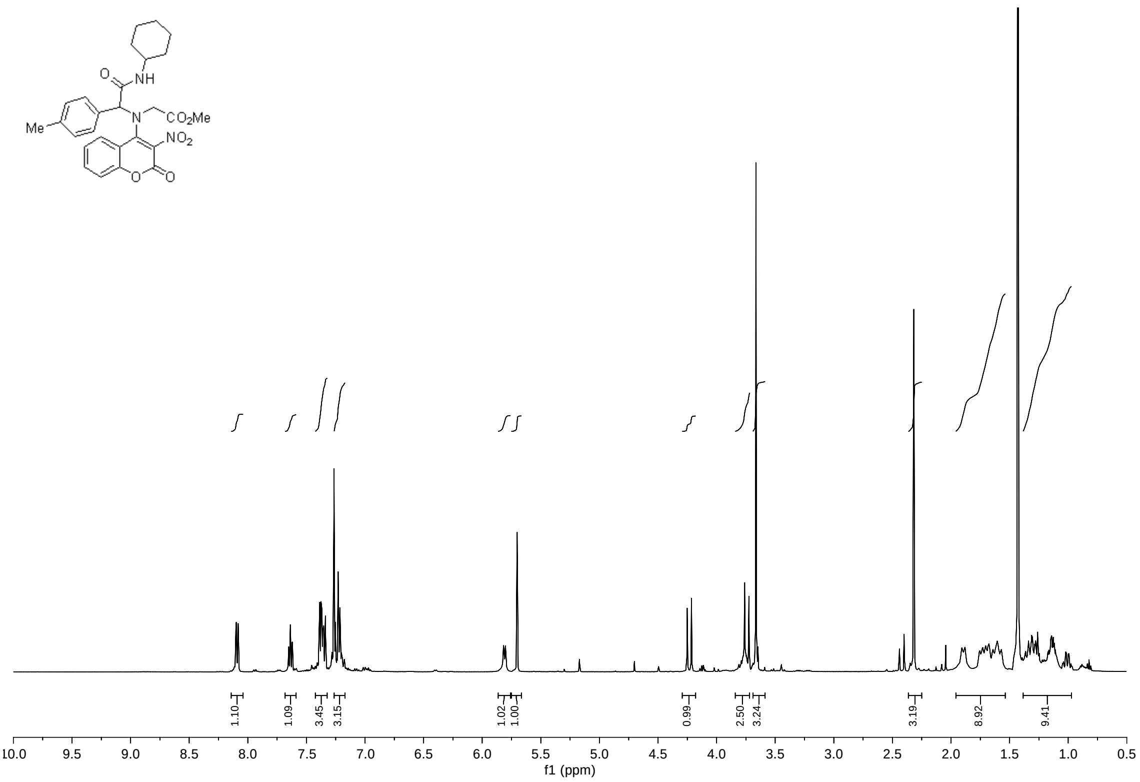

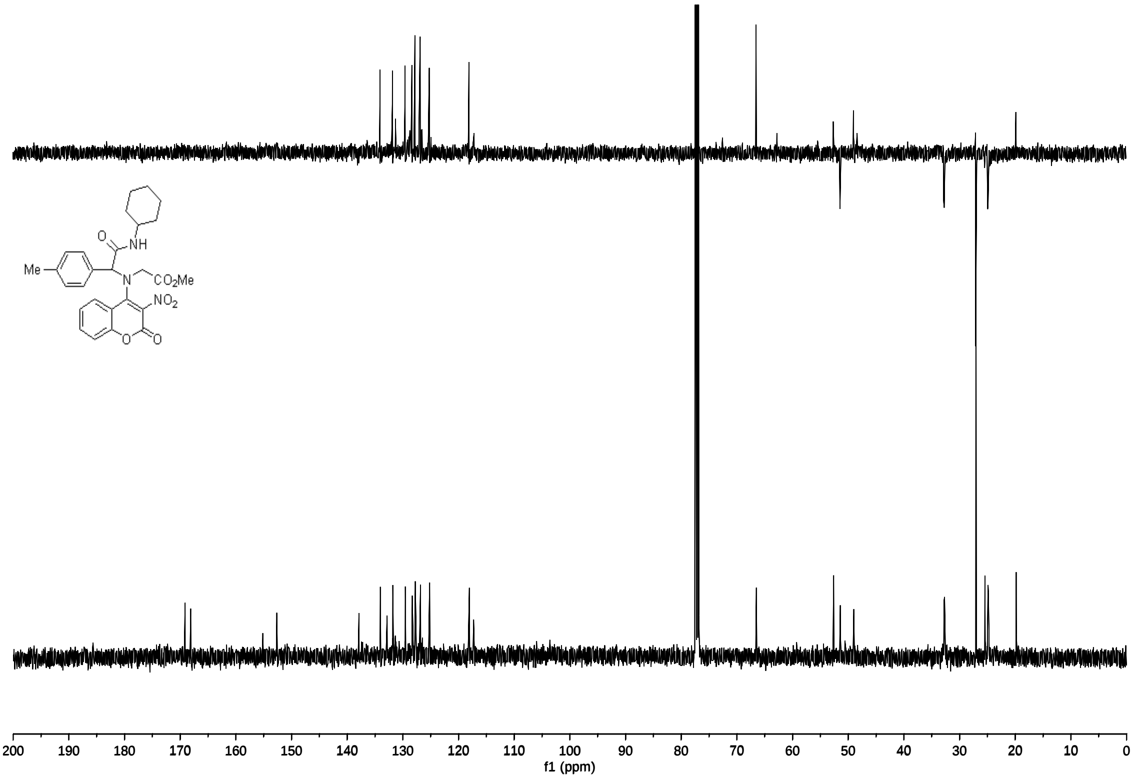

Methyl *N*-(2-(cyclohexylamino)-2-oxo-1-(4-(trifluoromethyl)phenyl)ethyl)-*N*-(3-nitro-2-oxo-2*H*-chromen-4-yl)glycinate (17n).

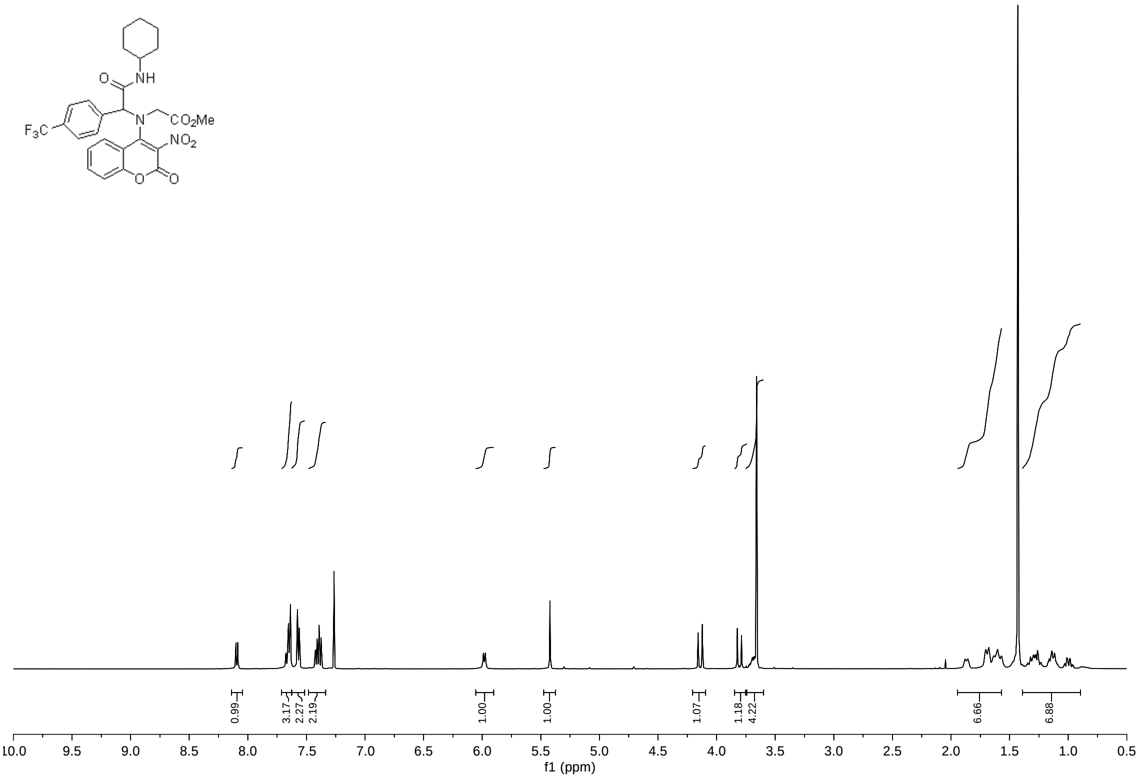

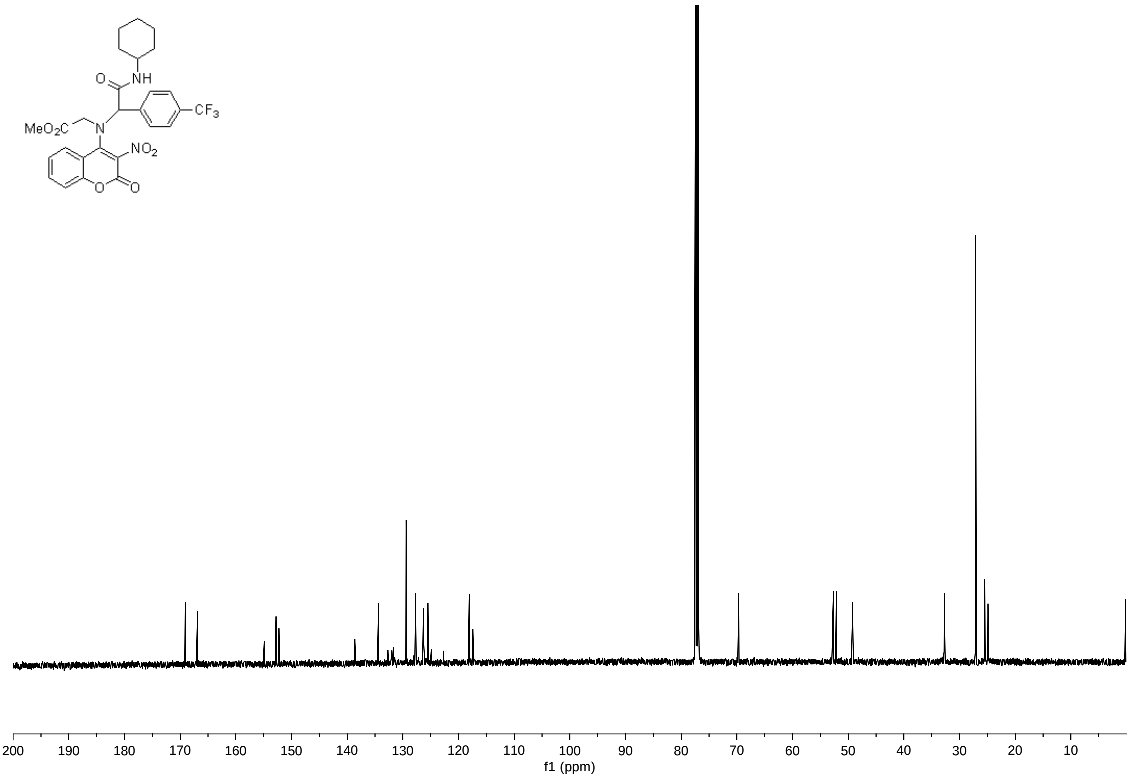

Methyl *N*-(2-(*tert*-butylamino)-2-oxo-1-phenylethyl)-*N*-(3-nitro-2-oxo-2H-chromen-4-yl)glycinate (17o).

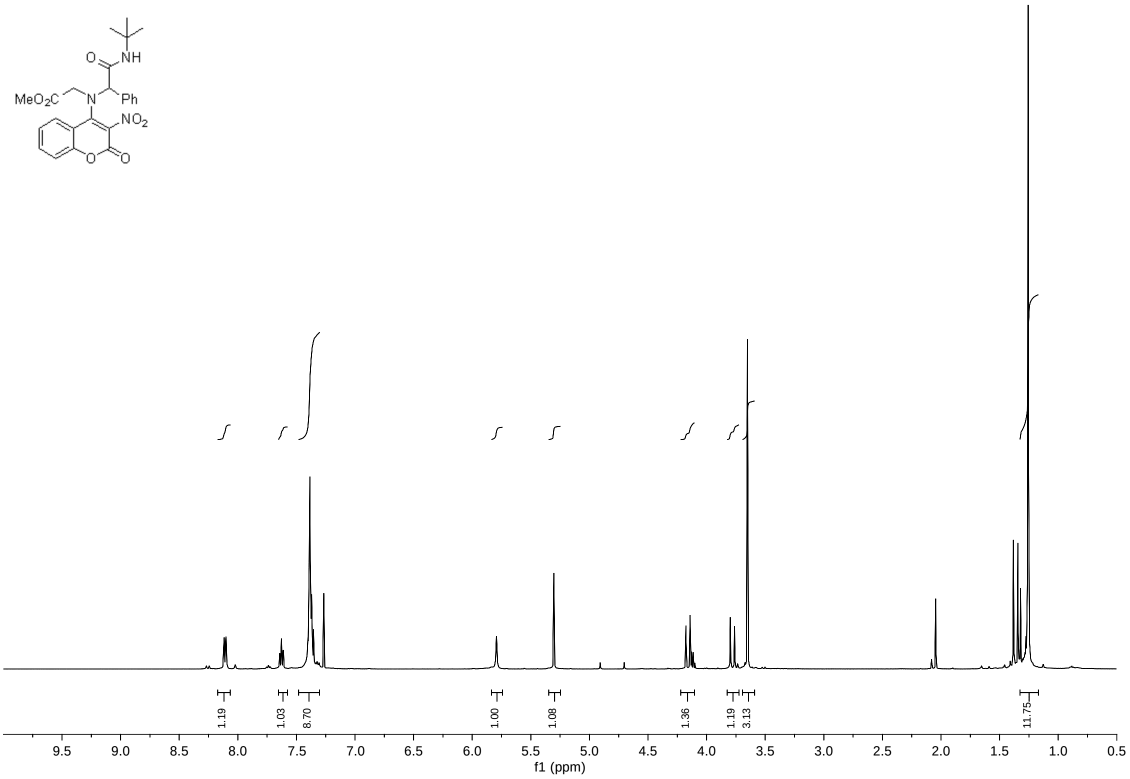

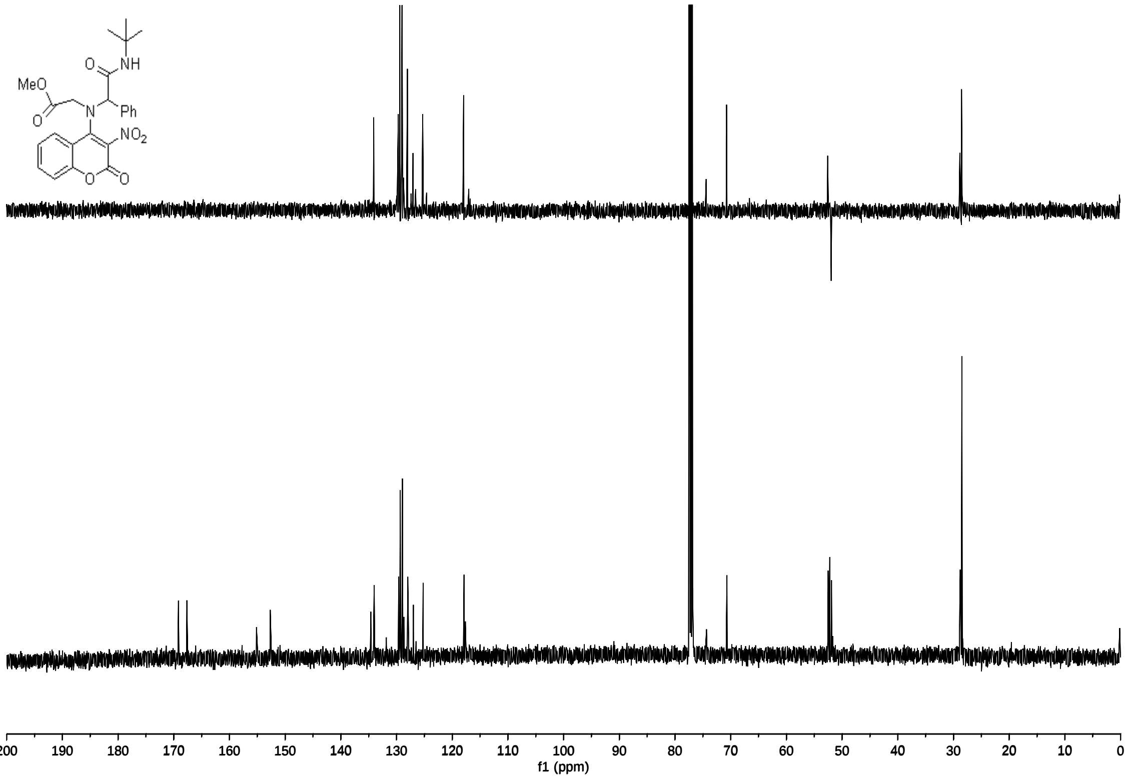

Methyl *N*-(3-nitro-2-oxo-2*H*-chromen-4-yl)-*N*-(2-oxo-2-(pentylamino)-1-phenylethyl) glycinate (17g).

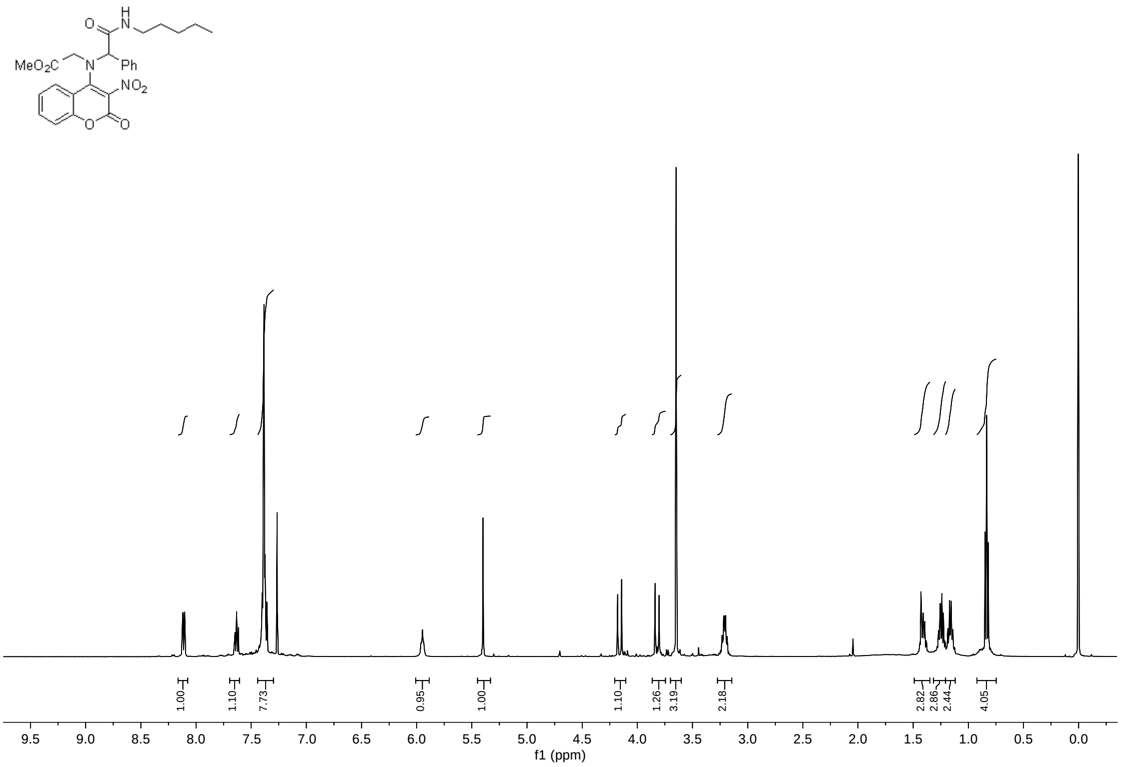

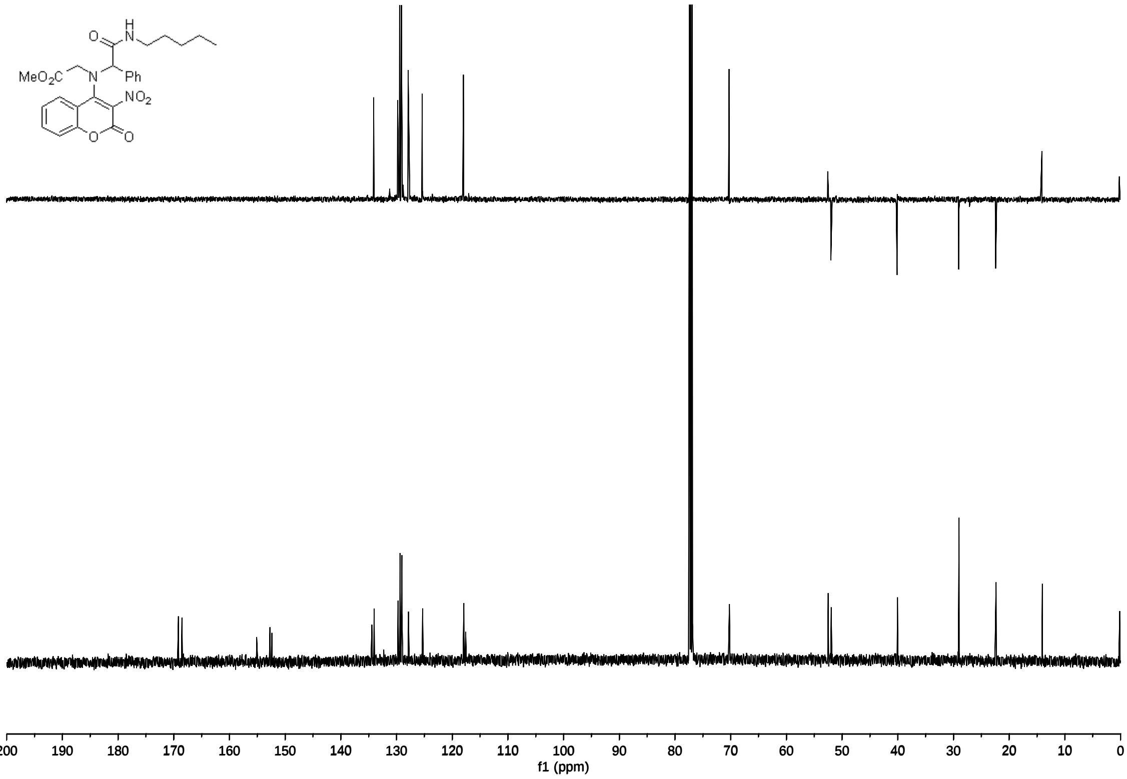

Methyl *N*-(2-(benzylamino)-2-oxo-1-phenylethyl)-*N*-(3-nitro-2-oxo-2*H*-chromen-4-yl)glycinate (17q)

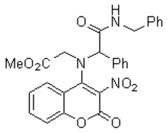

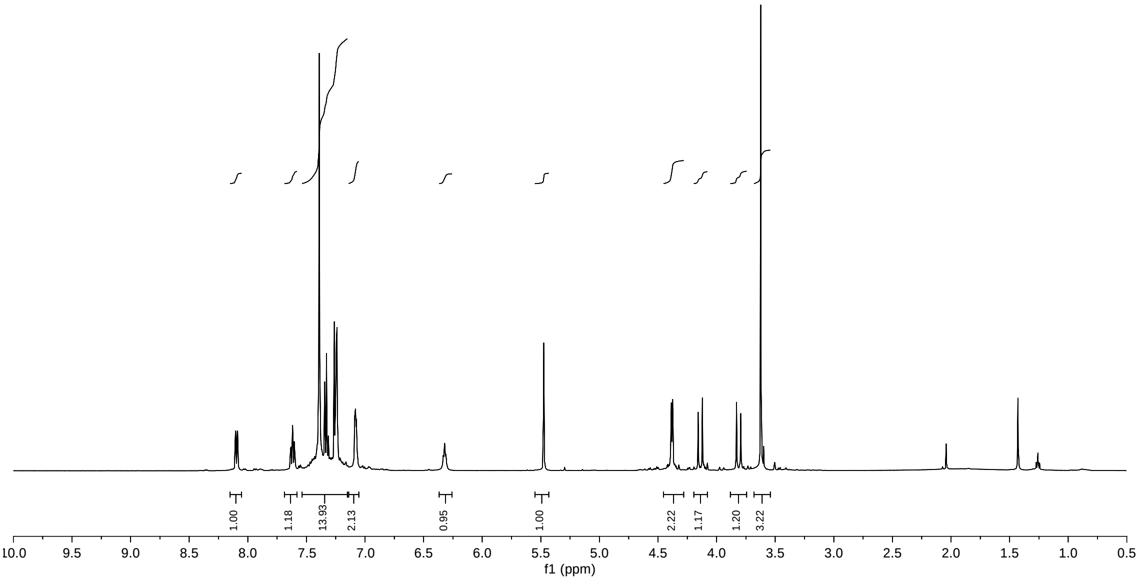

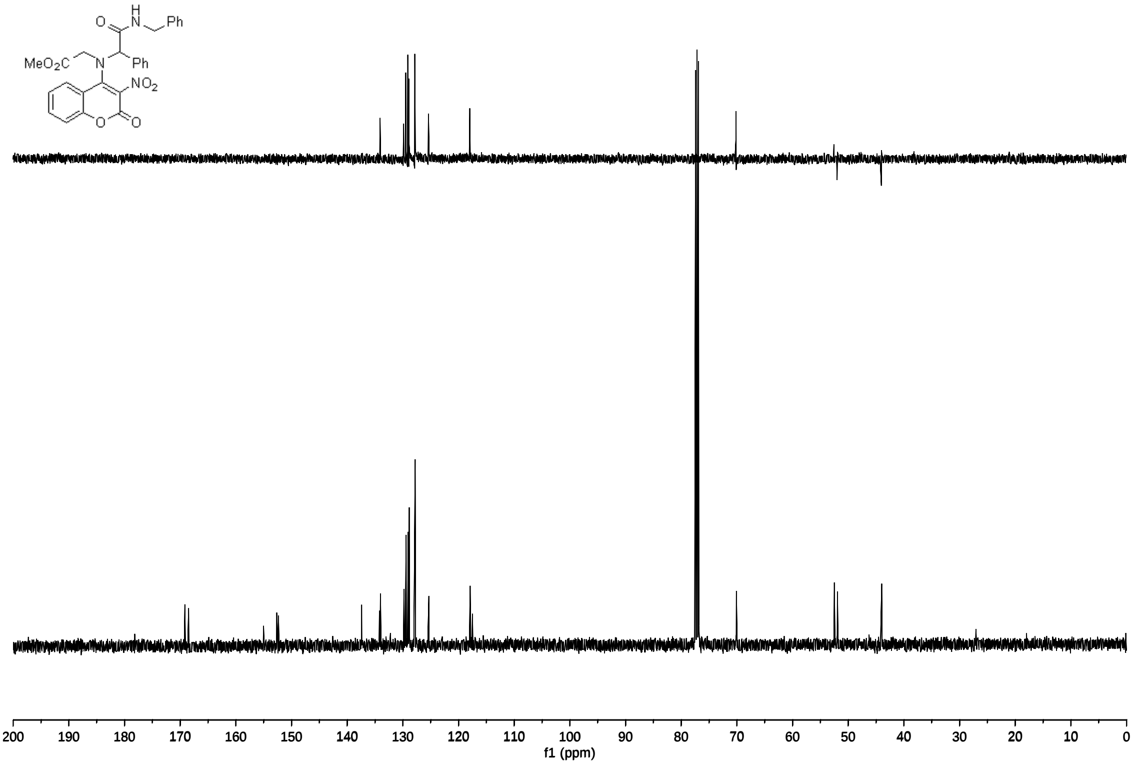

Methyl *N*-(2-(benzylamino)-2-oxo-1-(*p*-tolyl)ethyl)-*N*-(3-nitro-2-oxo-2*H*-chromen-4-yl)glycinate (17r).

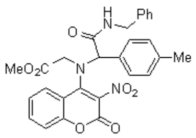

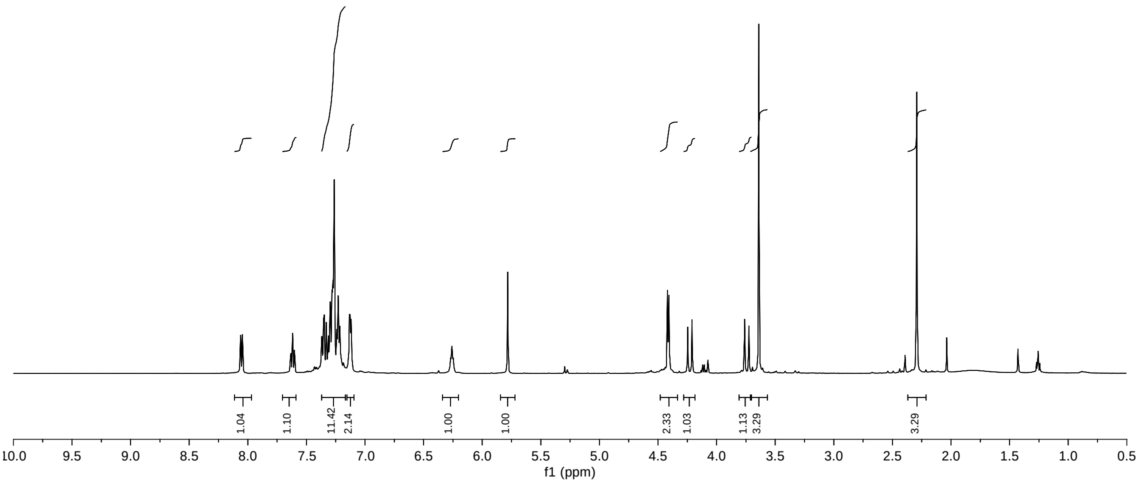

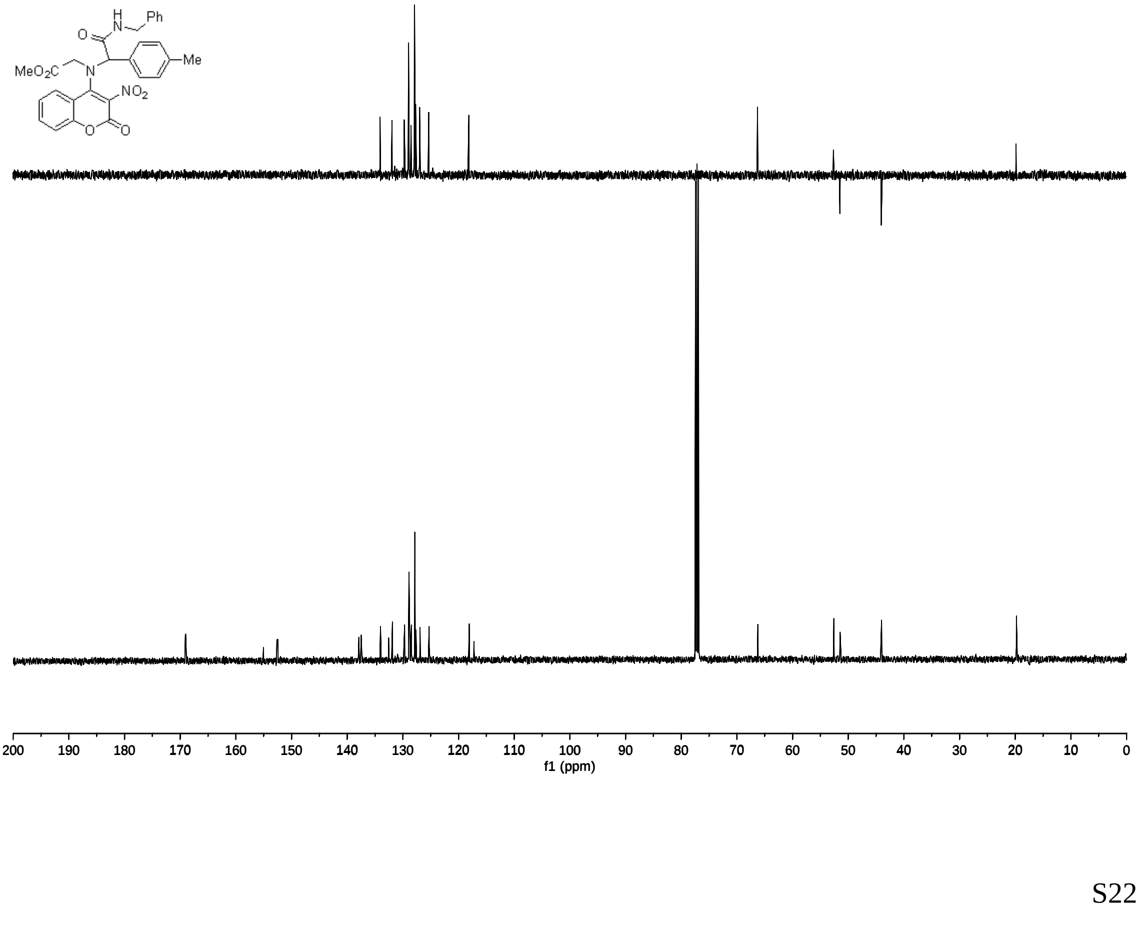

Ethyl 3-((2-(cyclohexylamino)-2-oxo-1-phenylethyl)(3-nitro-2-oxo-2*H*-chromen-4-yl)amino)propanoate (17s).

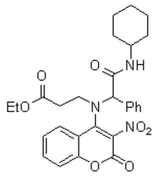

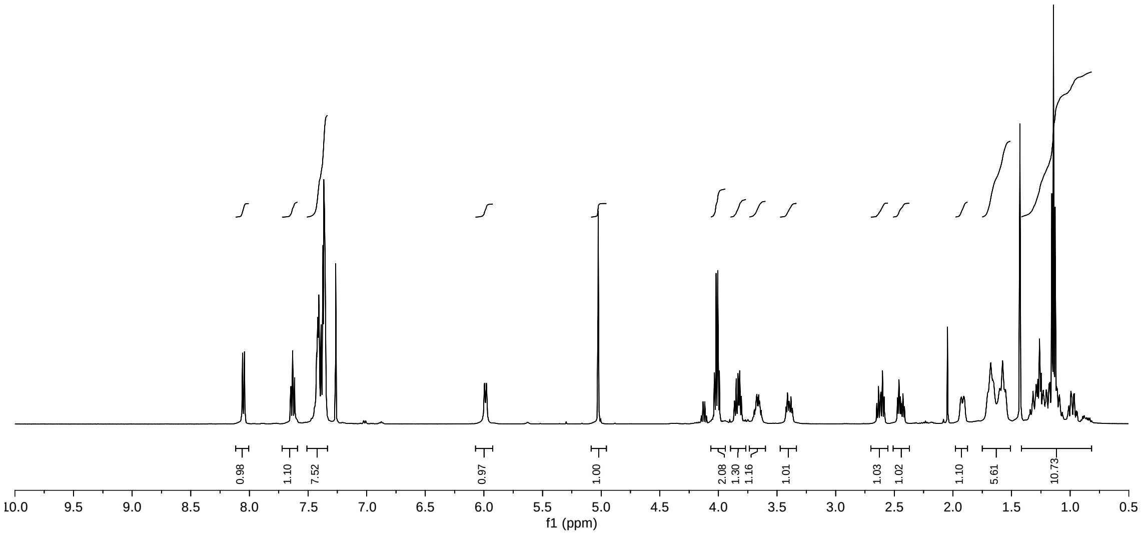

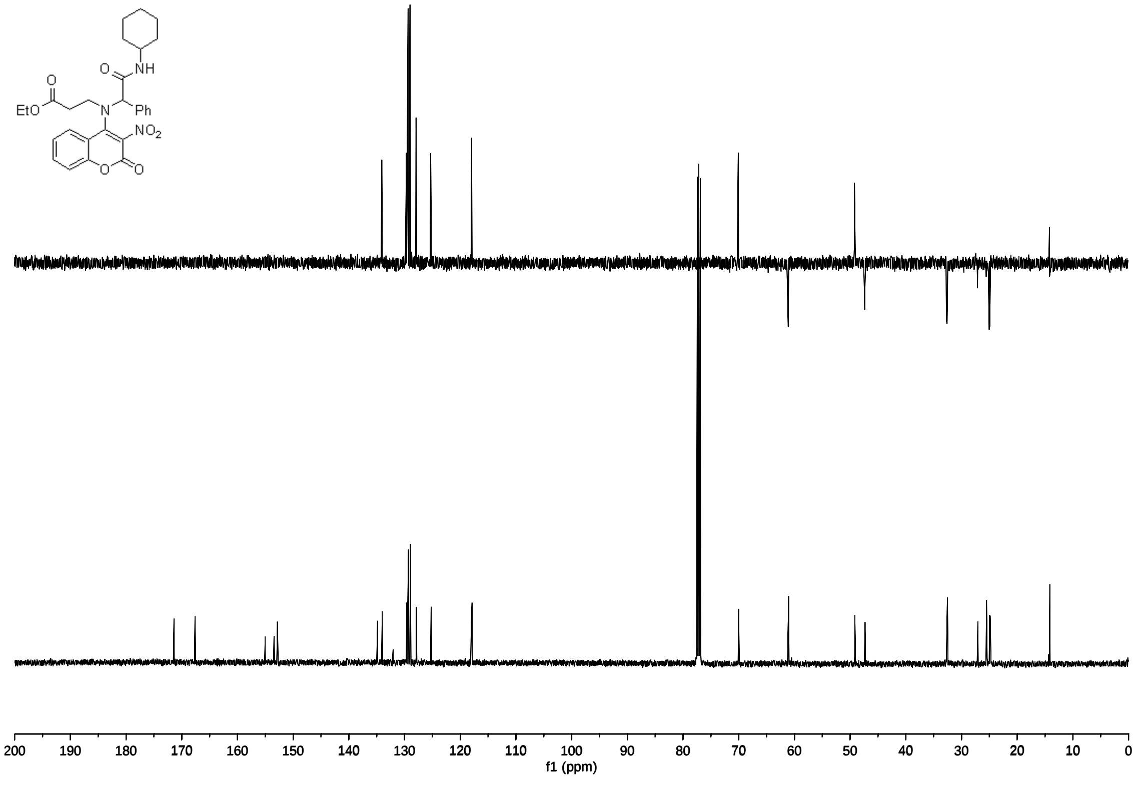

1-Benzyl-2-phenyl-1,4-dihydro-2*H*-chromeno[3,4-*b*]pyrazine-3,5-dione (19a).

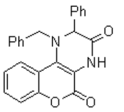

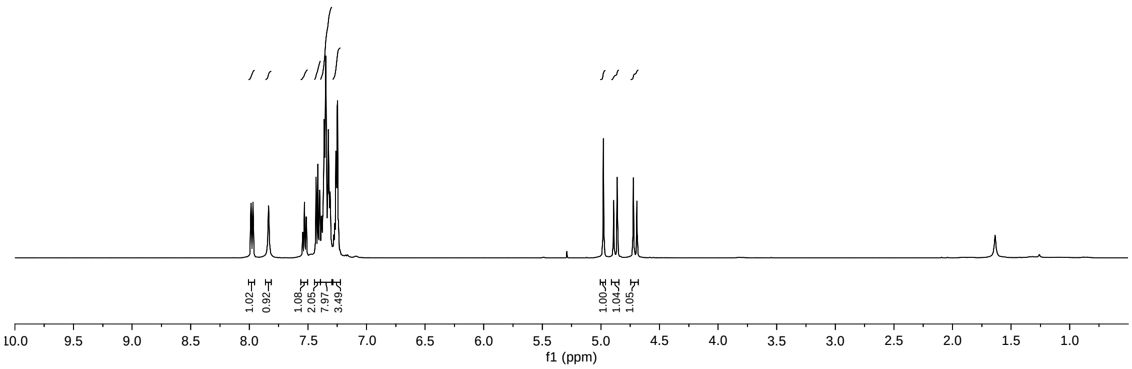

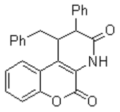

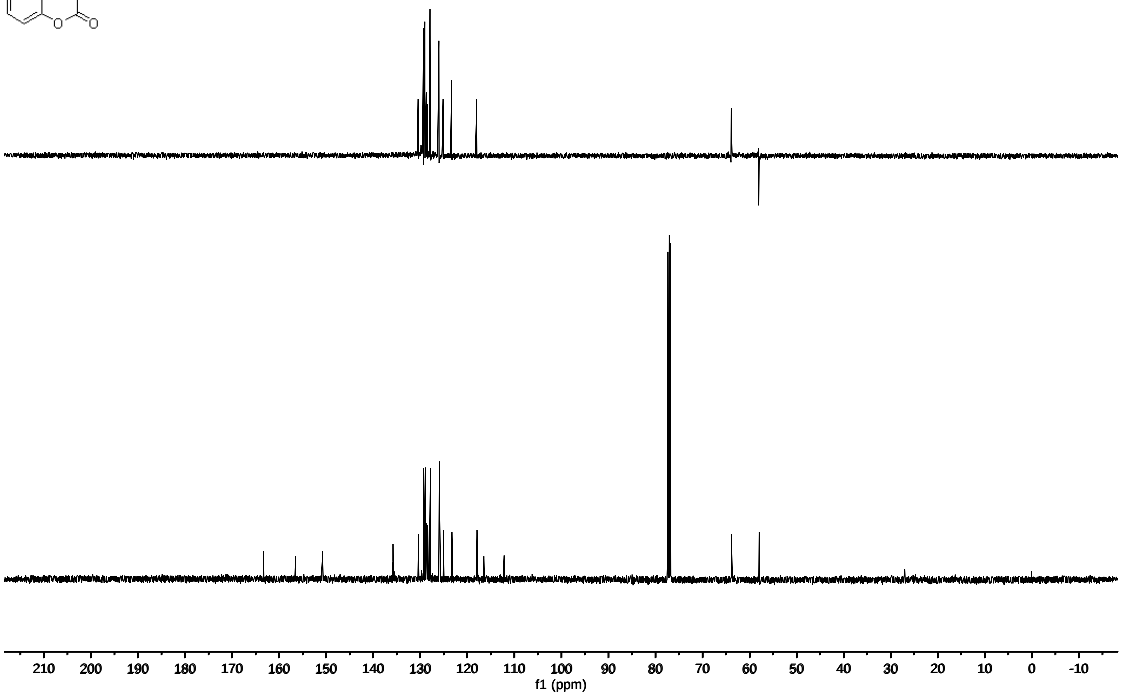

1-Benzyl-2-(2-bromophenyl)-1,4-dihydro-2*H*-chromeno[3,4-*b*]pyrazine-3,5-dione (19b)

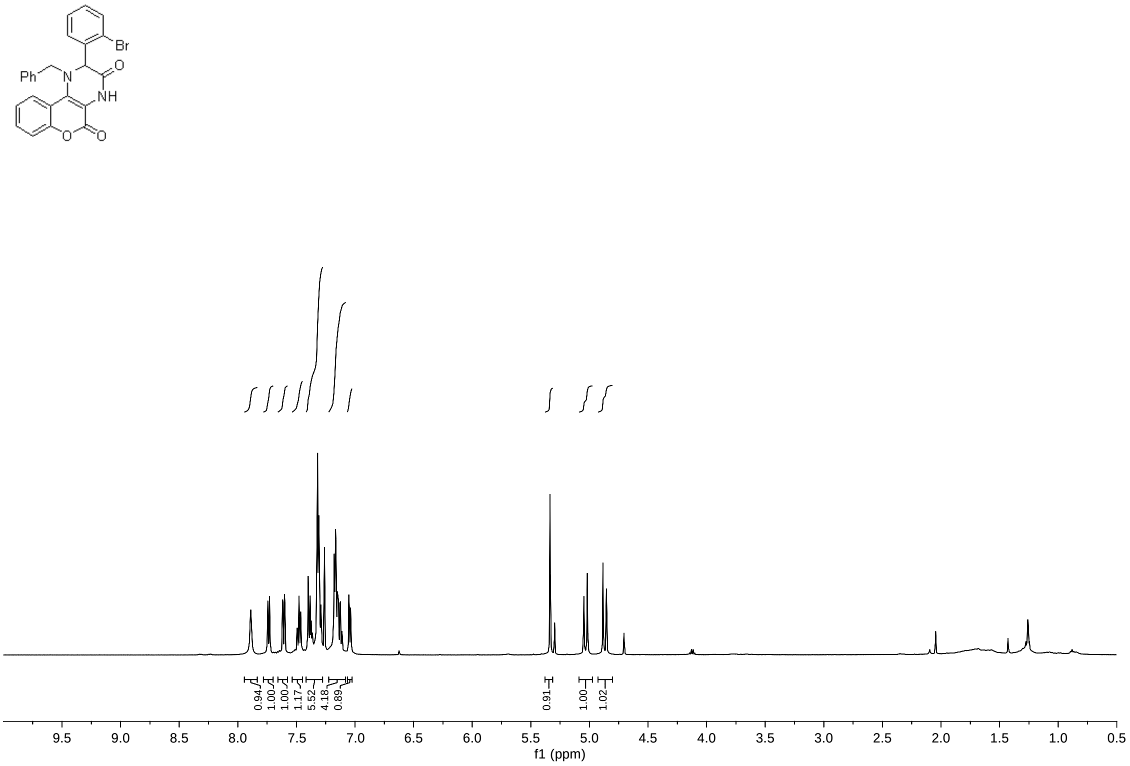

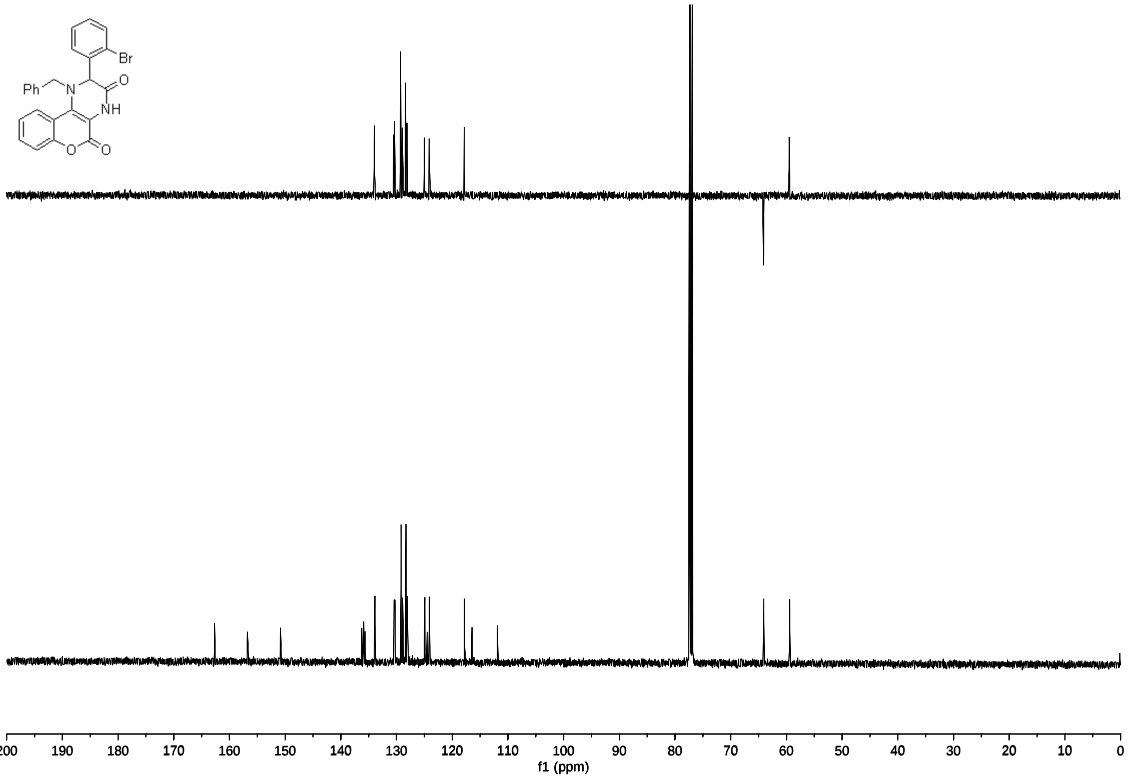

1-Benzyl-2-(*p*-tolyl)-1,4-dihydro-2*H*-chromeno[3,4-*b*]pyrazine-3,5-dione (19c).

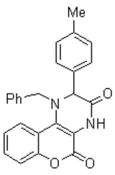

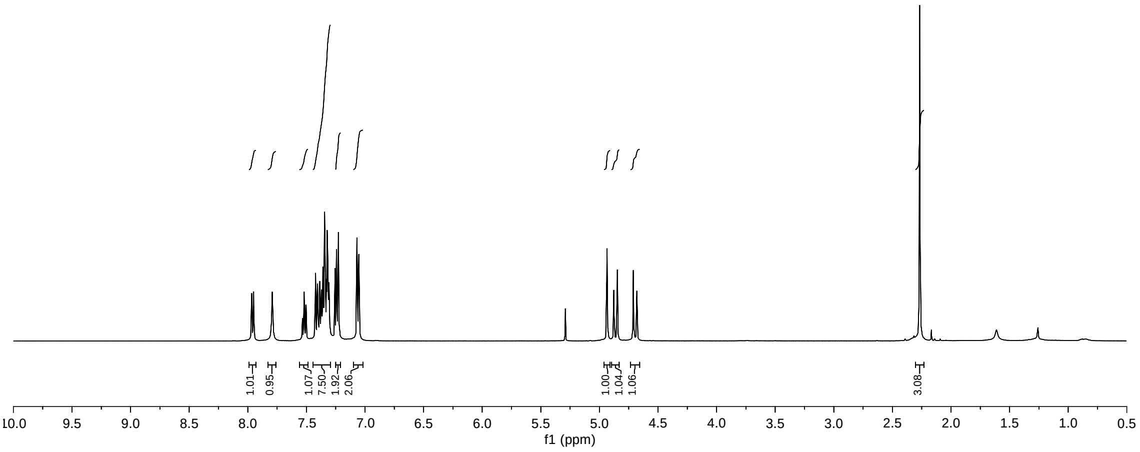

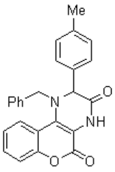

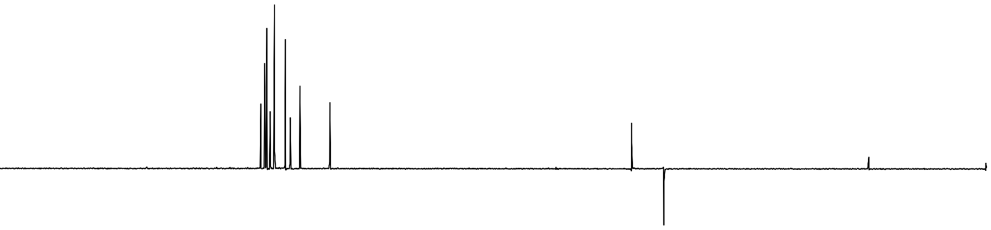

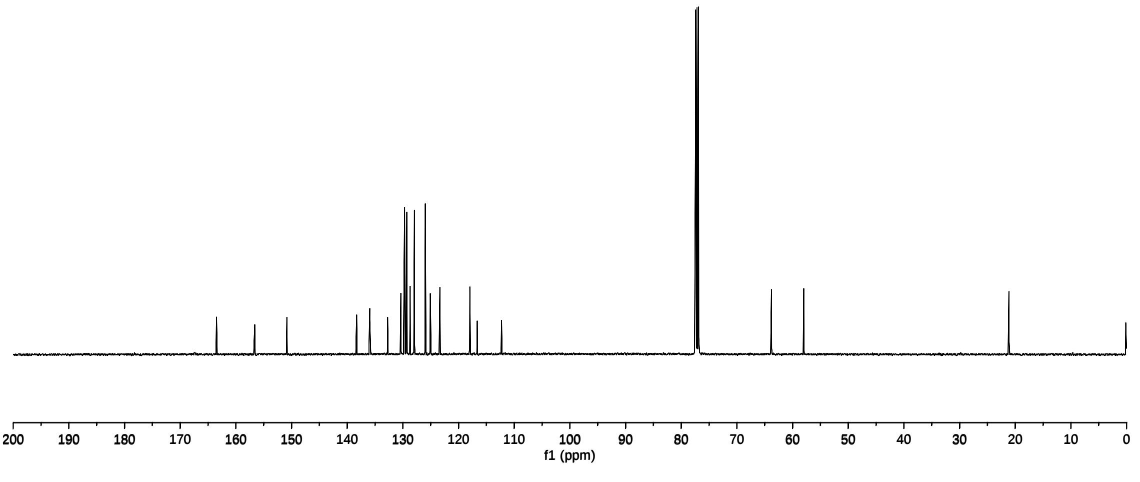

1-Benzyl-2-(4-(trifluoromethyl)phenyl)-1,4-dihydro-2*H*-chromeno[3,4-*b*]pyrazine-3,5-dione (19d).

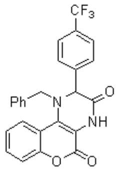

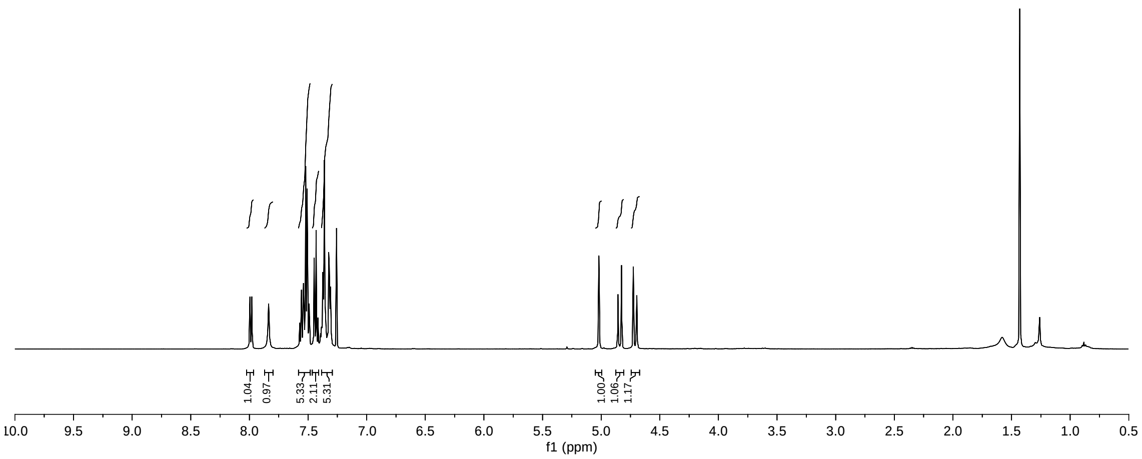

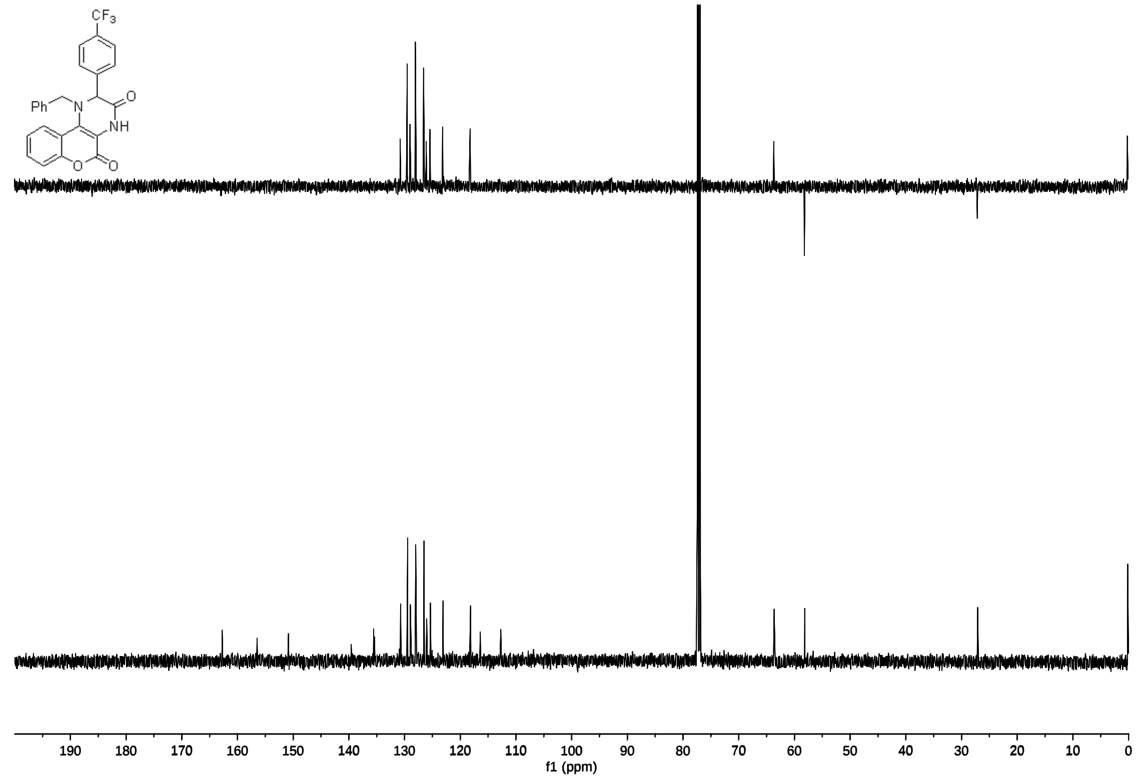

1-(Benzo[*d*][1,3]dioxol-5-ylmethyl)-2-phenyl-1,4-dihydro-2*H*-chromeno[3,4-*b*]pyrazine-3,5-dione (19e).

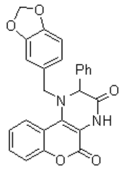

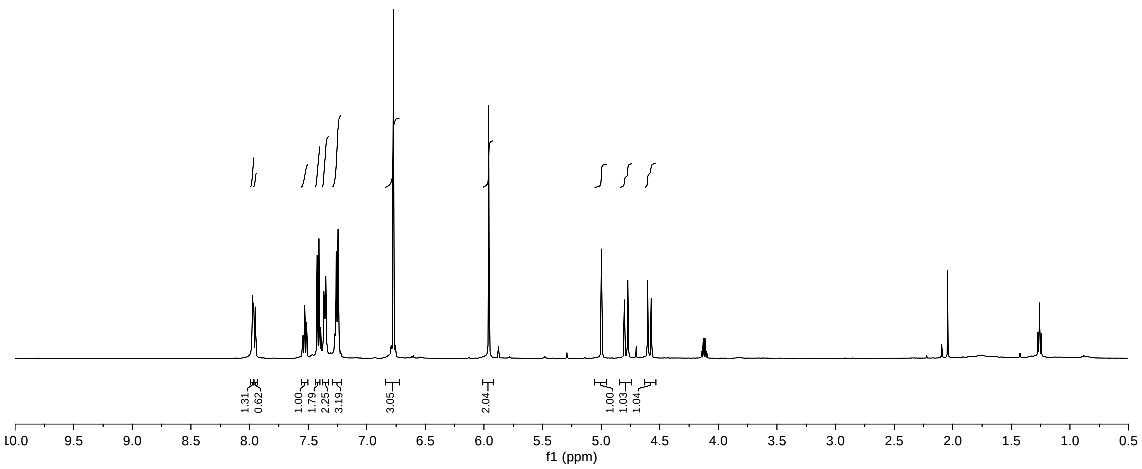

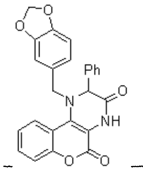

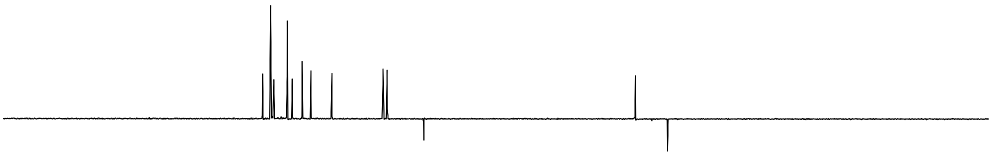

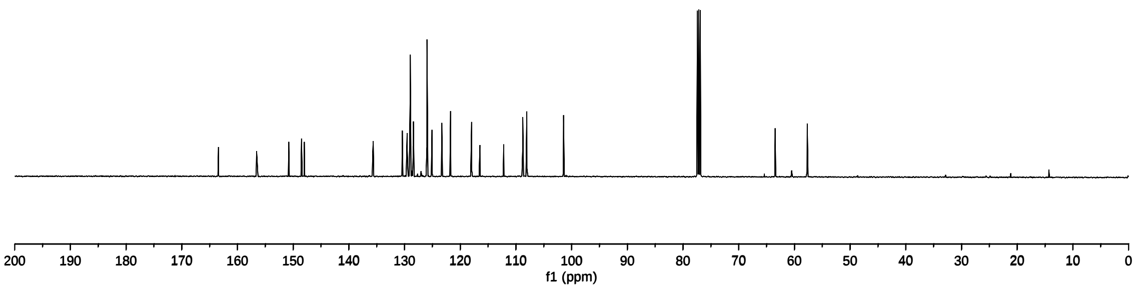

1-(Benzo[*d*][1,3]dioxol-5-ylmethyl)-2-(*p*-tolyl)-1,4-dihydro-2*H*-chromeno[3,4-*b*]pyrazine-3,5-dione (19f).

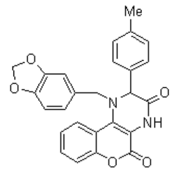

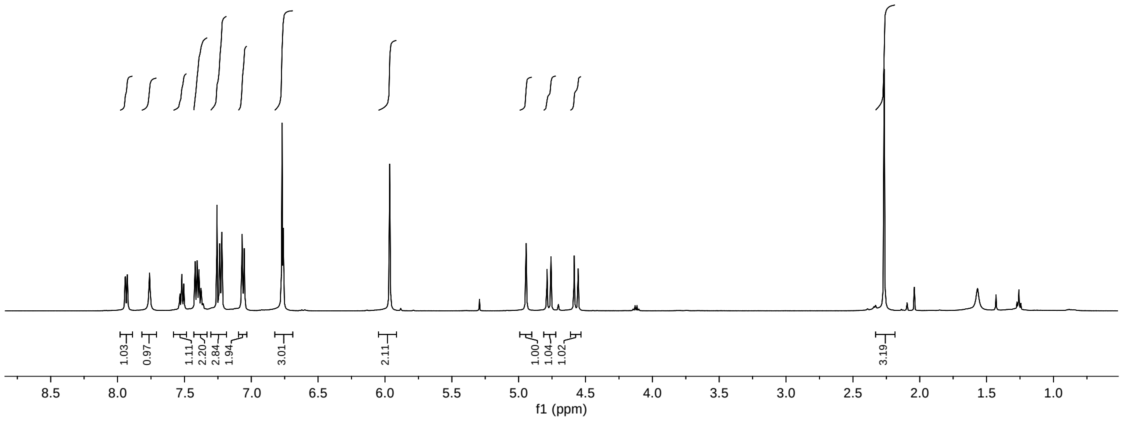

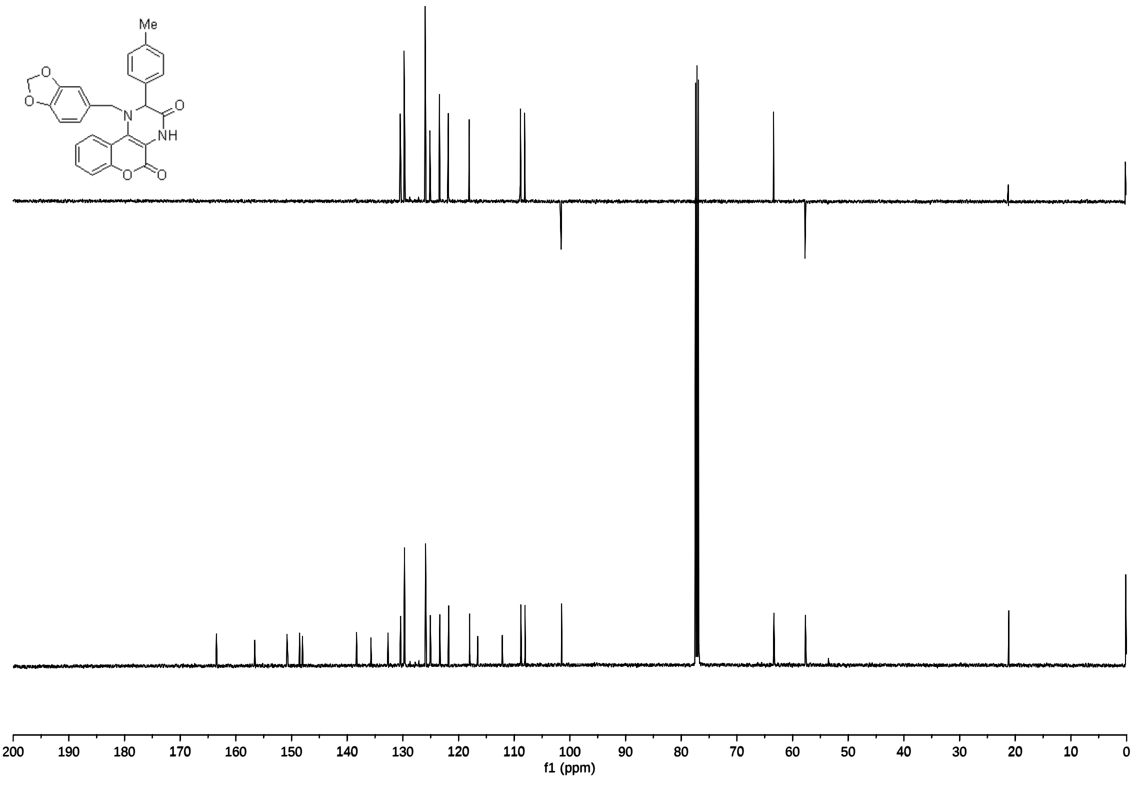

1-(Benzo[*d*][1,3]dioxol-5-ylmethyl)-2-(4-(trifluoromethyl)phenyl)-1,4-dihydro-2*H*-chromeno[3,4-*b*]pyrazine-3,5-dione (19g).

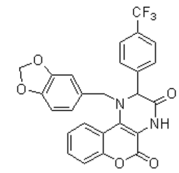

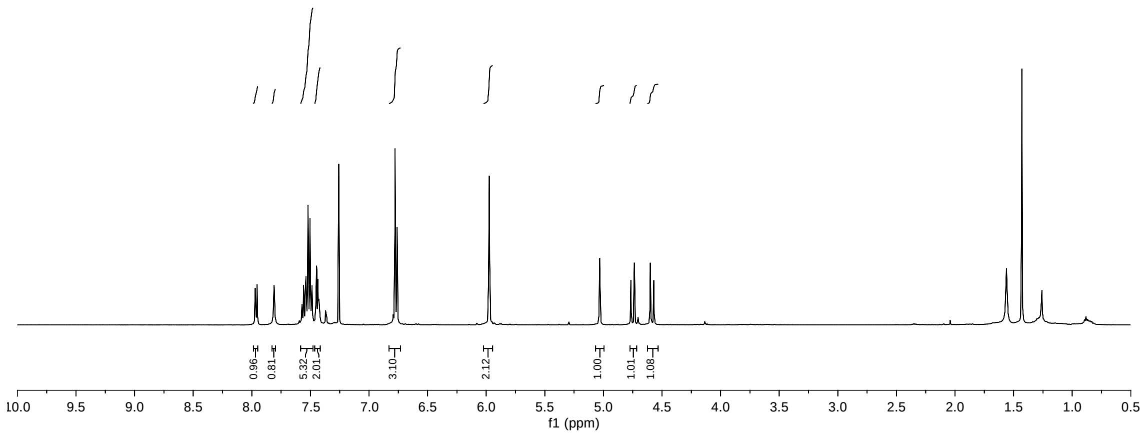

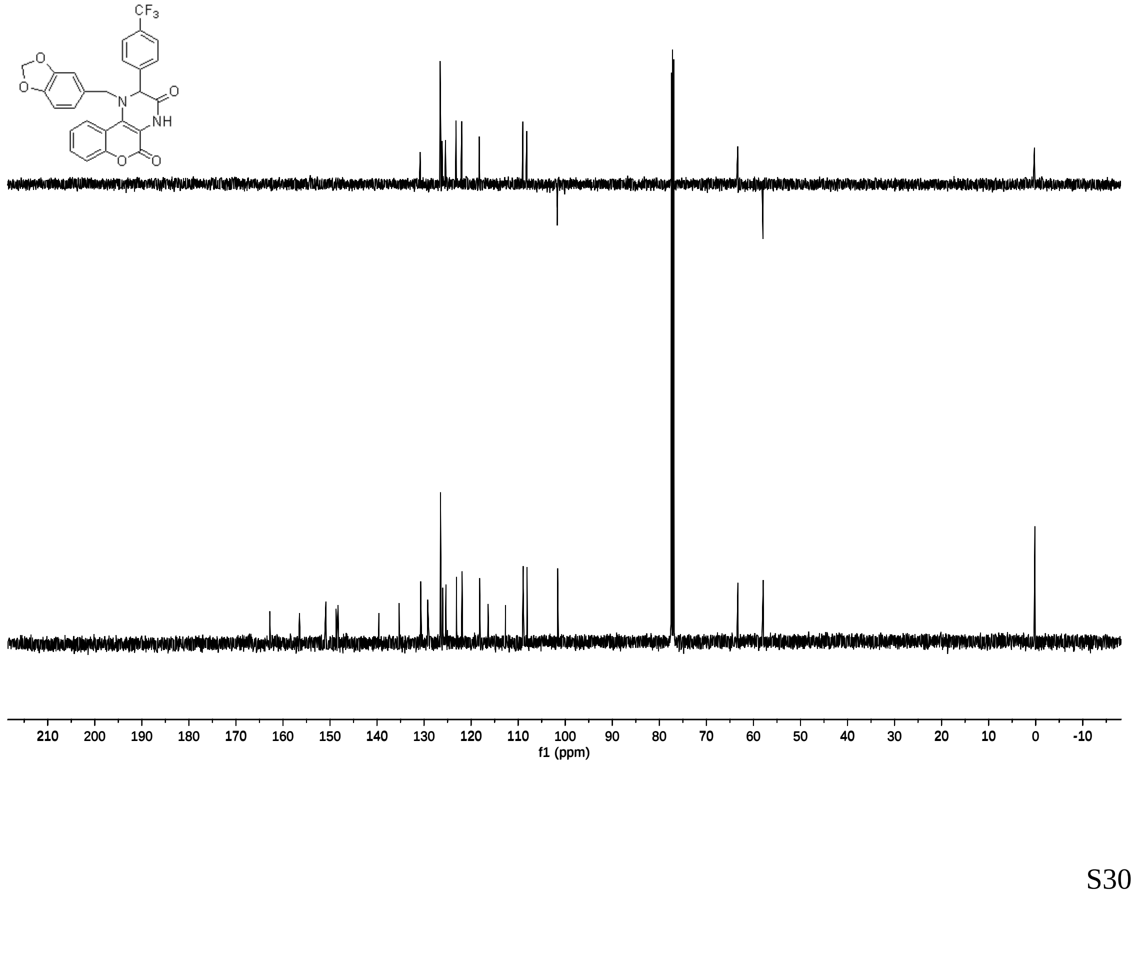

1-Cyclohexyl-2-(*p*-tolyl)-1,4-dihydro-2*H*-chromeno[3,4-*b*]pyrazine-3,5-dione (19h).

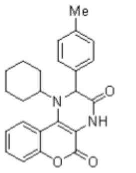

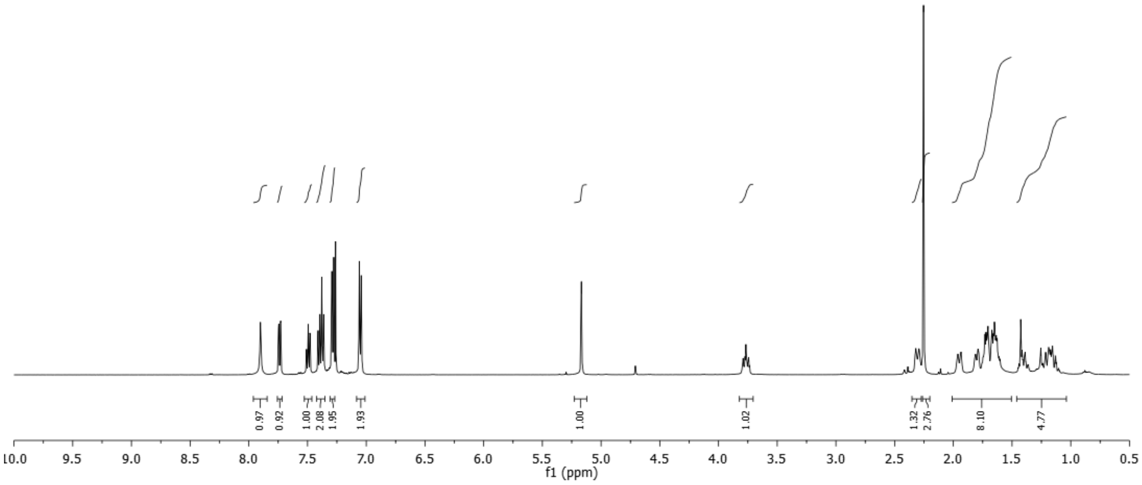

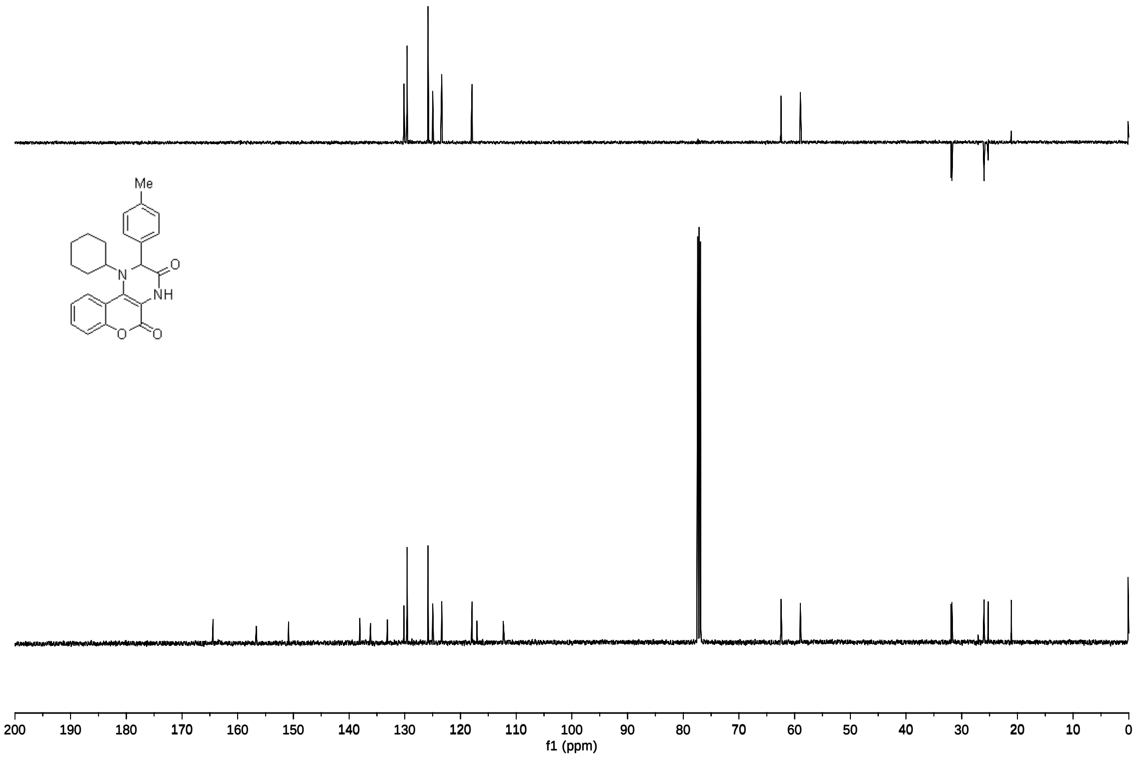

1-Cyclohexyl-2-(4-(trifluoromethyl)phenyl)-1,4-dihydro-2*H*-chromeno[3,4-*b*]pyrazine-3,5-dione (19i)

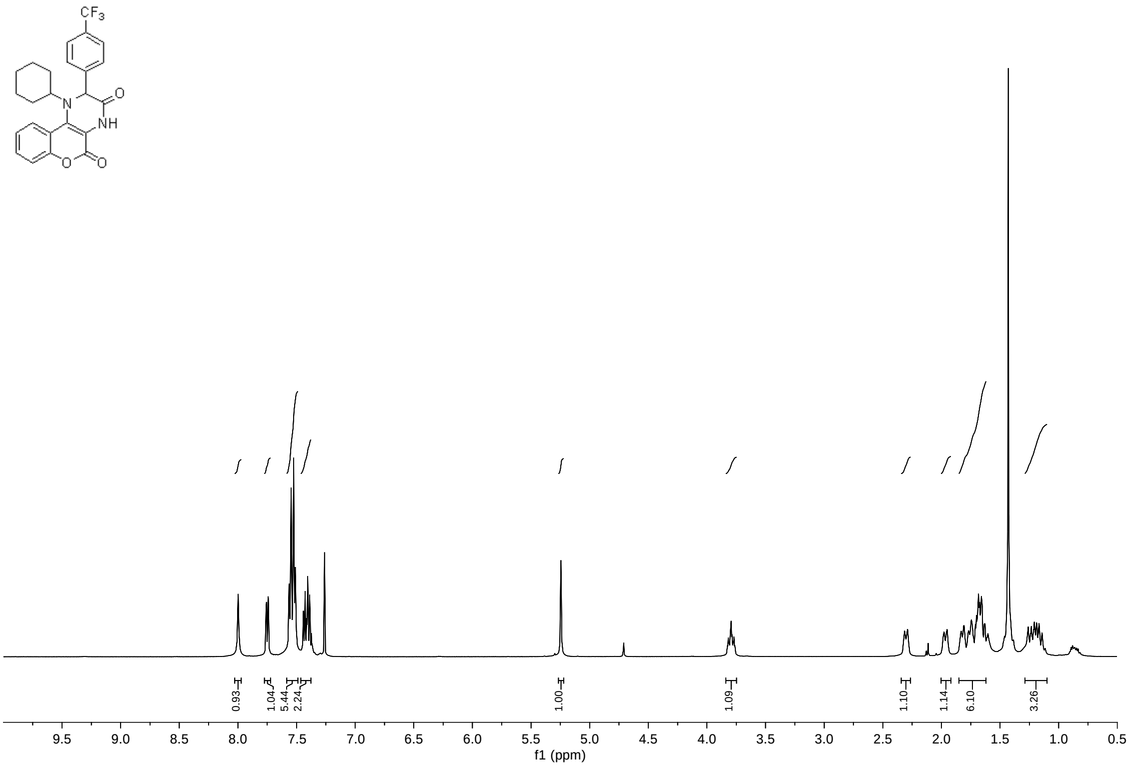

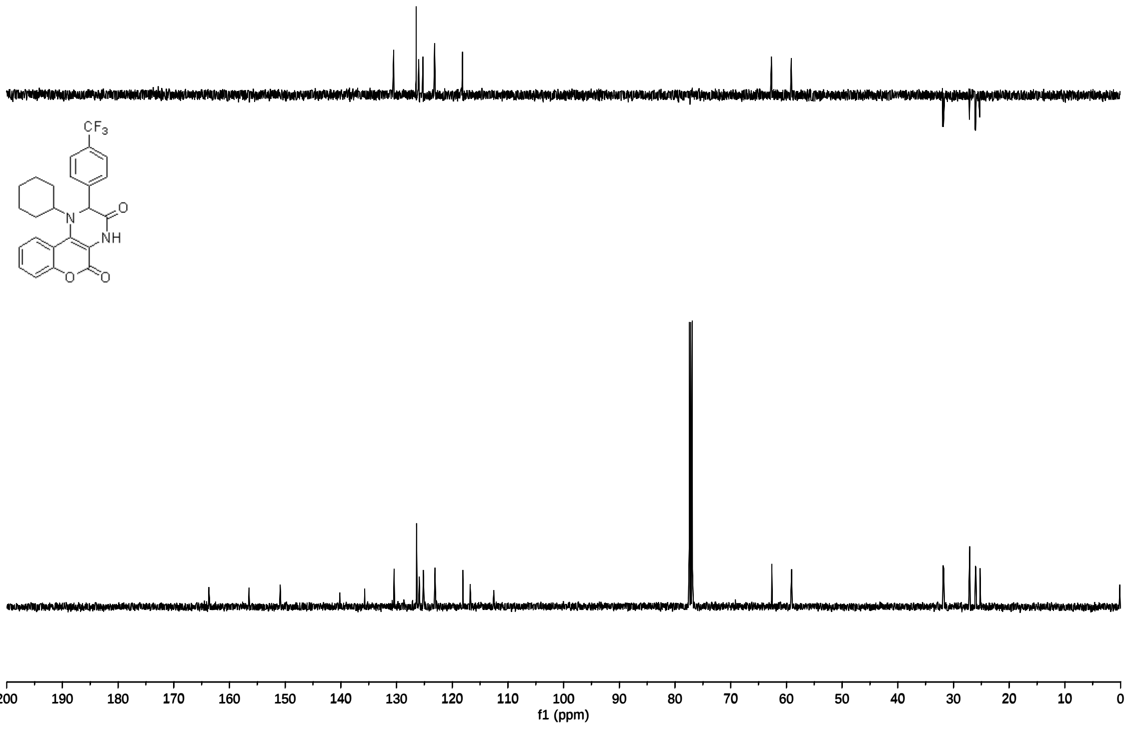

2-((3-Amino-2-oxo-2*H*-chromen-4-yl)(phenyl)amino)-*N*-cyclohexyl-2-phenylacetamide (18j).

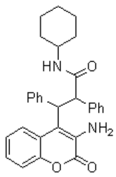

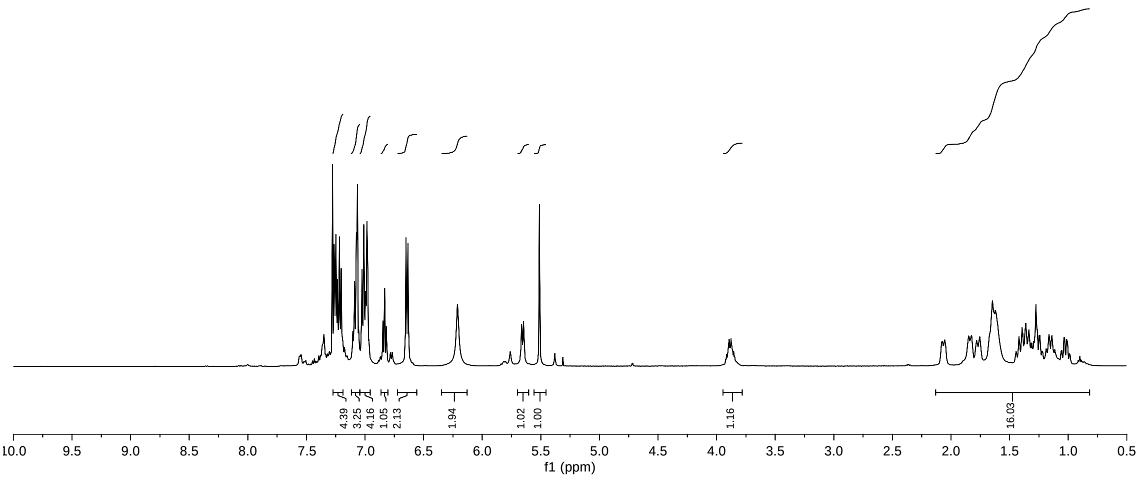

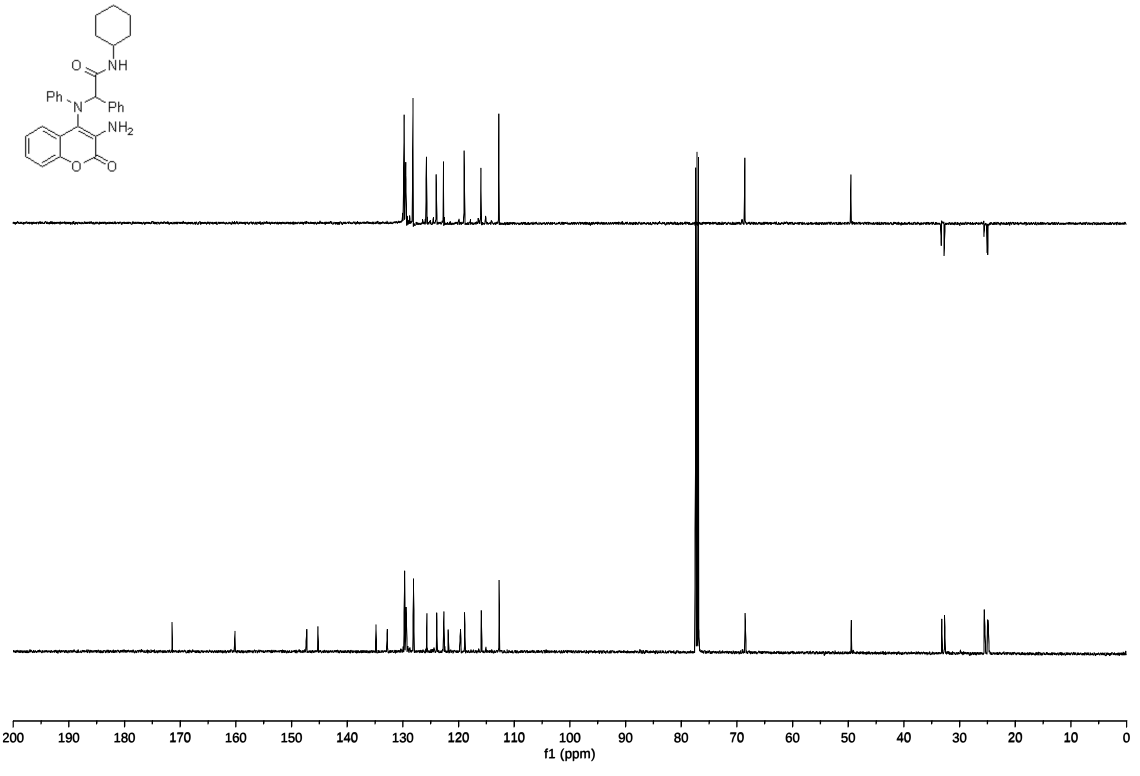

2-((3-Amino-2-oxo-2*H*-chromen-4-yl)(phenyl)amino)-*N*-cyclohexyl-2-(3,4-dimethoxyphenyl)acetamide (18k).

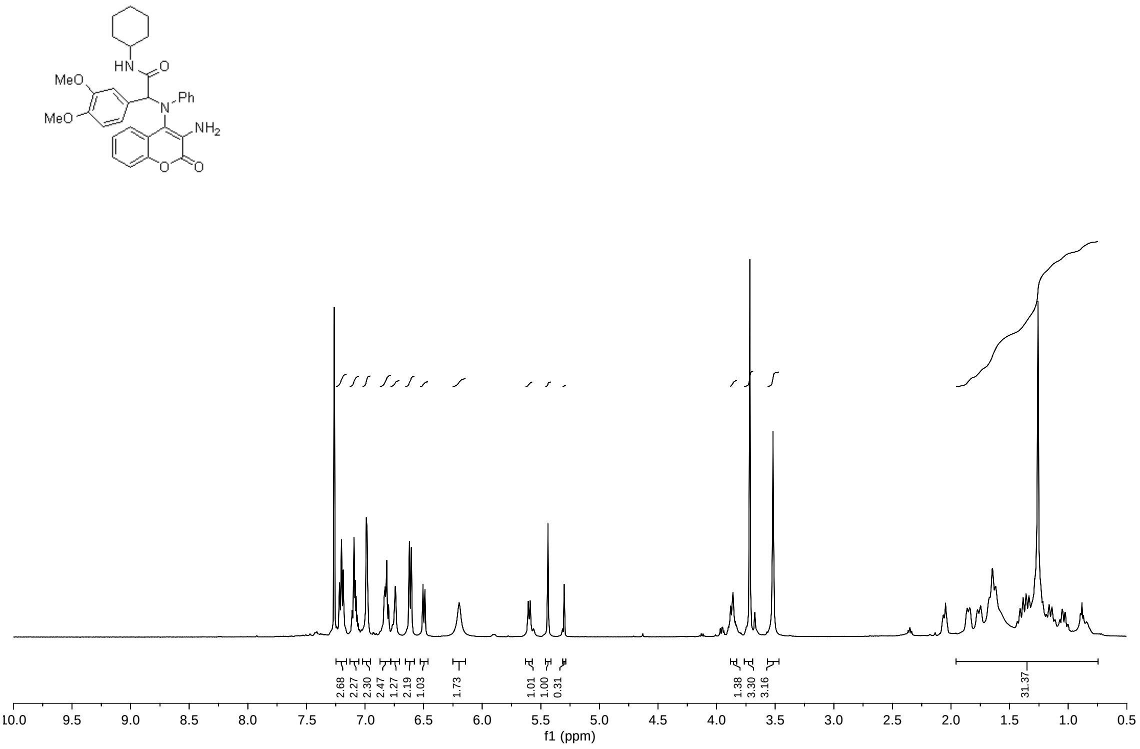

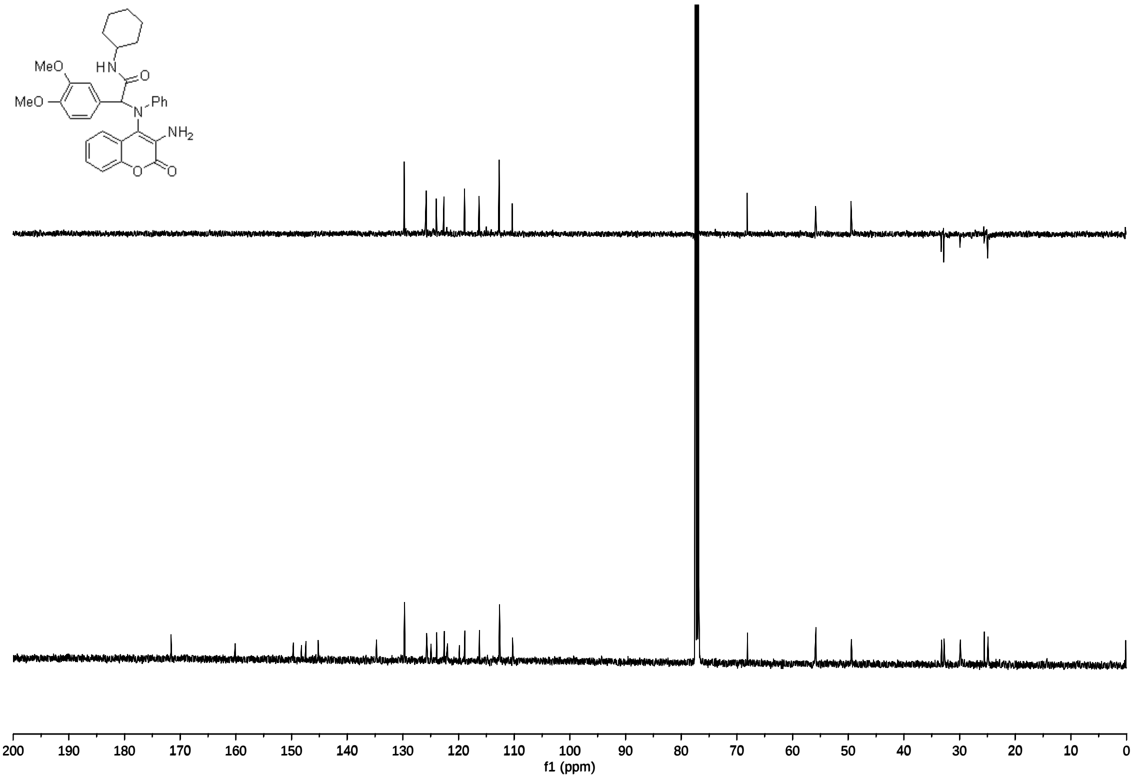

1,2-Diphenyl-1,4-dihydro-2*H*-chromeno[3,4-*b*]pyrazine-3,5-dione (19j).

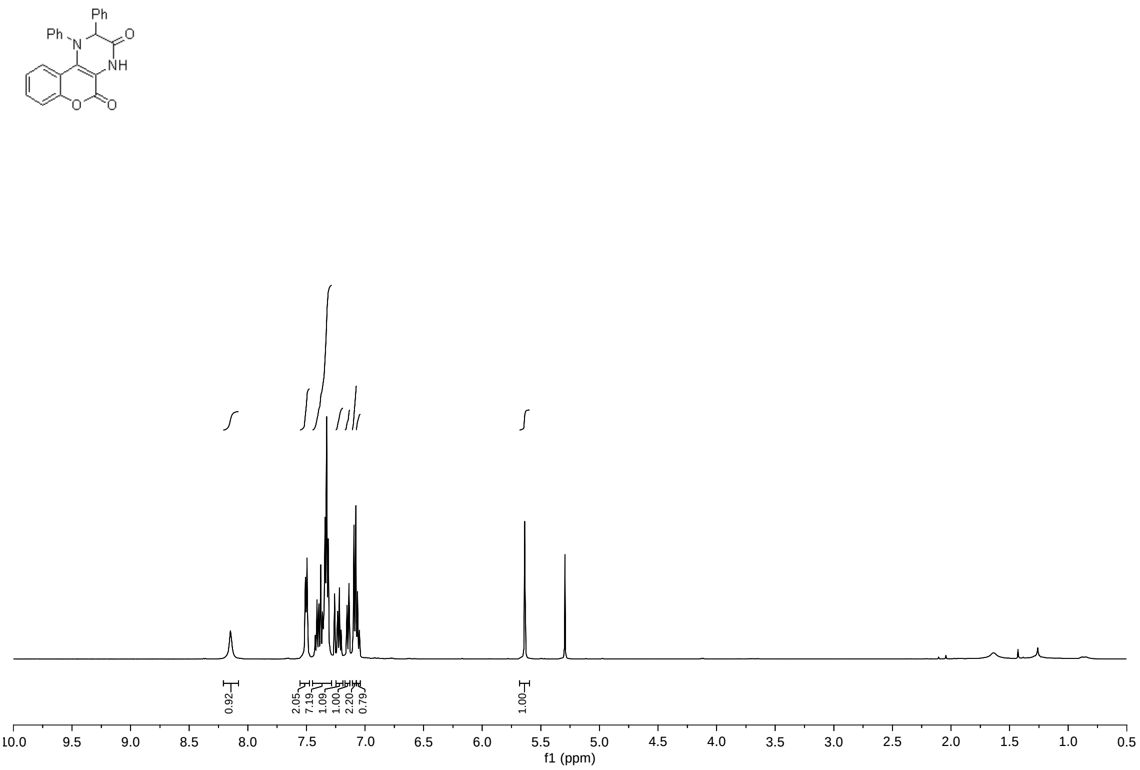

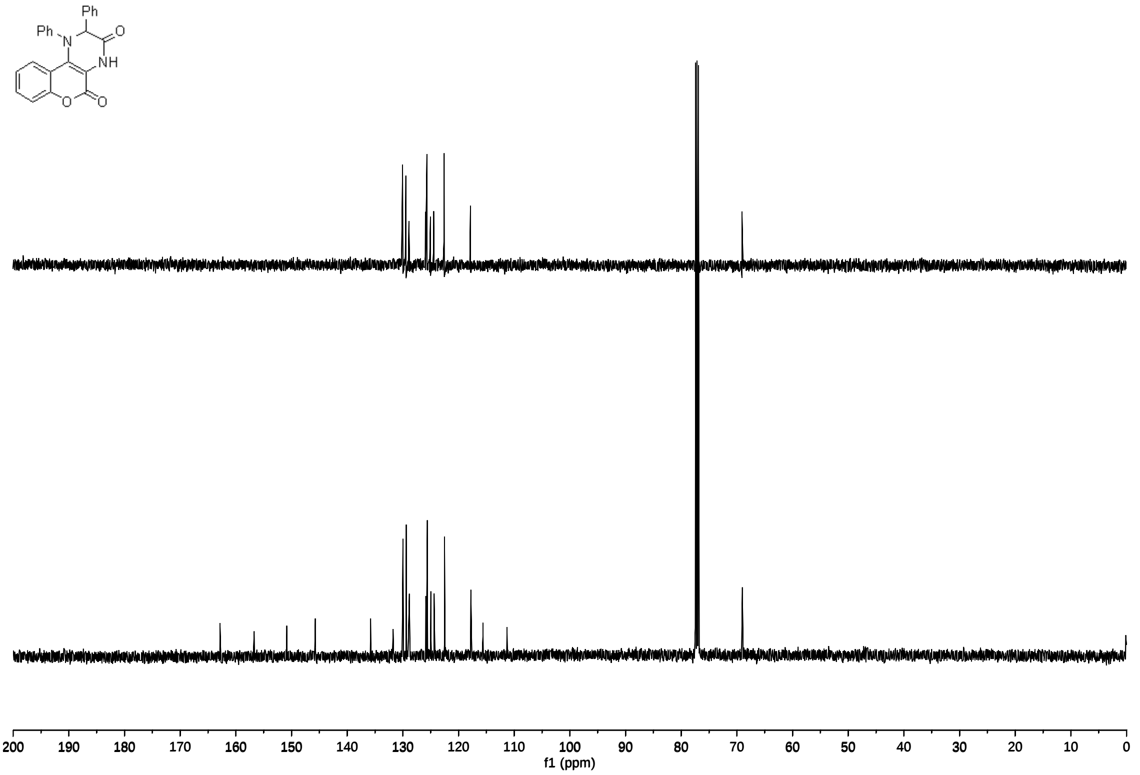

2-(3,4-Dimethoxyphenyl)-1-phenyl-1,4-dihydro-2*H*-chromeno[3,4-*b*]pyrazine-3,5-dione (19k).

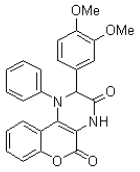

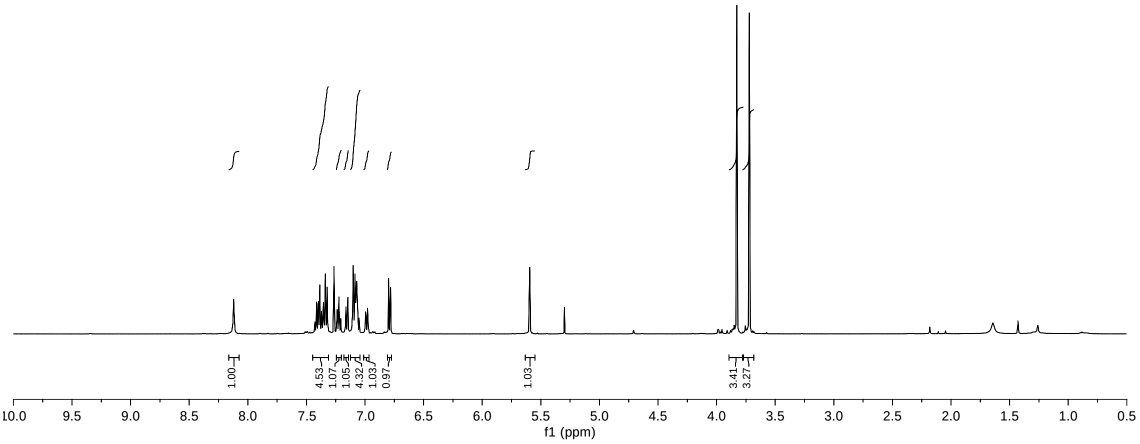

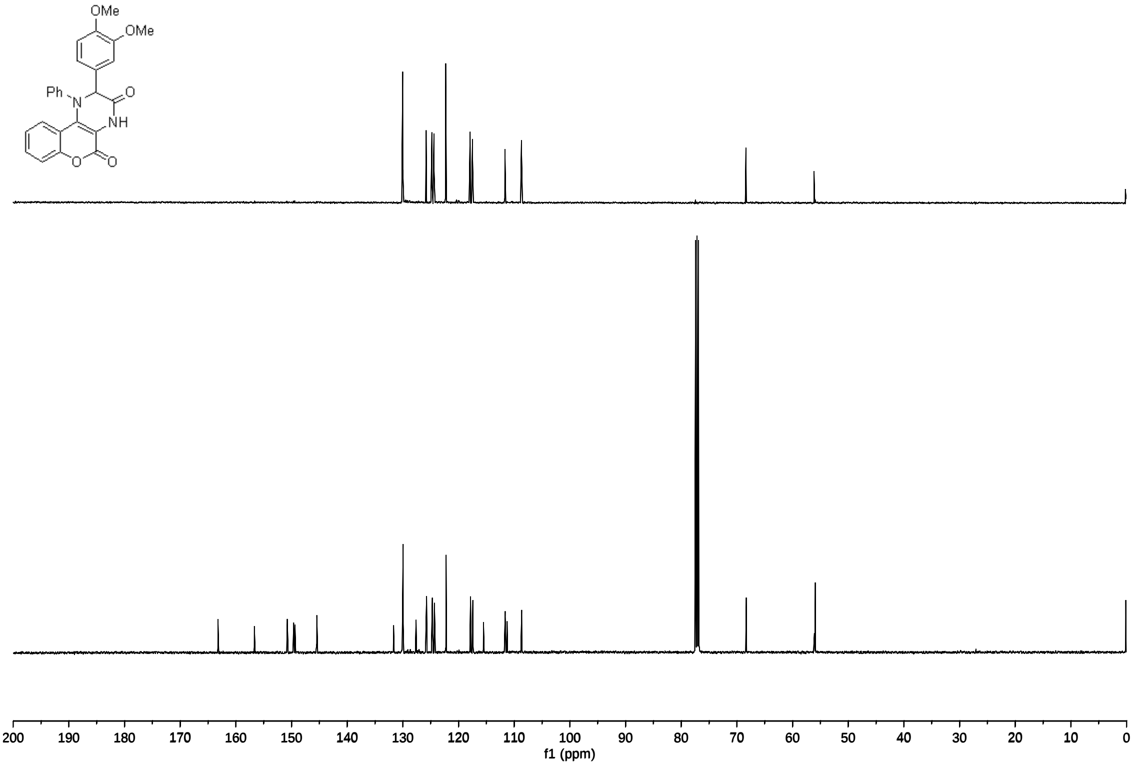

***N*-cyclohexyl-2-(3,5-dioxo-3,4-dihydro-2*H*-chromeno[3,4-*b*]pyrazin-1(5*H*)-yl)-2-phenyl acetamide (20l)**

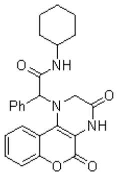

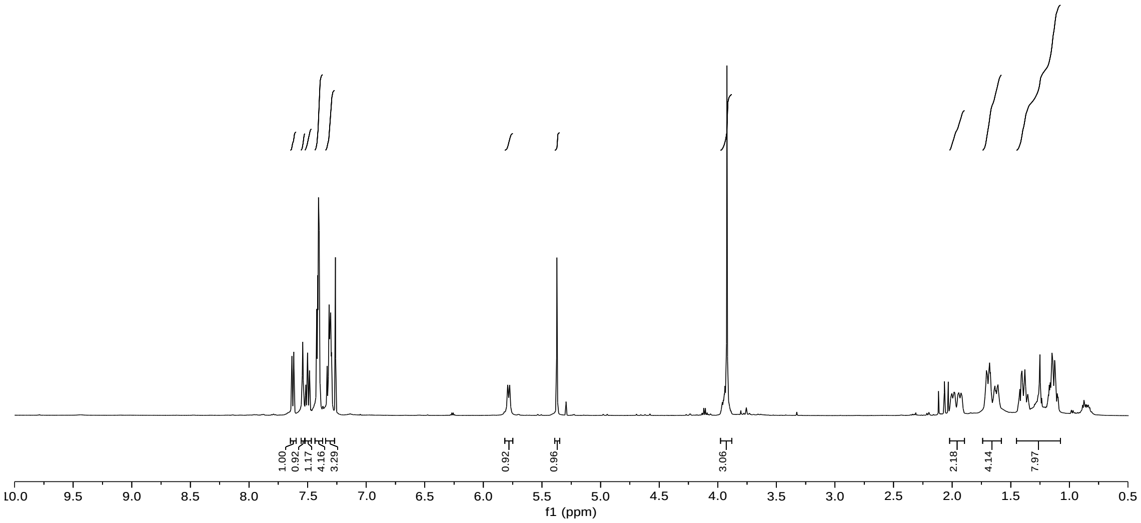

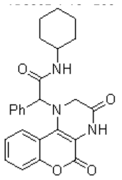

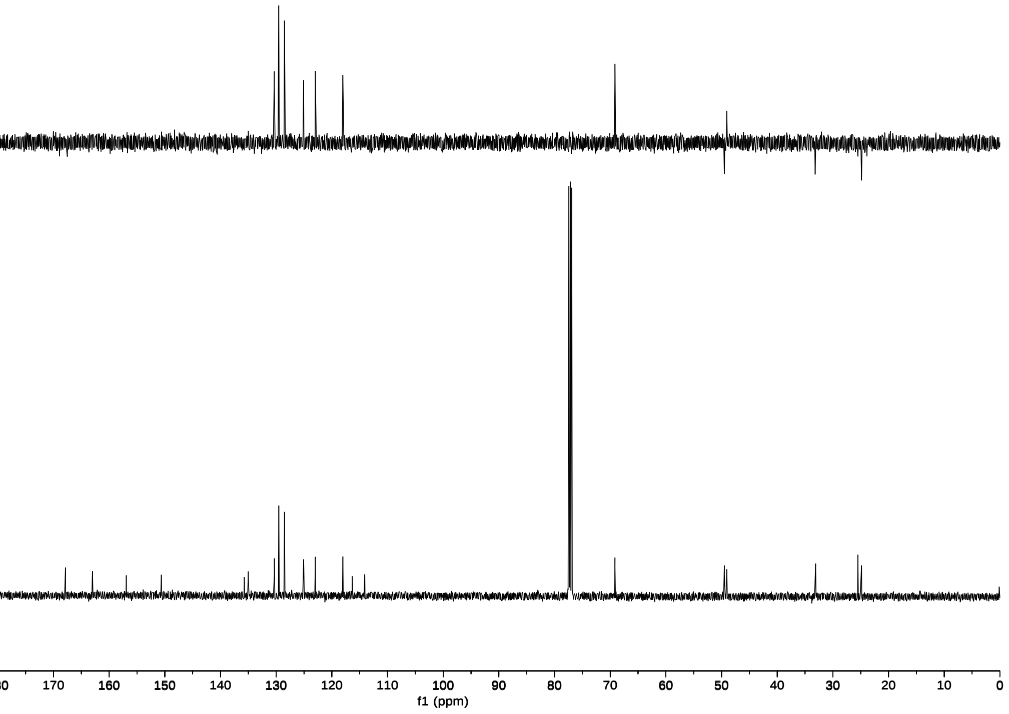

***N*-cyclohexyl-2-(3,5-dioxo-3,4-dihydro-2*H*-chromeno[3,4-*b*]pyrazin-1(5*H*)-yl)-2-(*p*-tolyl)acetamide (20m).**

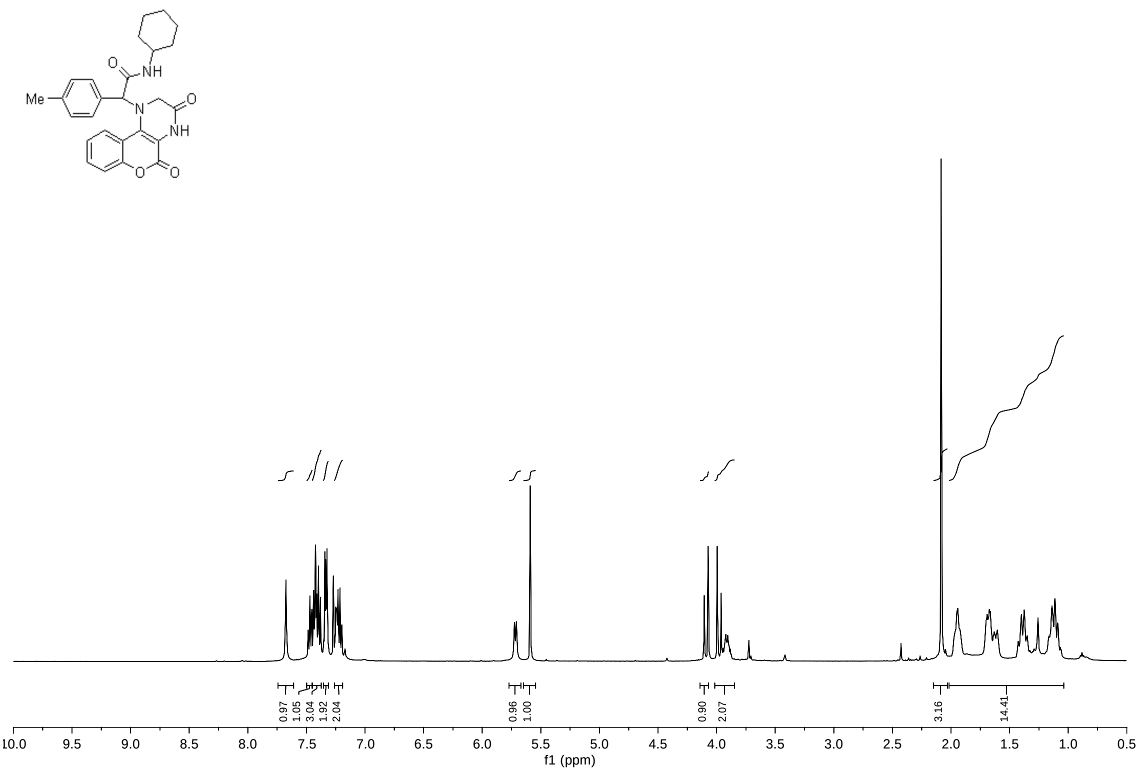

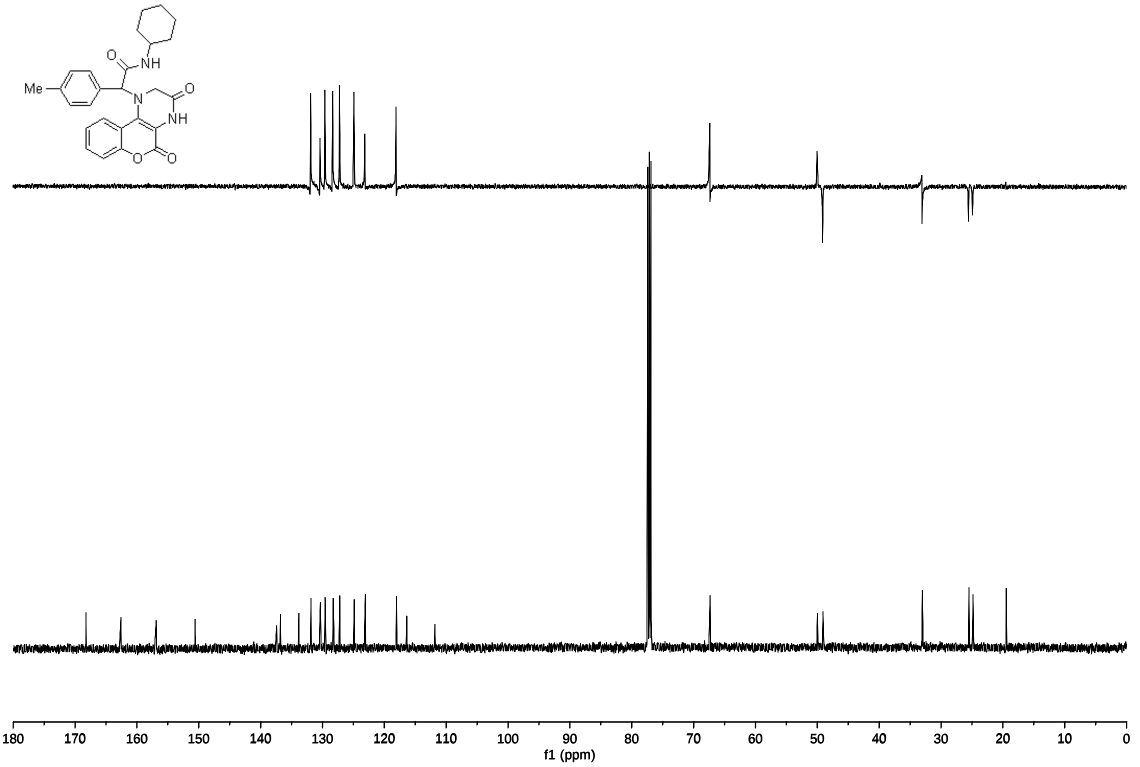

***N*-cyclohexyl-2-(3,5-dioxo-3,4-dihydro-2*H*-chromeno[3,4-*b*]pyrazin-1(5*H*)-yl)-2-(4-(trifluoromethyl)phenyl)acetamide (20n).**

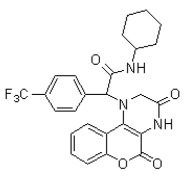

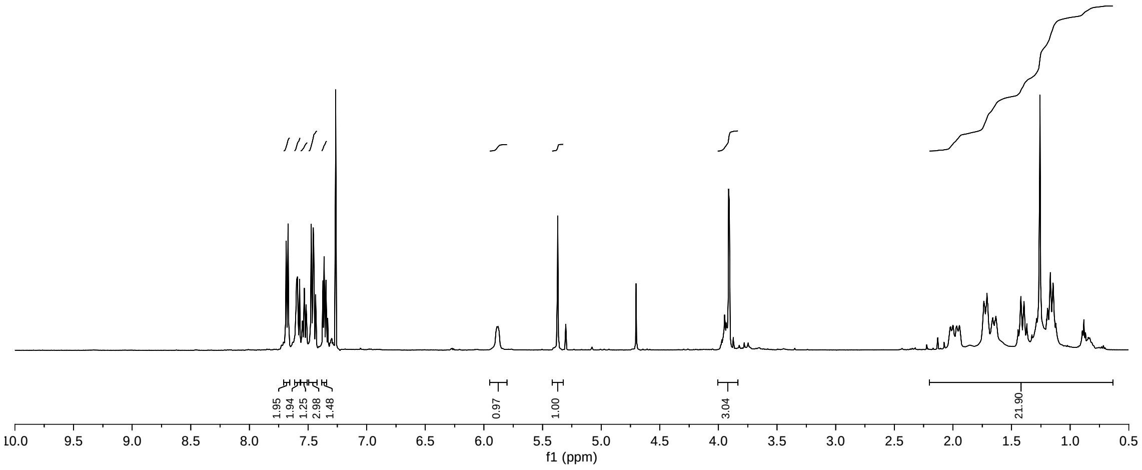

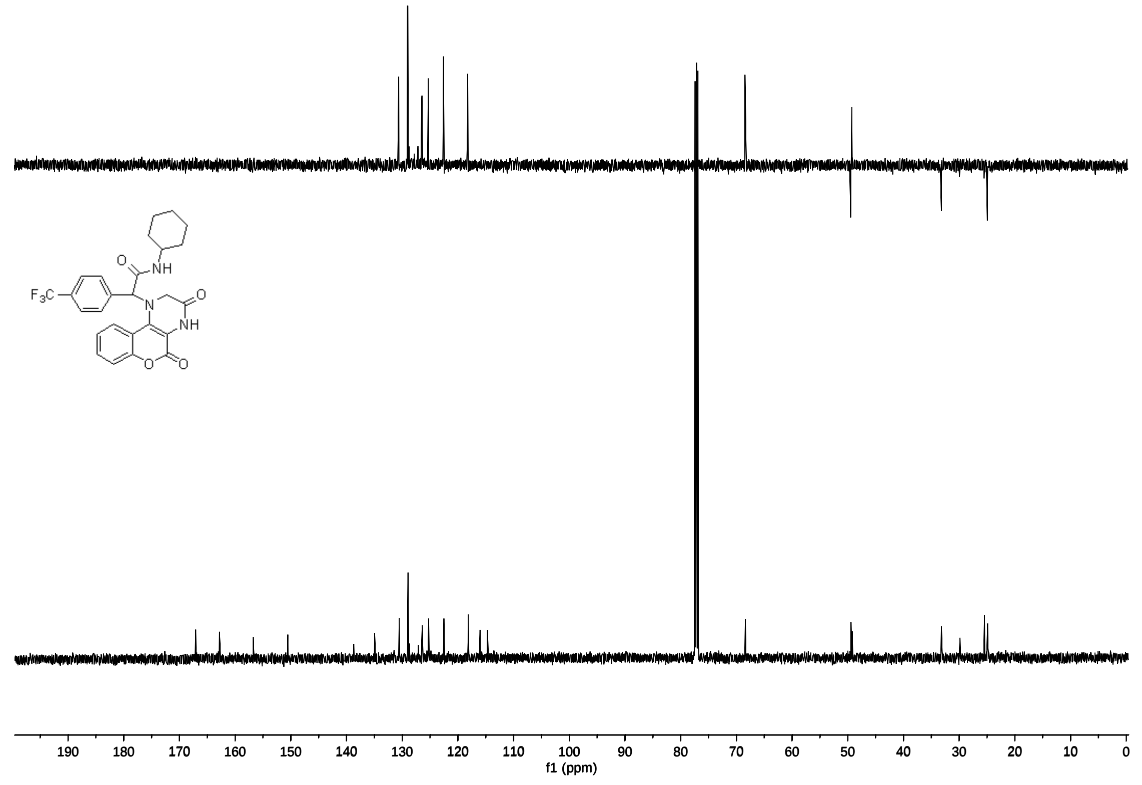

***N*-(*tert*-butyl)-2-(3,5-dioxo-3,4-dihydro-2*H*-chromeno[3,4-*b*]pyrazin-1(5*H*)-yl)-2-phenylacetamide (20o).**

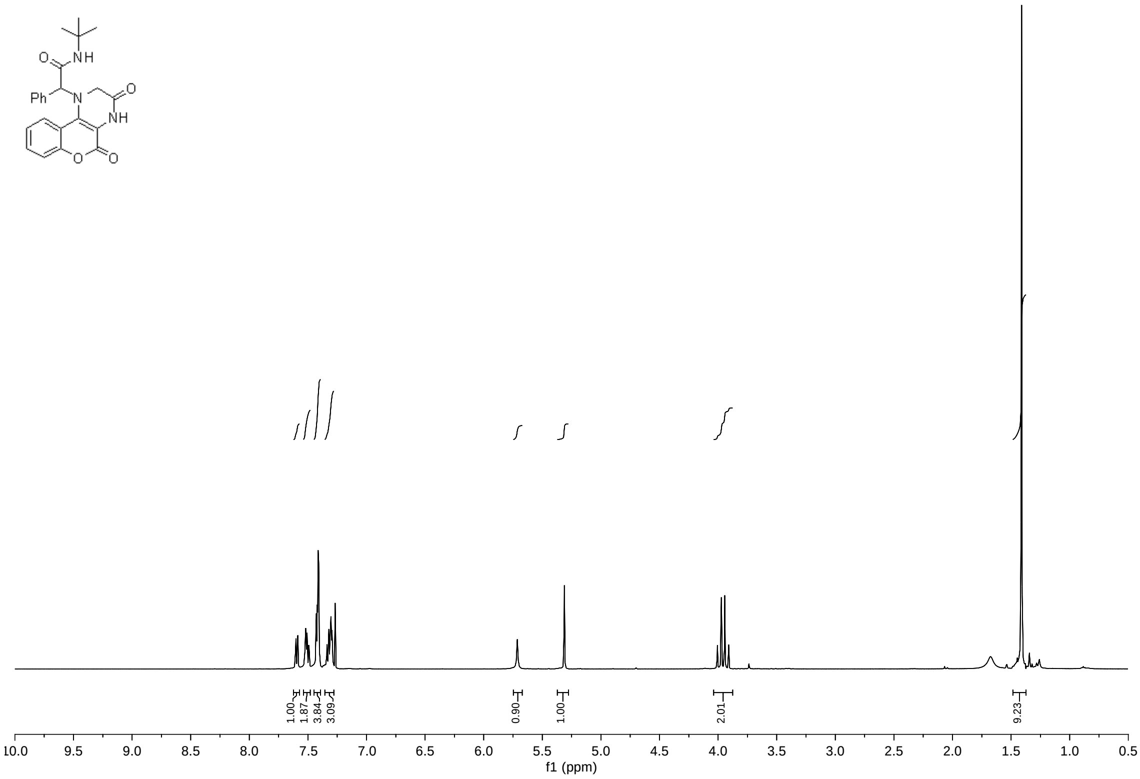

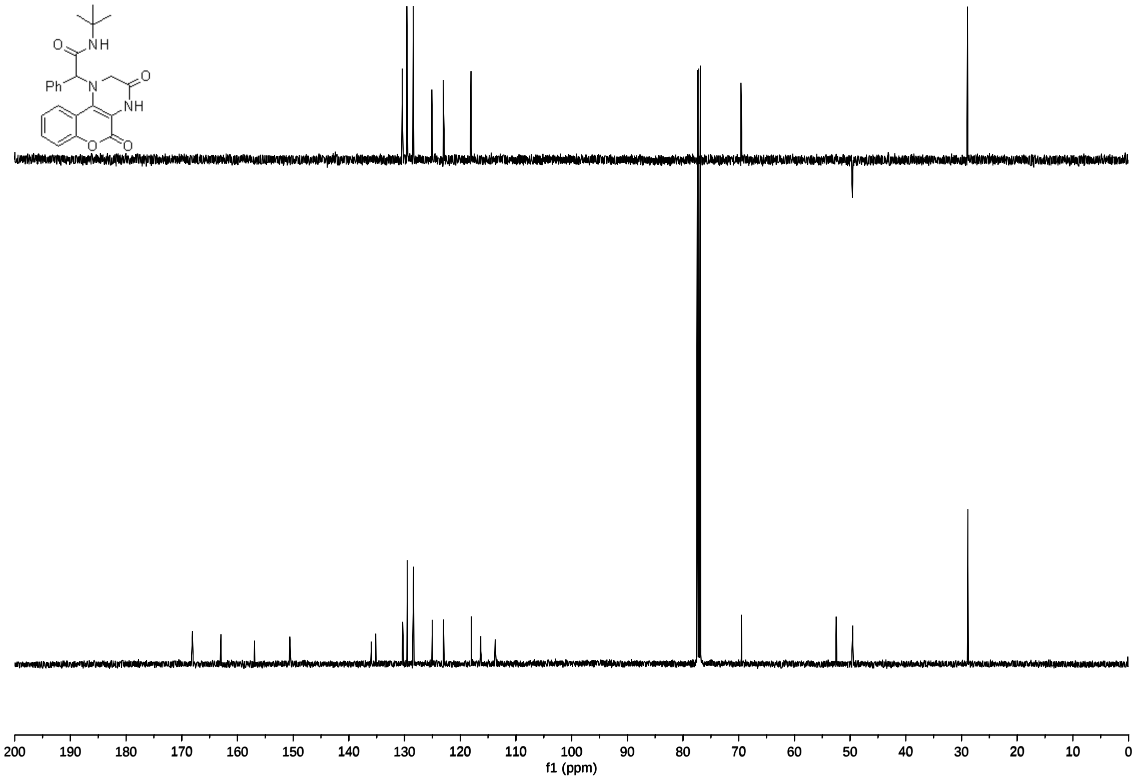

2-(3,5-Dioxo-3,4-dihydro-2*H*-chromeno[3,4-*b*]pyrazin-1(5*H*)-yl)-*N*-pentyl-2-phenylacetamide (20p).

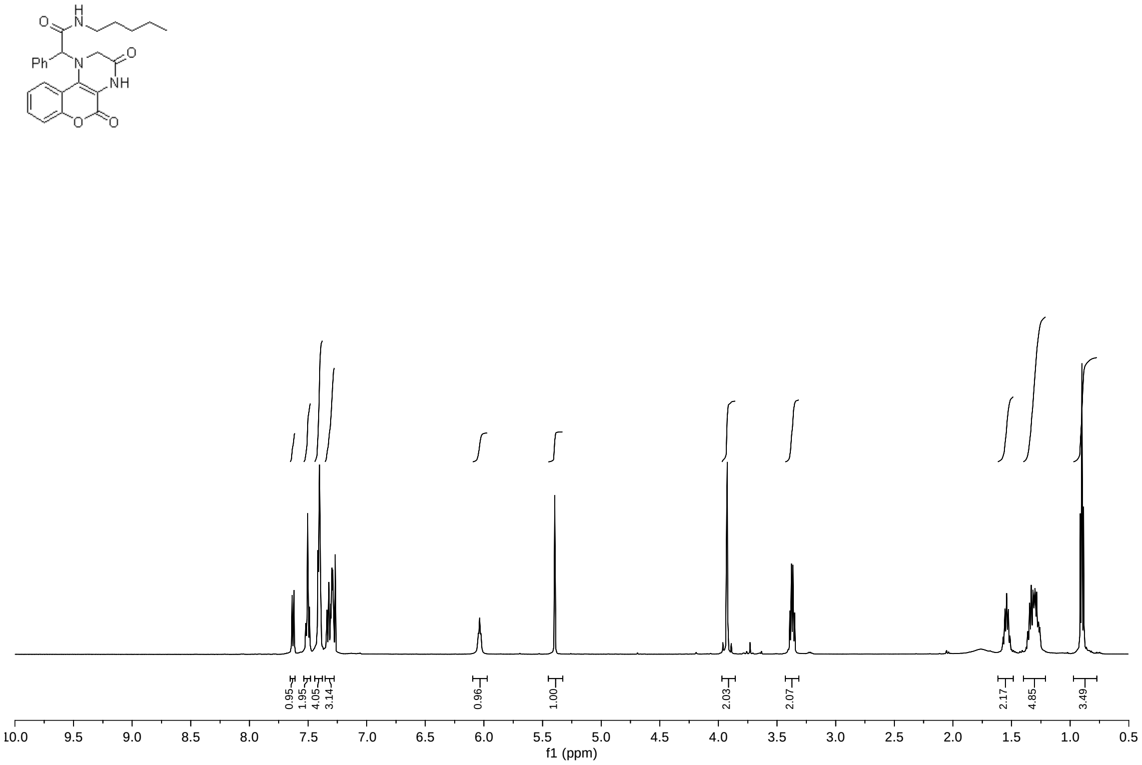

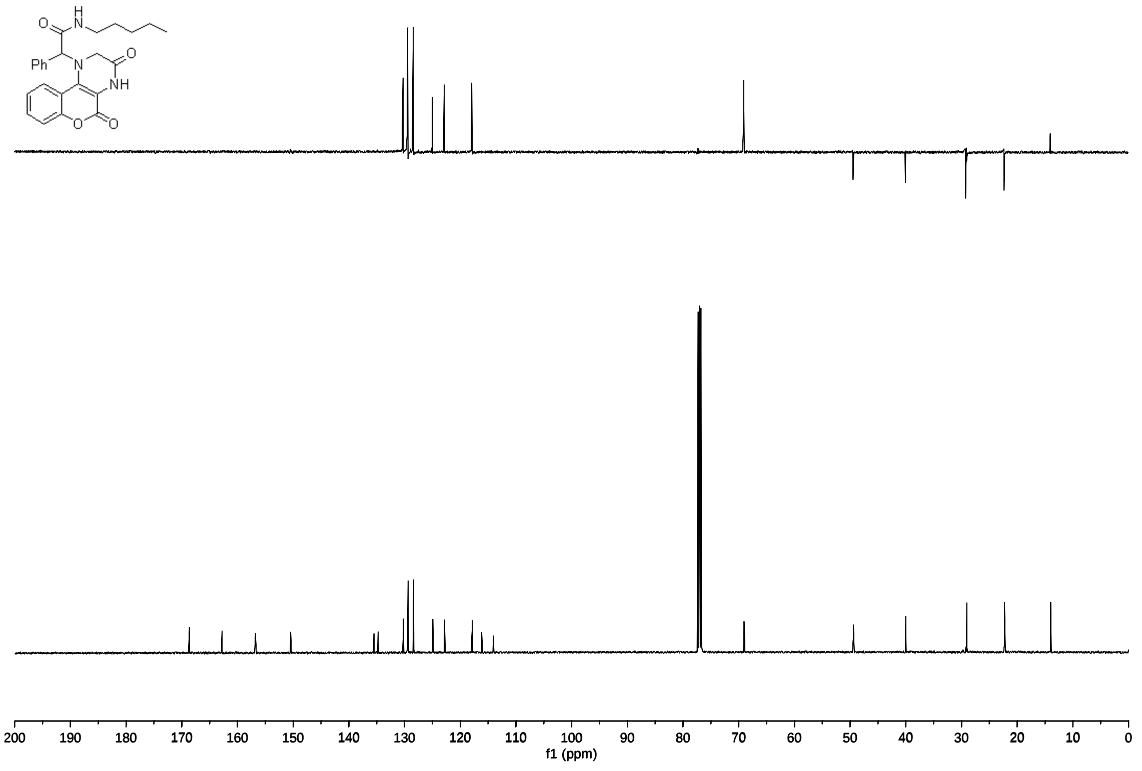

***N*-benzyl-2-(3,5-dioxo-3,4-dihydro-2*H*-chromeno[3,4-*b*]pyrazin-1(5*H*)-yl)-2-phenylacetamide (20q).**

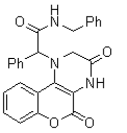

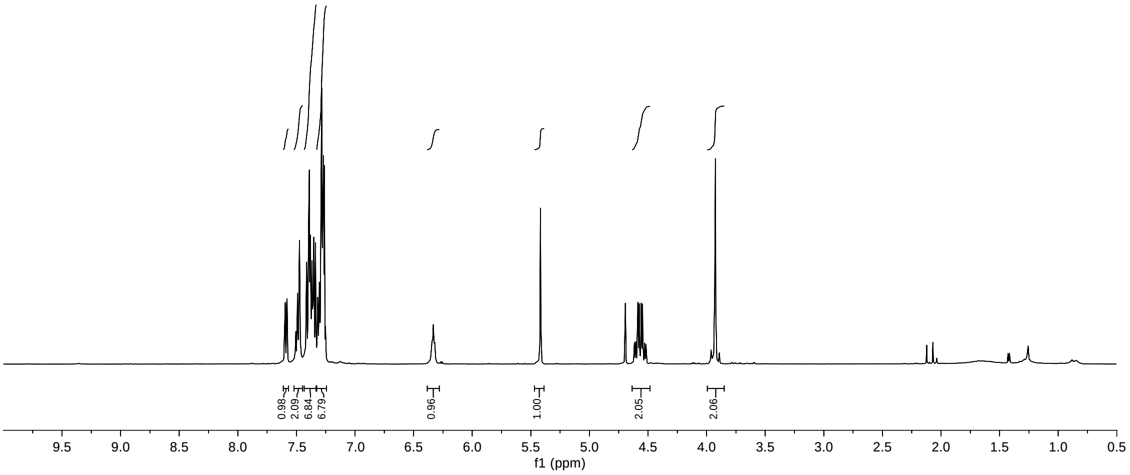

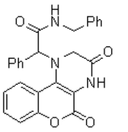

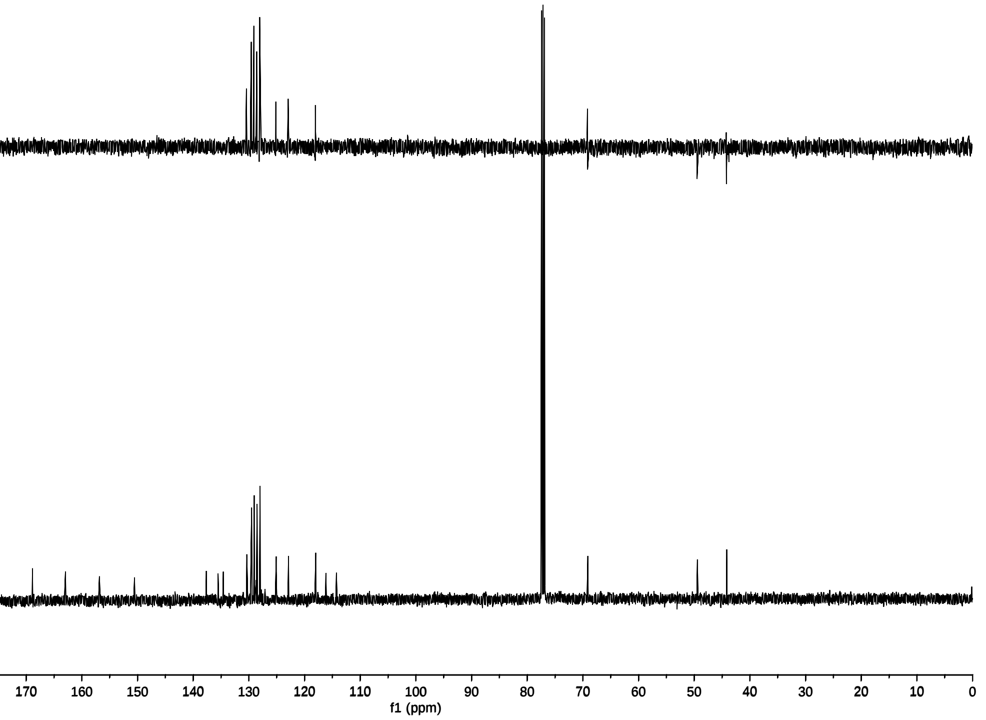

***N*-benzyl-2-(3,5-dioxo-3,4-dihydro-2*H*-chromeno[3,4-*b*]pyrazin-1(5*H*)-yl)-2-(*p*-tolyl)acetamide (20r).**

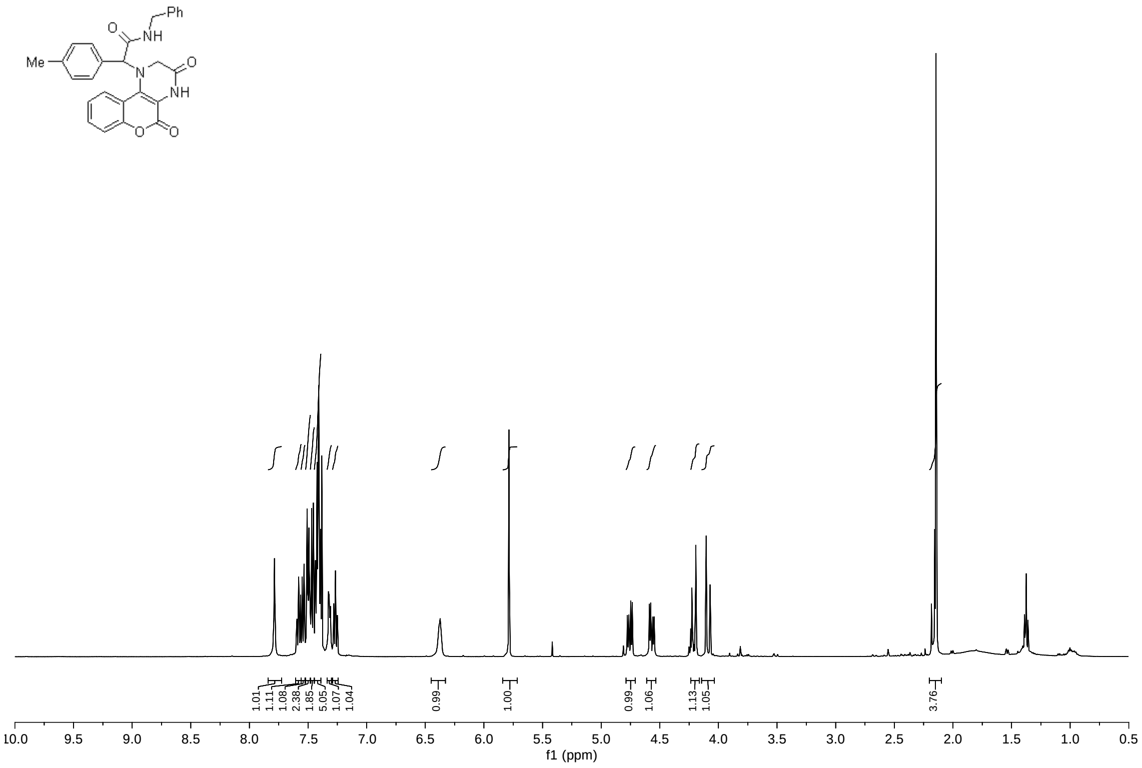

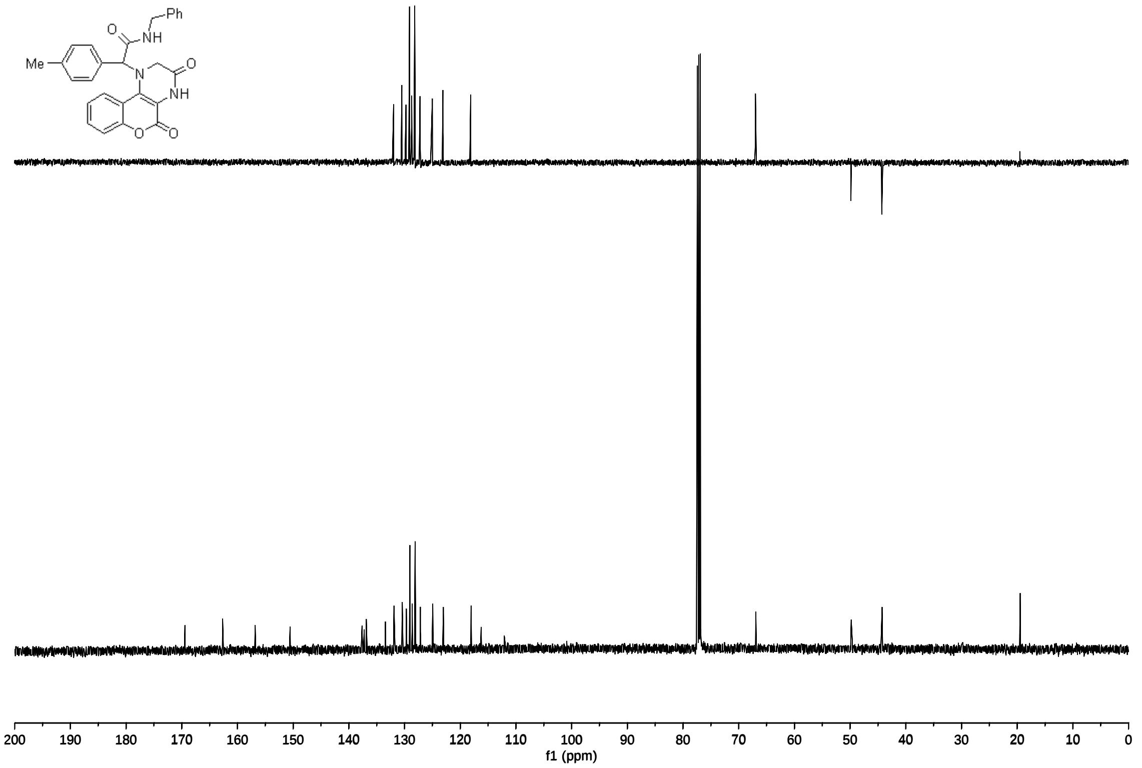

Ethyl 3-(3,5-dioxo-2-phenyl-3,4-dihydro-2*H*-chromeno[3,4-*b*]pyrazin-1(5*H*)-yl)propanoate (19s).

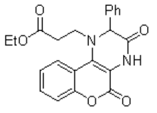

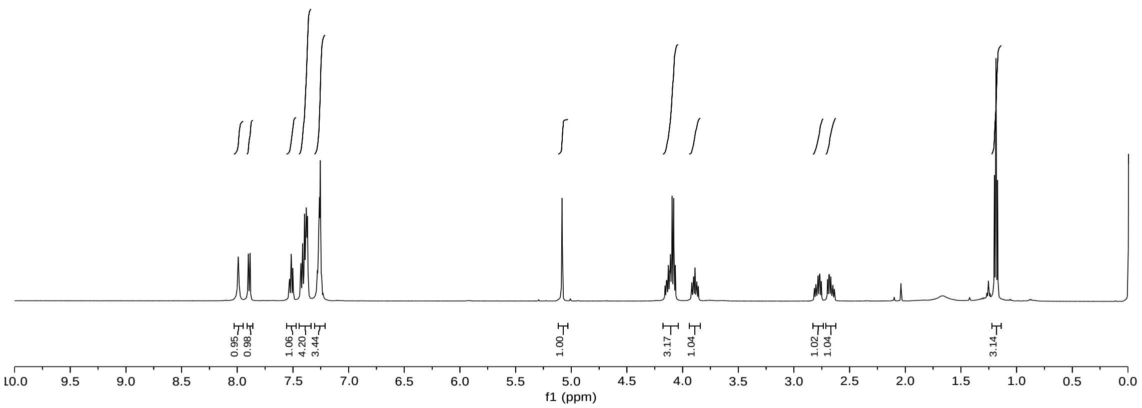

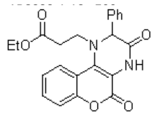

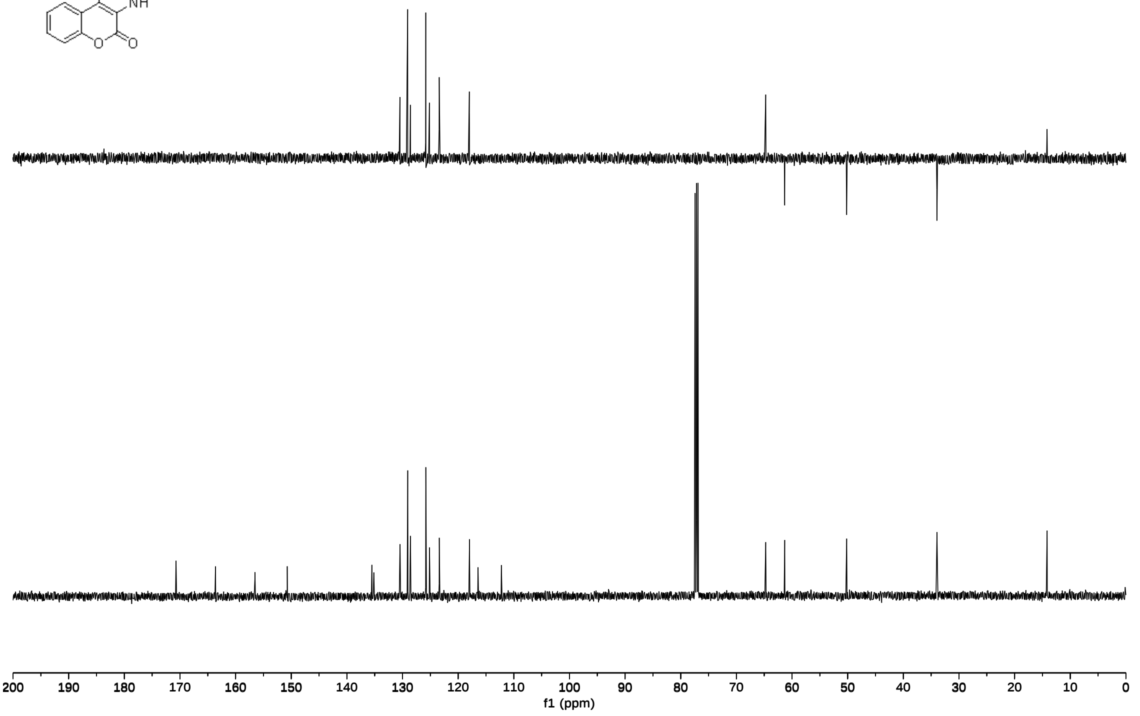

Supplement: Supplementary file 1 [file molecules-26-01287-s001.pdf]
